# ntRoot: computational inference of human ancestry at scale from genomic data

**DOI:** 10.1093/bioadv/vbaf287

**Published:** 2025-11-09

**Authors:** René L Warren, Lauren Coombe, Johnathan Wong, Parham Kazemi, Inanc Birol

**Affiliations:** Canada’s Michael Smith Genome Sciences Centre, BC Cancer, Vancouver, V5Z 4S6, Canada; Canada’s Michael Smith Genome Sciences Centre, BC Cancer, Vancouver, V5Z 4S6, Canada; Canada’s Michael Smith Genome Sciences Centre, BC Cancer, Vancouver, V5Z 4S6, Canada; Canada’s Michael Smith Genome Sciences Centre, BC Cancer, Vancouver, V5Z 4S6, Canada; Canada’s Michael Smith Genome Sciences Centre, BC Cancer, Vancouver, V5Z 4S6, Canada; Department of Medical Genetics, University of British Columbia, Vancouver, V6H 3N1, Canada

## Abstract

**Motivation:**

Ancestry information is essential to large cohort studies but is often unavailable or inconsistently measured. For studies involving genome sequencing, existing ancestry prediction methods are constrained by computational demands and complex input requirements. Efficient, scalable approaches are needed to infer ancestry directly from sequencing data while maintaining accuracy and reproducibility.

**Results:**

We present ntRoot, a computationally lightweight method for inferring human super-population-level ancestry from whole genome assemblies or short or long sequencing data. Utilizing a reference-guided, alignment-free single nucleotide variant detection framework, ntRoot employs a succinct Bloom filter to efficiently query diverse genomic inputs against a variant reference panel with known genotypes and ancestry. Demonstrated on over 600 human genome samples, including complete genomes, draft assemblies, and 280 independently generated samples, ntRoot accurately predicts geographic labels and shows high concordance with traditional methods such as ADMIXTURE (*R*^2^ = 0.9567) when estimating ancestry fractions. Analyses complete within 30 minutes for assemblies and 75 min for 30-fold sequencing data using 13–68 GB of memory. ntRoot provides global and local ancestry inference, delivering high-resolution predictions across genomic loci. This paradigm fills a critical gap in cohort studies by enabling rapid, resource-efficient, and accurate ancestry inference at scale, advancing ancestry characterization in genomic research.

**Availability:**

ntRoot is freely available on GitHub (https://github.com/bcgsc/ntroot).

## 1 Introduction

Our ancestry information, encoded within our DNA, is crucial for genomic studies. Individuals from different ancestries may have a different genetic makeup and associated disease risk factors, yet association studies do not always take ancestry into consideration or simply leave it as a confounding variable, either because that information is costly to compute, is based on incomplete genotype data, or is not reliably measured. On a population scale, the HapMap Project (Gibbs *et al.* 2003) was the first to profile common patterns of variation between individuals. Subsequently, the 1000 Genomes Project (1kGP) (Auton *et al*. 2015) and the Simons Genome Diversity Project (SGDP) (Mallick *et al.* 2016) catalogued human genomic variation across the whole genomes of healthy individuals from various populations, to generate a baseline for the study of population genetics.

To systematically quantify ancestry, researchers use two complementary approaches: Local Ancestry Inference (LAI), which resolves ancestry along the genome, and Global Ancestry Inference (GAI), which provides genome-wide summaries. LAI leverages the fact that during chromosomal crossover, proximal regions of DNA are inherited together. Hence, even if an individual’s ancestors were admixed generations ago, DNA fragments may originate from a single ancestry. While GAI provides a genome-wide summary, LAI offers higher-resolution ancestry inferences (Tang *et al.* 2005).

Most existing computational approaches developed for ancestry inference are based on single nucleotide variants (SNVs), leveraging the availability of population-scale genotype data from HapMap (Gibbs *et al.* 2003) and 1kGP projects (McVean *et al.* 2012). Tools for GAI analysis include the widely-applied model-based STRUCTURE program (Pritchard *et al.* 2000), principal component analysis-based utilities Rye (Conley *et al.* 2023) and EIGENSTRAT (Price *et al.* 2006), and ADMIXTURE for likelihood model-based estimation of ancestry in unrelated individuals (Alexander *et al.* 2009). HAPAA (Sundquist *et al.* 2008) and HAPMIX (Price *et al.* 2009), both designed for LAI, use Hidden Markov Models (HMMs) and explicitly incorporate linkage disequilibrium (LD), the degree of correlation between allelic SNVs. RFMix (Maples *et al.* 2013) is an HMM-based admixture model with conditional random forest that leverages phase and LD information (Uren *et al.* 2020) for both GAI and LAI, and SNVstory (Bollas *et al.* 2024) uses machine learning-based models to compute GAI from sequencing data. Additional LAI methods include G-Nomix, FLARE, and SALAI-Net (Hilmarsson *et al.* 2021, Oriol Sabat *et al.* 2022, Browning *et al.* 2023). While these utilities have their strengths, large-scale studies and routine applications may benefit from more flexible genomic input and smoother execution to better align with current data throughputs and computational demands in the genomic era.

**Figure 1.**
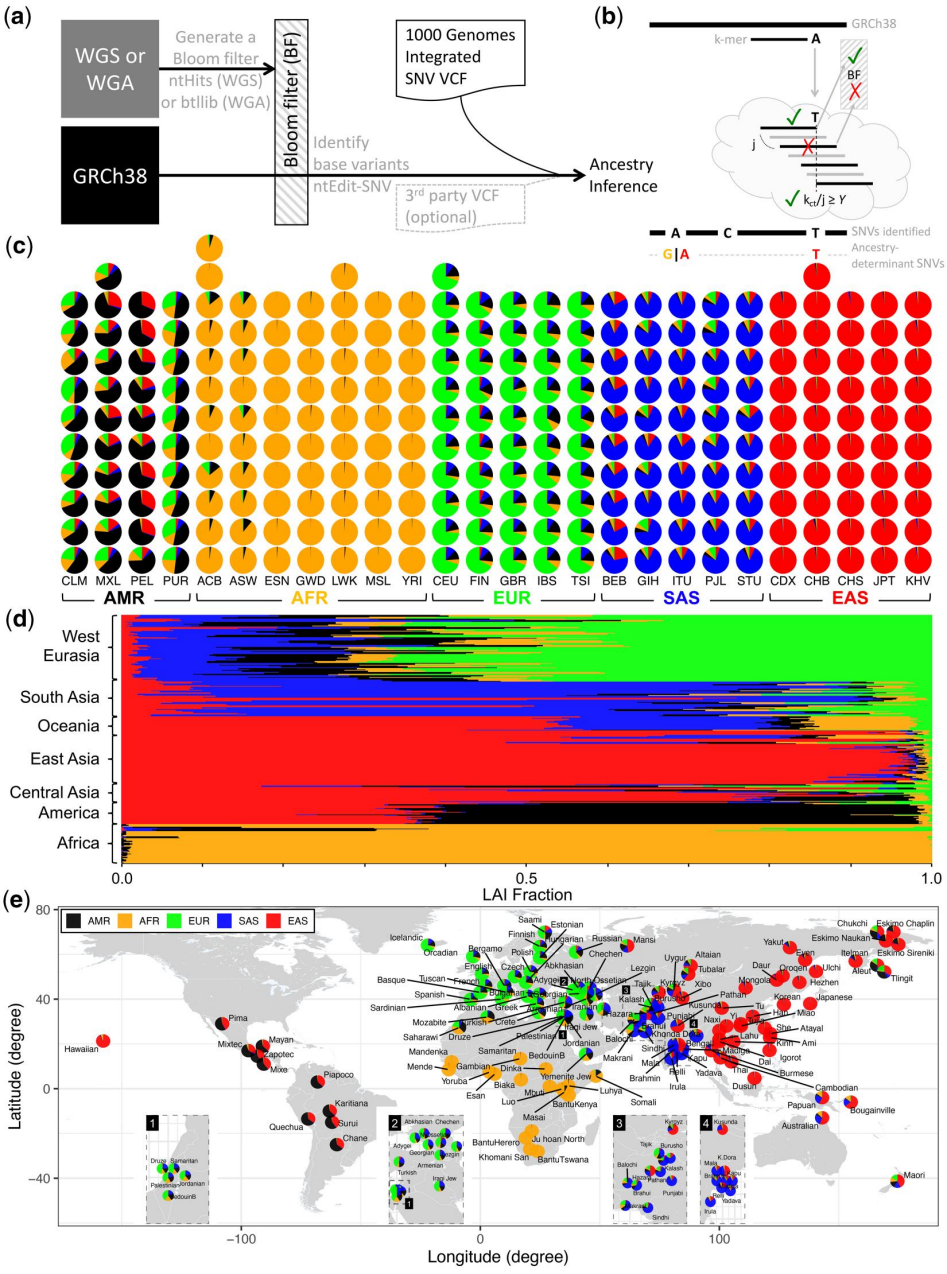
Ancestry inference from genomic data. (a) Schematic of the ntRoot framework for ancestry inference using whole-genome sequencing (WGS) or whole-genome assembly (WGA) data. ntRoot leverages ntEdit in SNV mode (ntEdit-SNV) to interrogate each base of the GRCh38 human genome reference against a Bloom filter (BF) constructed from an individual’s WGS or WGA data (using ntHits or btllib, respectively). Candidate SNVs detected by ntEdit-SNV are then cross-referenced within ntRoot against the 1kGP IVC (Zheng-Bradley *et al.* 2017, Lowy-Gallego *et al.* 2019). Optionally, ntRoot can also incorporate a third-party VCF (dashed lines) for cross-referencing against 1kGP to support ancestry inference (not presented in this study). (b) Overview of the ntRoot ancestry-determinant SNV detection algorithm. Using k-mers (e.g. black line ending in A) sliding along GRCh38 (top, black line), ntEdit v2 queries each base by testing k-mer presence (green checks) or absence (red crosses) in the BF. For each alternate allele (e.g. T), overlapping k-mers spanning the base (cloud) are queried while skipping forward in increments of j bases. A variant is confirmed when the fraction of successful BF hits (k_ct_/j) meets or exceeds the user-defined threshold −Y (default 0.55). SNVs cross-referenced with the 1kGP IVC (e.g. red A and T) are depicted. Human ancestry composition estimates derived from LAI on (c), the 266 WGS 1kGP *validation set* grouped by 5 super-populations (1kGP continental ancestry labels AMR: Admixed American; AFR: African; EUR: European; SAS: South Asian; EAS: East Asian) and 26 populations *(*1kGP labels CLM: Colombian; MXL: Mexican-American; PEL: Peruvian; PUR: Puerto Rican; ACB: African-Caribbean; ASW: African-American South West; ESN: Esan; GWD: Gambian; LWK: Luhya; MSL: Mende; YRI: Yoruba; CEU: CEPH (Centre d*‘*_**É**_tude du Polymorphisme Humain—Utah); FIN: Finnish; GBR: British; IBS: Spanish; TSI: Tuscan; BEB: Bengali; GIH: Gujarati; ITU: Indian; PJL: Punjabi; STU: Sri Lankan; CDX: Dai Chinese; CHB: Han Chinese; CHS: Southern Han Chinese; JPT: Japanese; KHV: Kinh Vietnamese), and (d) 279 SGDP WGS *discovery set* from 129 distinct populations (e) shown in the global context (using 1kGP continental ancestry labels), with inset regions zoomed-in.

Here, we present ntRoot, a scalable and flexible method for super-population-level (continental, as defined by 1kGP) LAI and GAI analysis from whole genome assemblies or raw genome sequencing samples. The name ntRoot reflects the method’s focus on uncovering the ancestral ‘roots’ of each genome at the nucleotide (nt) level. It uses a reference-guided, sequence alignment-free genome variant detection framework (Warren *et al.* 2019) that employs a succinct Bloom filter (Bloom 1970) data structure, which contains a reduced sequence representation (i.e. k-mer hashes). This filter is first used to identify SNVs and then leverages integrated variant call sets (IVC) from 1kGP to estimate continental ancestry based on established 1kGP labels (Fig. 1a and b). Prior work has shown that k-mers can carry signatures of population structure (Rahman *et al.* 2018), motivating their use in alignment-free approaches for global ancestry inference. Bloom filters are probabilistic data structures commonly used in bioinformatics software because they offer a low-memory alternative, enabling scalable applications in the era of large sequencing datasets (Warren *et al.* 2015, Jackman *et al.* 2017). The ntRoot paradigm offers both global and local ancestry inference, providing high-resolution predictions across genomic loci. In our manuscript, the term “ancestry” does not refer to genetic ancestry. Instead, it refers to group labels of geographical locations used to describe a population, as established by the 1kGP and in accordance to The National Academies of Sciences, Engineering, and Medicine guidelines (Committee on the Use of Race, Ethnicity, and Ancestry as Population Descriptors in Genomics Research et al. 2023).

## 2 Methods

Within ntRoot, we expanded the base-editing utility ntEdit (Warren *et al.* 2019) for SNV prediction and ancestry inference (Fig. 1a and b). This mode leverages k-mer (sequence words of length k)-based analysis to identify alternate base possibilities across genomic datasets, bypassing the error-checking step otherwise used in ntEdit for genome polishing. As with the polishing mode, the SNV calling feature of ntEdit requires a primary Bloom filter (BF) built from k-mers of a user-specified length k, derived from a sequencing data input. Regardless of the genomic sequence data type, whole genome assembly (WGA) or whole genome sequencing (WGS), the Bloom filter maintains a low false positive rate (∼0.008–0.0001) with three hash functions, as established in ntEdit (Warren *et al.* 2019). After BF construction, each and every k-mer of the provided reference sequence is queried, 5’ to 3’, and the last, 3’-most base is permutated to identify potential alternate bases at that position (Fig. 1b). Alternate bases are reported in a variant call format (VCF) file when their observed frequency surpasses a user-defined threshold. The method does not explicitly rely on paired-end read information or alignment-based positional context, focusing on k-mer presence/absence from the BF instead (details in Section 2.1). The approach is “alignment-free,” as it does not rely on sequence alignments in the traditional sense (i.e. mapping reads to a reference using scoring matrices and gap penalties). Instead, it leverages k-mer presence/absence queries in Bloom filters, thereby avoiding computationally intensive alignment. More precisely, it is “reference-guided,” since variant detection relies on querying k-mers from a reference genome against Bloom filters built from the sequencing data of each sample. After cross-referencing the putative SNVs identified with the 1kGP IVC (Zheng-Bradley *et al.* 2017, Lowy-Gallego *et al.* 2019), we average the SNV allele frequency (AF) within each of the five continental super-populations labels, globally for the genome under scrutiny, and locally for genomic windows/tiles (details in Section 2.2). We also keep an independent tally of non-zero AF SNVs at both local (per genomic window of a certain size, referred to as *tile* hereafter) and global (whole genome) levels. We rank the super-populations separately for each tile and the genome using those combined metrics, as an indicator of the most likely ancestry. We note that ntRoot can also predict ancestry from a user input VCF file generated from other sources (i.e. not just ntEdit; Fig. 1a), but this feature is not presented in the current study.

### 2.1 Single nucleotide variant detection with ntEdit v2

The functionality of ntEdit was extended to include single nucleotide variant detection. In this mode, each genomic base of the human reference genome (GRCh38), represented by individual k-mers, is interrogated in turn using the k-mer representation from the individual of interest, stored in a BF. Reference k-mers and their constituent overlapping k-mers query the BF for presence or absence. Putative SNVs are recorded when alternate 3’-end A, T, C, or G base positions on the original k-mer show sufficient support. Specifically, at any given position in the genome, each k-mer variant and its associated overlapping k-mers are queried, while shifting to the next k-mer by j bases. Once k/j k-mers have been queried, a potential SNV is recorded after evaluating the proportion of successful BF k-mer hits (k_ct_/j) and confirming that it meets or exceeds the user-defined −Y parameter (Bloom filter hit fraction threshold, default 0.55). This means that the SNV is supported when at least a fraction of Y overlapping k-mers yield Bloom filter hits. By design, ntEdit evaluates base substitutions one at a time at the 3’ end of k-mers. When two or more SNPs occur within k bases or less, they may be missed. In practice, the density of SNPs in the human genome (∼1 per 1000 bases; (Auton *et al*. 2015) makes such closely spaced SNPs relatively uncommon within the k base windows tested herein (e.g. 30–70 bp, corresponding to the Poisson-approximation expected probability of two or more SNPs in a window of **≈**4** × **10^**−**^^4^ to 2** × **10^**−**^^3^). Consequently, the likelihood of missed variants affecting global or local ancestry inference is minimal. Putative SNVs are then cross-referenced with integrated variant callsets from the 1kGP, marking the concordant positions in an output VCF file (Fig. 1b).

### 2.2 Ancestry inference workflow

The ntRoot pipeline is orchestrated using Snakemake, a reproducible workflow management system with built-in checkpoint capabilities (Mölder *et al.* 2021), which we leverage in ntRoot to coordinate execution of ntHits or btllib, ntEdit, and the ancestry assessment script. On whole genome sequencing (WGS) datasets, the ntRoot workflow first executes ntHits (v1.0.2, user-defined parameter, default *k* = 55, https://github.com/bcgsc/ntHits), which outputs a BF (Bloom 1970) of robust (non-error) read k-mers. Next, ntEdit (v2.0.0, https://github.com/bcgsc/ntEdit) loads the generated BF and, using the human genome (HG) reference GRCh38, interrogates every k-mer it comprises in turn, querying the BF for presence or absence as described above to generate the variants in VCF format. ntRoot then loads a succinct version of the 1kGP integrated variant call sets, where reported SNVs have an allele frequency (AF) of 1% or more in at least one human super-population label (EAS, AFR, EUR, SAS, or AMR). The SNVs reported by ntEdit are cross-referenced against this 1kGP call set. On genome sequence assemblies (WGA) and other 1× representations of an individual’s genome, ntRoot builds a BF using the btllib common code library (Nikolić *et al.* 2022) (ntroot −−genome). The remaining steps in WGA follow the same process as with WGS data (Fig. 1a). Once the ntEdit execution has completed, the ntEdit output VCF file is parsed by the ancestry assessment script (ntRootAncestryPredictor.pl), summing and averaging the variant AF for each of the five super-populations while tracking the number and rate of non-zero (termed nz in equations 1 and 3) AF SNVs over all cross-referenced SNVs. Separate tallies are kept for the whole genome and for each 5 Mbp tile (user-defined parameter −−tile, default 5 Mbp). Global (GAI) and local (LAI) ancestry inference scores are calculated as:


(1) $$S=\bar{AF}\times R_{nz}$$

where:


(2) $$\bar{AF}=\frac{1}{n}\sum_{j=1}^{n}{AF}_{j}$$

is the average allele frequency across all SNVs for a given label, calculated for the whole genome and locally for each tile.

Here:

nz: non-zero

AF: SNV allele frequenc*y*

*S* ∈ [0, 1], with 0 and 1 representing lowest and highest confidence, respectively.

For each super-population label, the non-zero SNV rate,*R_nz_*, is defined as:


(3) $$R_{nz}^{\mathrm{label}}=\;\frac{{nz\;AF\;\mathrm{SNV}\;\mathrm{count}}_{\mathrm{label}}}{\mathrm{total}\;nz\;AF\;\mathrm{SNV}\;\mathrm{count}}$$

GAI scores report the highest-scoring ancestry globally, while LAI scores assign the corresponding super-population label to each tile locally. Once each tile has been assigned a super-population label, the LAI fraction for each label is calculated as:


(4) $${\mathrm{LAI}\_\mathrm{fraction}}_{\mathrm{label}}=\;\frac{\sum_{i=1}^{N_{\mathrm{tiles}}}\mathrm{length}(\mathrm{til}e_{i})\;if\;{\mathrm{tile}}_{i,\mathrm{label}}=\;\mathrm{label}}{\sum_{i=1}^{N_{\mathrm{tiles}}}\mathrm{length}(\mathrm{til}e_{i})}$$

where N_tiles_ is the total number of tiles, tile_i_ denotes the i^th^ genomic tile, and tile_i, label_ indicates that tile’s assigned super-population label. LAI_fraction calculation accounts for actual tile lengths when reporting ancestry fractions. GAI and LAI scores are reported in a tab-separated values (TSV) output file (_ancestry-predictions_tile<tile size>.tsv). When the −−lai option is specified, ntRoot additionally outputs ancestry predictions and scores per tile in a separate TSV file (_ancestry-predictions-tile-resolution_tile<tile size>.tsv).

### 2.3 Data

In our study we tested ntRoot (v1.0.1) on over 600 genome samples, which we broadly separated into whole genome sequence (WGS) and whole genome assembly (WGA). The WGS set included 3 different cohorts: **validation**, **discovery** and **benchmark**. The WGS validation and benchmark sets consisted of 266 and 100 1kGP sequence samples, respectively and were used to test the accuracy and compute performance of the method, respectively. The WGS discovery dataset (*n* = 279) is independent from 1kGP and was sequenced as part of the Simons Genome Diversity Project (SGDP). The WGA sets comprised complete and assembled genomes in draft stages from both independent (e.g. HuRef, KOREF, CN1) and 1kGP sources (e.g. HG02055 and HG002). Details on the datasets, samples, software commands and key run parameters are included in Supplementary Table S1 and Supplementary Methods.

### 2.4 Circular genome representations

For all aforementioned experiments and for each human super-population, we tracked tile assignments and SNV density in TSV files for all non-zero AF SNVs reported by ntRoot and cross-referenced with the 1kGP integrated variant call set. That information was then plotted in R using circlize (Gu *et al.* 2014). All scripts developed to support our study are available on zenodo (10.5281/zenodo.10869033).

### 2.5 Benchmarking

ntRoot was mainly (WGA and 1kGP validation set) benchmarked on a server with 144 Intel(R) Xeon(R) Gold 6150 2.70 GHz CPUs with 2.1TB RAM, using 48 threads. Additionally, we used a server-class system with 144 Intel(R) Xeon(R) Gold 6254 3.1 GhZ CPUs with 2.9 TB RAM for processing the large SGDP WGS data volumes and benchmarking SNVstory and ADMIXTURE. We extracted the wall clock run time and peak memory from the output of the UNIX time command preceding all ntRoot, SNVstory and ADMIXTURE processes.

## 3 Results and discussion

### 3.1 ntRoot GAI and LAI-based predictions on the WGS validation set

**Figure 2.**
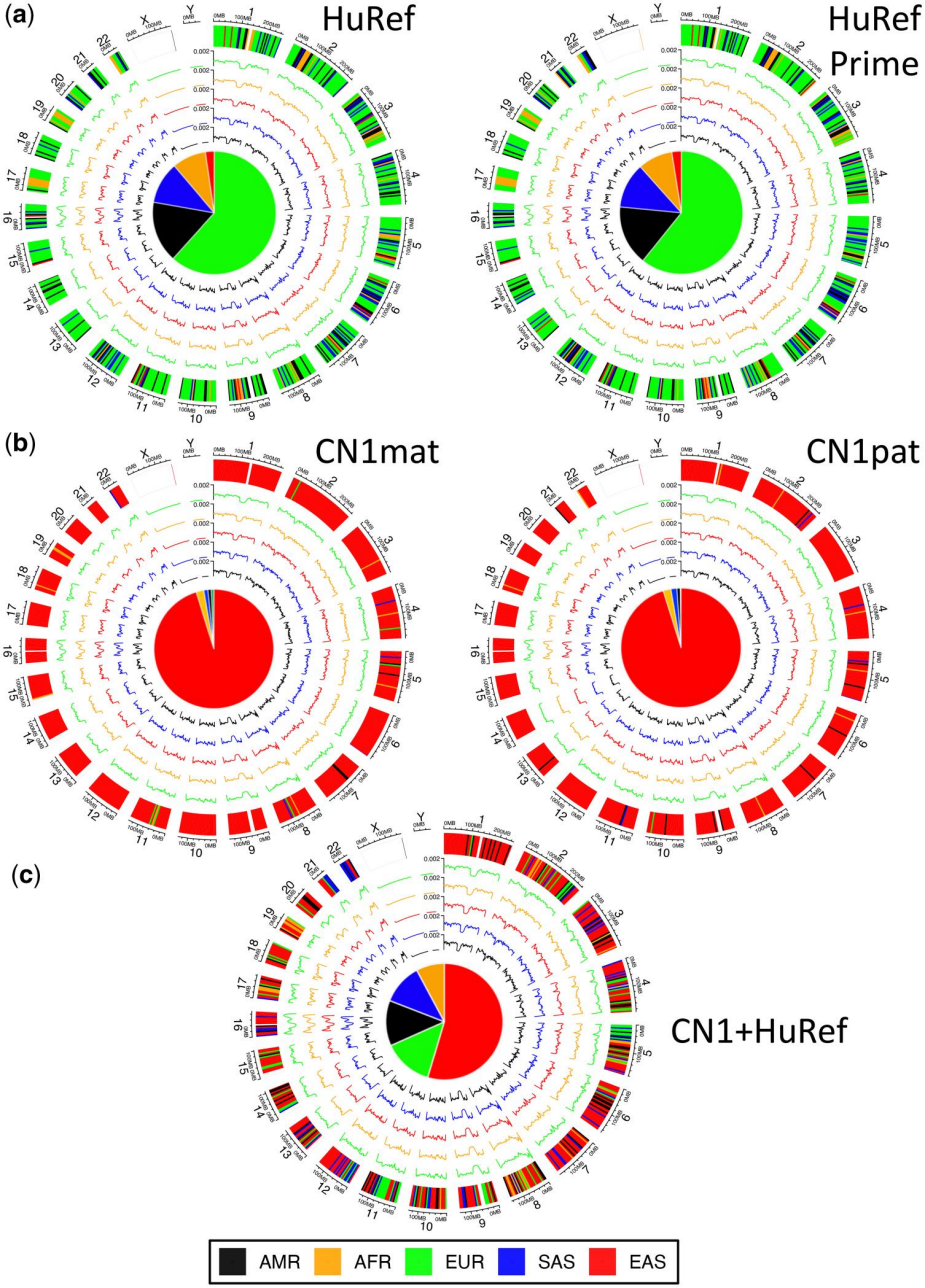
Circular genome representations of ancestry inference using WGA for (a) European HuRef (Levy *et al.* 2007) and HuRefPrime haplotypes, (b) Han Chinese CN1 (Yang *et al.* 2023) (mat: maternal, pat: paternal) haplotypes and (c) simulated HuRef/CN1 diploid rearrangement. The outer track shows ntRoot local ancestry assignments (LAI) along each chromosome using 5Mbp tiles (AMR: Admixed American; AFR: African; EUR: European; SAS: South Asian; EAS: East Asian), with each subsequent inner track showing the corresponding SNV density for each super-population. Inner pie charts report the predicted global ancestry composition (GAI).

To benchmark the performance of ntRoot, we derived super-population-level (label-based continental/geographic) ancestry for a random subset of the 1kGP whole genome sequencing (WGS) dataset (Byrska-Bishop *et al.* 2022) organized into the 26 human populations as defined by the 1kGP (**WGS validation set**, Supplementary Fig. S1 and Table S2, *n* = 266, 10–12 per population) and observed congruent ancestry inferences for all 266 individuals compared to 1kGP assigned labels when derived globally (Supplementary Fig. S2 and Tables S2–S4). The ancestry composition was inferred locally (i.e. LAI) using 5Mbp or 2Mbp tiles (Supplementary Table S5), and the estimated super-population contributions were reported for the entire genomes of the 266 and 279 WGS 1kGP and SGDP genome samples, respectively (Fig. 1c–e, Supplementary Fig. S3 and Table S6). We note that, based on LAI, the ancestry compositions of Admixed Americans (Fig. 1c and d, AMR), Europeans (EUR) and South Asians (SAS) are more diverse than are those of Africans (AFR) and East Asians (EAS), consistent with historical accounts of population movement (Willerslev and Meltzer 2021). Inferring super-population ancestries locally (i.e. LAI) is information-rich (Fig. 2). However, we caution against deriving a global ancestry label (GAI) solely from LAI (i.e. assigning the label of the largest ancestry fraction), since a small number of discrepancies arise when comparing 5Mbp and 2Mbp tiling schemes. Specifically, out of the 266 samples in the 1kGP WGS validation set, one CLM (Colombian, 0.4%) and three samples (1 CLM, 2 PUR: Puerto Rican, 1.1%) were inconsistent with their 1kGP-assigned labels when using 5Mbp and 2Mbp tiles, respectively (Supplementary Table S5). Despite these few mismatches, the overall ntRoot GAI prediction accuracy based on LAI remains very high, at 99.6% for 5Mbp tiles and 98.9% for 2Mbp tiles (precision, recall, and F1 score all >0.98; Supplementary Table S5). Interestingly, all four incongruent samples represent individuals from the Americas, where population admixture is common (Willerslev and Meltzer 2021) and hence, genetic ancestry is less homogeneous. This is the case for HG01243, a Puerto Rican individual with a recently documented (Zimin *et al.* 2022) mixed, but primarily African (AFR) ancestry, originally assigned an Admixed American (AMR) label by the 1kGP consortium. ADMIXTURE and ntRoot GAI and ancestry fractions for this individual are consistent and align with the revised and recently published AFR ancestry assignment (Zimin *et al.* 2022) (Supplementary Fig. S4, Tables S7 and S8).

### 3.2 ntRoot LAI predictions on WGA data

ntRoot LAI using the chromosome-scale diploid HuRef (Levy *et al.* 2007) (Fig. 2a), CN1 (Yang *et al.* 2023) (Fig. 2b) and haploid KOREF (Kim *et al.* 2022) (Supplementary Fig. S5) genome assemblies of three individuals revealed prevalent ancestry based on their highest composition (EUR: 61%, EAS*:* 95%, EAS*:* 95%, respectively), consistent with both the highest GAI scores, and the published European (Levy *et al.* 2007), Chinese (Yang *et al.* 2023) and Korean (Kim *et al.* 2022) ancestry for each (Tables S7 and S8). Furthermore, ntRoot correctly inferred EAS and EUR as the two most prevalent super-population ancestries from synthetic diploid genome admixtures of HuRef and CN1 (Fig. 2c, Supplementary Table S8).

### 3.3 ntRoot predictions on the WGS discovery set

Using orthogonal datasets distinct from the 1kGP data, WGS-based SGDP results (**WGS discovery set**, Supplementary Table S6) and WGA-based GAI/LAI results (Tables S7 and S8, respectively) independently validate our approach. The SGDP discovery data set uses slightly different geographic names compared to 1kGP, but despite this and the fact that we are using 1kGP integrated variant call VCFs to analyze the continental-level composition of SGDP, the location and ancestry fraction is congruent (Fig. 1e). Further, the full SGDP set comprises multiple individuals from the same populations, and the ntRoot predictions are consistent between these biological replicates (Supplementary Table S6).

### 3.4 Resource requirements, effect of sequencing coverage and key ntRoot run parameters

Unlike existing tools, ntRoot can infer ancestries directly from a genome assembly, even when it is in a draft stage (Supplementary Fig. S6). Processing haploid WGA with ntRoot averaged 29 m and consumed at most 13 GB of RAM, while it ran in under 1h36m requiring at most 68 GB of RAM, on average, on 279 SGDP WGS samples (22–87-fold genome coverage, average 44×). Despite varying WGS sequencing coverage, ntRoot produced robust predictions, using as little as 22× coverage (Supplementary Table S6). A more controlled sequencing coverage titration experiment using Illumina WGS data from the HG002 (also known as GM24385 and huAA53E0) cell line derived from an Ashkenazi Jewish individual of primarily European ancestry reveals that ntRoot’s predictions remain robust even at 12.5-fold coverage. The LAI EUR fraction, which represents the GAI and the largest ancestry fraction, shows a delta of 2.62% between full coverage (44.83%) and 12.5× coverage (42.21%), demonstrating the stability of ntRoot’s ancestry predictions at reduced sequencing depths (Supplementary Table S10). Further, the predictions appear to be sequencing data type-agnostic, as long-read WGS data from Illumina, PacBio (HiFi), and Oxford Nanopore Technologies (ONT, V14 kit) instruments yield consistent ntRoot predictions of ancestry fractions for HG002. The largest percentage variation observed is 0.7%, seen in both the LAI AFR fraction (Illumina vs. ONT) and the LAI EUR fraction (Illumina vs. ONT). This consistency is also observed in KOREF, where both Illumina and PacBio WGS data yield nearly identical ancestry fraction predictions. For example, the LAI EAS fraction, which represents the GAI and the largest ancestry fraction for KOREF, is 97.72% for Illumina and 97.90% for PacBio, with the LAI AFR, AMR, and EUR fractions showing 100% consistency across both platforms (Tables S1, S7 and S8). ntRoot ancestry predictions are influenced by the parameter k, with the GAI being accurately inferred at higher k values (e.g. k45-k70). However, as k decreases, the fraction compositions can fluctuate. This is expected, as smaller k values generally increase the recall of ancestry-discriminant SNVs, but at the cost of precision. The parameter Y, which specifies the minimum support fraction required for calling SNVs, can be tuned between 0 and 1 to emphasize higher recall or higher precision, respectively. Using the Genome in a Bottle SNV benchmarks for HG002 (Zook *et al.* 2019), we identified k = 55 and Y = 0.55 (55% threshold) as a Pareto-optimal choice, representing an empirically supported trade-off that balances sensitivity (0.8051) and precision (0.8262) in detecting ancestry-discriminant SNVs (Supplementary Fig. S7a–d, Tables S11–13). At cohort-scale, on the balanced WGS benchmark set of 100 samples, k values of 45, 55, and 65 and Y values of 0.45, 0.55 and 0.65 all produced robust ancestry predictions, with per-class Precision, Recall, F1 score, and AUC near or equal to 1 across all five major ancestry groups (EAS, SAS, AMR, AFR, EUR; Supplementary Table S13). Based on these findings, we recommend running ntRoot with the default or higher k and Y values on all datasets.

### 3.5 Benchmark set performance: comparison with other GAI/LAI methods

**Figure 3.**
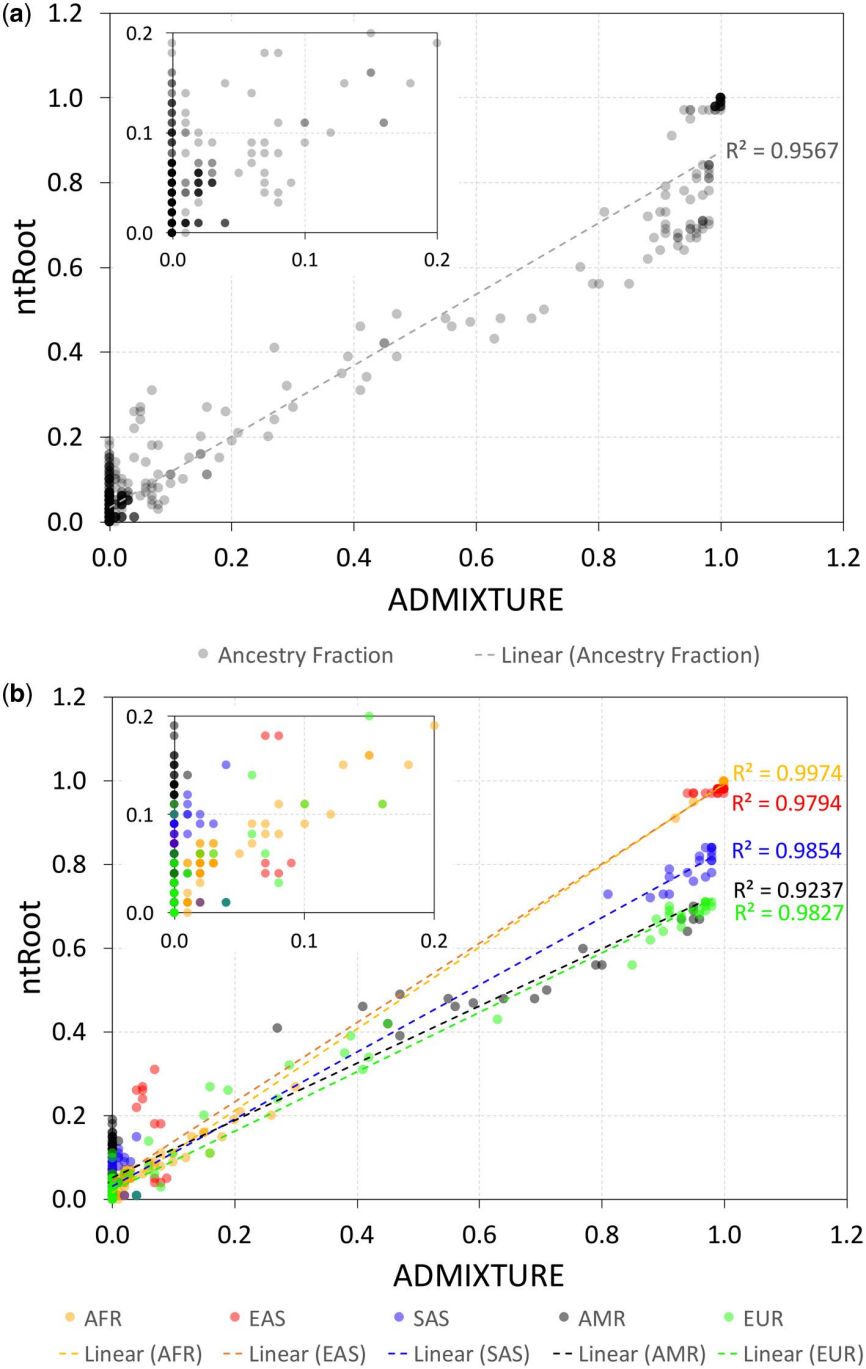
Correlation between ntRoot and ADMIXTURE ancestry fractions on a balanced set of 100 WGS 1kGP samples. Scatter plots showing a, ancestry fractions estimated by ntRoot (y-axis) against ADMIXTURE (x-axis) across all ancestries and b, for each of the five separate ancestry class: AFR (African, orange), EAS (East Asian, red), SAS (South Asian, blue), AMR (Admixed American, black), and EUR (European, green). Linear regression lines are shown for each group, with corresponding *R*^2^ values displayed. Insets show predicted ancestry fractions for each tool between fractions 0.0 to 0.2.

Compared to SNVstory (Bollas *et al.* 2024), one of the more recent GAI-based utilities shown to have more consistent performance on continental and subcontinental ancestry inference tasks compared to RFMix and ADMIXTURE, ntRoot has the same or similar accuracy as SNVstory and ADMIXTURE (100% and 99% concordance with 1kGP labels, respectively) while running ∼4× and ∼250× faster (1h09m vs. 4h25m vs. 273h42m, for ntRoot, SNVstory and ADMIXTURE, respectively) and using 1.3–6.7× less compute memory when inferring the ancestry of 100 1kGP individuals from WGS data (**WGS benchmark set**, Tables S9, S14, and S15). We note that while SNVstory outputs probabilities for GAI predictions, often 0.99 or 1 on the WGS benchmark set, these values do not provide insights into ancestry composition. In contrast, ntRoot and ADMIXTURE offer more detailed ancestry fractions, which are useful for characterizing populations and individuals with significant ancestry admixtures (Supplementary Fig. S8 and Table S14). Ancestry fraction estimates provide a more nuanced view of genetic structure, as the highest fraction does not always align perfectly with ground truth labels, and individual ancestry cannot be distilled to a single label. In support of this, ntRoot shows high concordance with ADMIXTURE overall (Fig. 3a, *R*^2^ = 0.9567) and across each ancestry (Fig. 3b), with *R*^2^ values ranging from 0.9237 (AMR) to 0.9974 (AFR).

Additionally, a mean squared error (MSE) analysis further supports this concordance, with an overall MSE of 0.78%, and individual MSE values for each ancestry as follows: EAS—0.44%, SAS—0.70%, AMR—1.19%, AFR—0.06%, and EUR—1.48%. We note, however, that ntRoot’s LAI-based fraction is different from the fine-scale genetic structure analysis previously reported (Leslie *et al.* 2015) and, in its current implementation, does not have the resolution to characterize genetic ancestry at that level. Because ntRoot cross-references the SNVs identified with an integrated variant call set collated from 2709 1kGP individuals (Zheng-Bradley *et al.* 2017, Lowy-Gallego *et al.* 2019), exact ancestry compositions are not expected. This is especially true given that the continental (and population) labels have been assigned by 1kGP based on the geographical location where the original 1kGP samples were taken/where the individuals lived at the time of collection, and that the information that ntRoot uses to cross-reference its SNVs is built on a relatively limited number (*n* = 2709) of those individuals. Given a human population size of eight billion in mid-November 2022 (United Nations, Department of Economic and Social Affairs, Population Division  2022) and that large human migrations have taken place in the past 10 000 years, 2709 individuals are not expected to represent the full breadth of nucleotide variations in humans. We also stress that ntRoot is designed to predict the likely continental (super-population)-level ancestry based on pre-assigned labels, which may or may not be representative of true genetic ancestry. To mitigate these limitations, potential strategies include providing ntRoot with an IVC built from other or additional sources (e.g. the Genome Aggregation Database—gnomAD or The International Genome Sample Resource—IGSR), ensuring it is compiled from more comprehensive and diverse reference panels (Fairley *et al.* 2020, Chen *et al.* 2024).

The ntRoot framework automates each step via a driver script, markedly simplifying the execution of ancestry prediction pipelines compared to the often convoluted workflows of other modern predictors (Supplementary Table S15). It builds on top of the reference-guided, sequence alignment-free ntEdit paradigm (Warren *et al.* 2019), for identifying and cross-referencing sequence variants in whole genome sequencing and whole genome assembly datasets with an efficient use of computational resources. It accomplishes the feat by using Bloom filters generated from either ntHits (https://github.com/bcgsc/nthits) (in WGS mode) or btllib (Nikolić *et al.* 2022) (in WGA mode). Bloom filters, widely used in bioinformatics applications and the genomics realm (Warren *et al.* 2015, Jackman *et al.* 2017, Wong *et al.* 2023), are popular, succinct probabilistic data structures that accommodate large datasets with a light compute memory footprint, as demonstrated herein. In previous work (Warren *et al.* 2019), we showed how Bloom filter false positive rates do not affect downstream ntEdit analyses. This is because ntEdit requires k-mer redundancy at each base for its nucleotide base identification. Further, ntRoot cross-references the variants it identifies (or inputs) with integrated variant call sets, which only looks at a subset of human variations characterized and curated by third parties (Zheng-Bradley *et al.* 2017, Lowy-Gallego *et al.* 2019). As observed with ntRoot predictions from WGA, even a 1× genome representation is sufficient to accurately predict continental-level ancestry. We also find that, in a typical ntRoot run that uses either WGA or WGS as input, roughly 2–3M SNVs are cross-referenced with the 1kGP integrated variant call sets and used to derive adequate ancestry inferences (Supplementary Table S7; SNV count column). The ntRoot framework is inherently scalable and could be extended to incorporate larger, more comprehensive reference datasets, such as pan-human references (Liao *et al.* 2023) or gnomAD (Chen *et al.* 2024), to improve ancestry resolution. Moreover, the methodology is not restricted to human genomes; it could be adapted for non-human species where population reference data are available. These potential extensions highlight the broader applicability and flexibility of ntRoot for diverse genomic analyses.

## 4 Conclusion

With its streamlined, memory-efficient, fast, and easy-to-execute workflow, ntRoot generates GAI and LAI-based admixture profiles that provide detailed insights into population genetics. We anticipate that ntRoot will broadly facilitate geographic-level ancestry inference, offering reliable and objective demographic information from the sequencing data of modern technologies. By enabling rapid and accurate pedigree inference at scale, ntRoot addresses a critical gap in cohort studies and holds promise for advancing super-population-level ancestry predictions for association studies in the genomic era.

## Supplementary Material

vbaf287_Supplementary_Data

## Data Availability

The data used in this study is summarized in Supplementary Material, Table S1.
