# Supplementary material for: ntRoot: computational inference of human ancestry at scale from genomic data
Source: Bioinform Adv. 2025 Nov 9;5(1):vbaf287. doi: 10.1093/bioadv/vbaf287 (PMC12695050; doi:10.1093/bioadv/vbaf287)
Supplement: vbaf287_Supplementary_Data [file vbaf287_supplementary_data.pdf]

Supplementary Material for:

ntRoot: Computational inference of human ancestry at scale from genomic data

René L Warren, Lauren Coombe, Johnathan Wong, Parham Kazemi, Inanc Birol

Table of Contents

Supplementary Methods....3

Table S1. Data Accessions. 6

Table S2. Continental, super-population level summary of ntRoot GAI results on representative 1kGP whole-genome sequencing datasets (n=266, WGS validation set) arranged by population, compared to their Assigned Label Counts....8

Table S3. Continental, super-population level summary of ntRoot GAI results on representative 1kGP whole-genome sequencing datasets (n=266, WGS validation dataset), compared to their Assigned Label Counts. 9

Table S4. ntRoot super-population level GAI summary on the 1kGP WGS validation dataset. 10

Table S5. ntRoot super-population level ancestry fraction estimates (LAI-based) on the 1kGP WGS validation set using 5 Mbp (5Mbt) and 2 Mbp (2Mbt) tiles 15

Table S6. ntRoot super-population level LAI-based ancestry fraction estimates on the SGDP WGS discovery set. 20

Table S7. ntRoot super-population level GAI summary on complete and draft genome sequences, with matching raw whole-genome read sequencing data 29

Table S8. ntRoot (unless specified) super-population level LAI-based ancestry fraction estimates on complete and draft genome sequences, with matching raw whole-genome read sequencing data 30

Table S9. Resource usage for ntRoot, SNVstory and ADMIXTURE. 31

Table S10. Effect of HG002 WGS coverage on ntRoot ancestry predictions 32

Table S11. Rationale for selecting the parameter k in ntRoot 33

Table S12. Rationale for selecting the parameter Y in ntRoot 34

Table S13. ntRoot ancestry fraction prediction results as a function of parameters k and Y on the balanced 1kGP WGS benchmark set of 100 samples....35

Table S14. Comparison of ancestry prediction results from ADMIXTURE (AdM), SNVstory (SnvS), and ntRoot (ntR) on the balanced 1kGP WGS benchmark set of 100 samples 37

Table S15. Resource usage breakdown by steps for ADMIXTURE on the balanced set of 100, 1kGP samples (WGS benchmark set)....40

Table S16. Key ntRoot input parameters....41

Figure S1. Geolocation of the 26 human populations, as defined by the 1000 Genomes Project. 42

Figure S2. ntRoot super-population level GAI compared to 1kGP labels for the 1kGP WGS validation set.......**43**

Figure S3. ntRoot ancestry composition estimates derived from LAI on SGDP WGS discovery data set from 129 distinct populations shown in the global context **44**

Figure S4. ntRoot LAI predictions on HG01243 Illumina whole genome sequencing data from 1kGP for a Puerto Rican individual of mixed, primarily African ancestry......**45**

Figure S5. ntRoot LAI predictions on KOREF......**46**

Figure S6. ntRoot LAI predictions on HG02055......**47**

Figure S7. Selection of ntRoot parameters k and Y based on precision-recall and F1 score analyses......**48**

Figure S8. a, ADMIXTURE and b, ntRoot ancestry fraction predictions (y-axis) on the balanced WGS benchmark set of 100, 1kGP samples **49**

References......**50**

Supplementary Methods

Parameter setting

Key parameters for ntRoot are summarized in Table S16, specifying which were systematically optimized, which used default values, and which are fixed by design. This table provides a transparent overview of all settings used in our analyses. Parameters such as k-mer length (−k) and the Bloom filter hit fraction threshold (−Y) were optimized using the HG002 benchmark VCF set to balance sensitivity and precision for identifying ancestry-discriminant SNVs (Figs. S7a-d and Tables S11-13). Default values, such as genome tiling size (--tile), were optimized on the validation set (Table S5), while other run parameters, including maximum threads, were used without systematic optimization. Design-fixed parameters, including the 1% allele frequency threshold used to generate the input integrated variant call set (IVC) and the Bloom filter configuration, were chosen to maintain computational efficiency and a low false positive rate.

Ancestry inference of 266 1kGP individuals, using whole genome sequencing datasets (WGS validation set)

The 30X WGS data was downloaded from https://www.internationalgenome.org/data-portal/data-collection/30x-grch38 (Table S1). We randomly selected 266 individuals, with 10-12 representatives per each 26 human populations (defined in [1,2]) and downloaded the associated WGS data with enaBrowserTools (https://github.com/enasequence/enaBrowserTools). We executed ntRoot while tracking the wall-clock run time and memory. An example ntRoot command on a 1kGP WGS dataset:

ntroot -k 55 --draft GRCh38.fa.gz --reads ERR3242308_ -t 48 -Y 0.55 -l 1000GP_integrated_snv_v2a_27022019.GRCh38.phased_gt1.vcf.gz

where GRCh38.fa.gz contains the GRCh38 human genome reference sequence with chromosome numbers as FASTA header, ERR3242308_ is the fastq file prefix for the sequencing reads and 1000GP_integrated_snv_v2a_27022019.GRCh38.phased_gt1.vcf.gz the integrated variant call set from 1kGP. We also downloaded Illumina-only (HG01243) and Illumina NovaSeq 6000 and ONT kit v14 Dorado base-called reads for NA24385 (HG002). See Table S1 for the file source and Tables S2-S5 for associated results.

Ancestry inference on 279 WGS datasets (1kGP-independent, WGS discovery set) from the Simons Genome Diversity Project

We downloaded all available 279 genomes sequencing datasets (WGS) from the diverse populations of the Simons Genome Diversity project[10] using enaBrowserTools and executed ntRoot (v1.0.1; ntroot --reads -t 48) on each read set separately while tracking the wall-clock run time and memory. We tabulated the fractions of ntRoot ancestry inference from every tsv output (in _ancestry-predictions_tile<tile size>.tsv) within the cohort and detailed in Table S6. Subsequently, we represented these fractions for a randomly-selected representative individual, for each of the distinct 129 populations represented in the main manuscript, Figure 1d. This large data served as a large-scale and independent validation of our approach.

Ancestry inference on reference, 1kGP-independent WGS datasets

In separate experiments, we downloaded the whole-genome sequencing datasets (Table S1) of an individual of European ancestry, HuRef (Illumina HiSeq X Five) and of Korean ancestry, KOREF (Illumina HiSeq X Ten / PacBio_SMRT Sequel II) and executed ntRoot (v1.0.1; ntroot --reads -t 48) on those datasets while tracking the wall-clock run time and memory. This experiment served as an independent validation of our 1kGP-based approach, since HuRef and KOREF are not part of the 1kGP cohort. See Tables S7-S8 for results.

Ancestry inferences using WGA, complete and draft whole genome sequences

In separate experiments, we downloaded the complete diploid genome assemblies (Table S1) of an individuals of European ancestry (Super-population EUR: European), HG002 (NA24385 complete genome, maternal/paternal haplotypes) and HuRef/HuRefPrime (complete genome, principal/alternate haplotypes), of Han-Chinese (EAS: East Asian) ancestry, CN1maternal/paternal (complete genome, maternal/paternal haplotypes), Korean (EAS: East Asian) ancestry, KOREF (complete genome, pseudohaploid), Puerto Rican (HG01243, AMR: Admixed American) ancestry and African (AFR: African) ancestry, HG02055 (complete genome, maternal and paternal haplotypes) and executed ntRoot (v1.0.1; ntroot --genome -t 48) while tracking the wall-clock run time and memory. We also ran ntRoot on a synthetic HuRef-CN1 admix diploid WGA and three pseudohaploid genome assemblies[11] of HG02055 to test ntRoot on admixed and pseudohaploid assemblies vs. separated, maternal and paternal haplotypes. For the synthetic HuRef-CN1 admix diploid experiment, using a custom script (available on zenodo), we first randomly selected complete paternal or maternal chromosome sequences from HuRef and HuRefPrime (random_chromosome_mix.py GCA_000002125.1_HuRef_genomic.chr-renamed.fa GCA_000212995.1_HuRefPrime_genomic.chr-renamed.fa HuRef-hap.fa), and then separately from CN1mat and CN1pat (random_chromosome_mix.py GWHCBHM00000000.genome.fasta GWHCBHQ00000000.genome.fasta CH1hap). We then executed ntRoot (v1.0.1; ntroot --genome -t 48) on the combined HuRef-hap.fa and CN1-hap.fa FASTA files. See Tables S7-S8 for results.

<u>HG002 WGS coverage titration, parameter k and Y sweeps, and sequencing data type experiments</u>

For the coverage titration experiment, we subsampled the NA24385 (HG002) Illumina WGS dataset to generate coverages ranging from 2.5 to 55-fold (using seqtk sample -s100 with read counts corresponding to 7.5 million to 165 billion bases). Each subsample was processed with ntRoot (ntroot --reads -t 48 -k 55). To evaluate the impact of parameters k and Y on ancestry predictions, we used the full ~60-fold coverage Illumina WGS data and ran ntRoot (ntroot --reads -t 48) across parameter sweeps: (1) varying k from 30 to 70 (step = 5) while fixing Y at 0.55, and (2) varying Y from 0.10 to 1.00 (step = 0.05) while fixing k at 55. The sensitivity, precision and F1 metrics were calculated at each k by first making a tally of all ancestry-discriminant SNVs from the ntRoot VCF output, and counting the number of true positives, false positives, and true negatives by comparing ntRoot's SNV predictions against a benchmark from the Genome in a Bottle Consortium[12] (available from: https://www.nist.gov/programs-projects/genome-bottle and https://ftp-trace.ncbi.nlm.nih.gov/ReferenceSamples/giab/release/AshkenazimTrio/HG002_NA24385_son/NISTv4.2.1/GRCh3

8/HG002_GRCh38_1_22_v4.2.1_benchmark.vcf.gz). Finally, in separate experiments, we ran ntRoot (ntroot --reads -t 48 -k 55) on recent PacBio Revio (Sequel II) and Oxford Nanopore (kit V14) HG002 long read datasets. See Tables S7-S8 and S10-S11 for results.

Ancestry inference of 100 1kGP individuals, using whole genome sequencing datasets (WGS benchmarking set)

The 30X WGS data was downloaded from https://www.internationalgenome.org/data-portal/data-collection/30x-grch38. We randomly selected 100 individuals in total, 20 for each of the five super-populations[1,2], ensuring no overlap with the 1kGP WGS validation set, and downloaded the associated WGS data with enaBrowserTools (https://github.com/enasequence/enaBrowserTools). We executed ntRoot (ntroot --reads -t 48) on each while tracking the wall-clock run time and memory and, in separate experiments, also tested the effect of parameters -k (45, 55, 65) and -Y (0.45, 0.55, 0.65) at cohort scale. To perform ancestry inference using SNVstory[3], we followed the approach described in the SNVstory manuscript. For each of the 100 1kGP individuals, we aligned the corresponding short reads to the human reference GRCh38 using bwa mem[77] (v 0.7.17-r1188; -t 48). Next, we generated a VCF file from the short-read alignments using the GATK[8,9] suite of tools. The necessary files for the GRCh38 reference genome were generated using samtools[10] faidx (v1.17) and gatk CreateSequenceDictionary (v4.5.0.0). Next, the variants were called using gatk HaplotypeCaller (v4.5.0.0), with separate runs launched for each chromosome (option -L), and at most 12 parallel jobs running at a given time. For each individual, all chromosome-specific VCF files generated using gatk HaplotypeCaller were merged using bcftools[10] concat (v 1.17; --threads 48). Finally, the ancestry for the individual was predicted using SNVstory (v3.0.2; --genome-ver 38 --mode WGS), providing the generated VCF file as input. The GAI inference for SNVstory was taken as the 1kGP continental prediction with the highest probability. We also inferred the ancestry of the same 100 1kGP individuals using ADMIXTURE[11]. We used the reference genotype VCF files available from the 1kGP project (https://ftp.1000genomes.ebi.ac.uk/vol1/ftp/data_collections/1000G_2504_high_coverage/working/20201028_3202_raw_GT_with_annot/), excluding the 100 1kGP query individuals. The VCF files generated for SNVstory were merged with this combined reference VCF using bcftools merge (v1.17; --threads 48). This merged VCF file was then filtered to only retain autosomes and biallelic sites using bcftools view (--threads 48 --max-alleles 2). Next, plink2[12] (v2.00a5.12LM; --threads 48 --make-bed --geno 0.1) was used to generate the required input files for ADMIXTURE from the combined VCF file. Finally, the ancestral fractions in the 100 benchmark samples were estimated using ADMIXTURE (v1.3.0; -j48 --supervised), supplying the bed file generated by plink2, and indicating the query samples using a .pop file. See Tables S9, S14-S15 for results.

Supplementary Tables

Table S1. Data Accessions.

Individual	Type	Samples	Accession	Resource URL
**GRCh38**	WGA	NA	GCA_000001405.15 and 10.5281/zenodo.10869033	https://www.ncbi.nlm.nih.gov/datasets/genome/GCF_000001405.15/
**HuRef[13]**	WGS	1	SRR8595488	https://www.ebi.ac.uk/ena/browser/view/SRR8595488
**HuRef[13]‡**	WGA	1	GCA_000002125.1	https://ftp.ncbi.nlm.nih.gov/genomes/all/GCA/000/002/125/GCA_000002125.1_HuRef/GCA_000002125.1_HuRef_genomic.fna.gz
**HuRefPrime[13]‡**	WGA	1	GCA_000212995.1	https://ftp.ncbi.nlm.nih.gov/genomes/all/GCA/000/212/995/GCA_000212995.1_HuRefPrime/GCA_000212995.1_HuRefPrime_genomic.fna.gz
**CN1[14]**	WGA maternal	1	GWHCBHM00000000	https://download.cncb.ac.cn/gwh/Animals/Homo_sapiens_CN1_v0.8_mat_GWHCBHM00000000/GWHCBHM00000000.genome.fasta.gz
**CN1[14]**	WGA paternal	1	GWHCBHQ00000000	https://download.cncb.ac.cn/gwh/Animals/Homo_sapiens_CN1_v0.8_pat_GWHCBHQ00000000/GWHCBHQ00000000.genome.fasta.gz
**HuRef/CN1 simulated diploid**	WGA	1	10.5281/zenodo.10869033	https://doi.org/10.5281/zenodo.10976332
**KOREF[15]***	WGA	1	GCA_020497085.1	https://www.ebi.ac.uk/ena/browser/api/fasta/GCA_020497085.1
**KOREF[15]**	WGS (Illumina)	1	SRR10861698	https://www.ebi.ac.uk/ena/browser/view/SRR10861698
**KOREF[15]**	WGS (PacBio Sequel II)	1	SRR14759118-21, 23-24	https://www.ebi.ac.uk/ena/browser/view/SRR14759118 to https://www.ebi.ac.uk/ena/browser/view/SRR14759118
**HG01243**	WGS	1	ERR3988858	https://www.ebi.ac.uk/ena/browser/view/ERR3988858
**NA24385 (HG002)**	WGA maternal	1	GCA_018852615.3	https://www.ebi.ac.uk/ena/browser/view/GCA_018852615.3
**NA24385 (HG002)**	WGA paternal	1	GCA_018852605.3	https://www.ebi.ac.uk/ena/browser/view/GCA_018852605.3
**NA24385 (HG002)**	WGS (llumina NovaSeq 2x250bp)	1	SRR11321732	https://www.ebi.ac.uk/ena/browser/view/SRR11321732
**NA24385 (HG002)**	WGS (ONT V14 Dorado)	1	NA	https://labs.epi2me.io/askenazi-kit14-2022-12/ and s3://ont-open-data/giab_lsk114_2022.12/
**NA24385 (HG002)**	WGS (PacBio Revio HiFi Sequel II)	1	SRR26402938	https://www.ebi.ac.uk/ena/browser/view/SRR26402938
**HG02055[1,2]**	WGS	1	ERR3988979	https://www.ebi.ac.uk/ena/browser/view/ERR3988979
**HG02055[4]***	WGA (GoldRush[*,^])	1	10.5281/zenodo.7884681	https://doi.org/10.5281/zenodo.7884681
**HG02055[4]***	WGA (Shasta[*,^])	1	10.5281/zenodo.7884681	https://doi.org/10.5281/zenodo.7884681
**HG02055[4]***	WGA (Flye[*,^])	1	10.5281/zenodo.7884681	https://doi.org/10.5281/zenodo.7884681
**HG02055[16]**	WGA maternal	1	JAHEPJ01	https://www.ncbi.nlm.nih.gov/Traces/wgs/JAHEPJ01
**HG02055[16]**	WGA paternal	1	JAHEPK01	https://www.ncbi.nlm.nih.gov/Traces/wgs/JAHEPK01
**1kGP[1,2]**	WGS	266 and 100	Tables S4/S5 and S12/S13	https://www.internationalgenome.org/data-portal/data-collection/30x-grch38

**SGDP[3#]**	WGS	279	PRJEB9586, Table S6	https://www.simonsfoundation.org/simons-genome-diversity-project/<br>https://ddbj.nig.ac.jp/resource/bioproject/PRJEB9586<br>https://www.ebi.ac.uk/ena/browser/view/PRJEB9586
**1kGP integrated variant call set**	VCF	2709	10.5281/zenodo.10869033	https://www.internationalgenome.org/data-portal/data-collection/30x-grch38

***Pseudohaploid assembly. ^Assembled with WGS Oxford Nanopore datasets[4] (Wong et al., 2022, Table 6). The mat/pat suffix indicates maternal and paternal haplotypes, respectively. ‡HuRef and HuRefPrime haploid WGAs each refer to the principal and alternate haplotypes respectively. #The primary SGDP dataset consists of data from 260 genomes from 127 populations: 39 Africans, 23 Native Americans, 27 Central Asians or Siberians, 49 East Asians, 27 Oceanians, 38 South Asians and 71 West Eurasians according to https://www.simonsfoundation.org/simons-genome-diversity-project/; We identified data (and report results in Table S5) for 279 distinct individuals organized into 129 unique populations (Figure 1d).**

Table S2. Continental, super-population level summary of ntRoot GAI results on representative 1kGP whole-genome sequencing datasets (n=266, WGS validation set) arranged by population, compared to their Assigned Label Counts.

Super-population Code	Population Code	Population Name	Assigned Label Counts	ntRoot GAI Correct Prediction Counts
**AMR**	MXL	Mexican-American	11	11
**AMR**	PUR	Puerto Rican	10	10
**AMR**	PEL	Peruvian	10	10
**AMR**	CLM	Colombian	10	10
**AFR**	MSL	Mende	10	10
**AFR**	LWK	Luhya	11	11
**AFR**	ASW	African-American South West	10	10
**AFR**	YRI	Yoruba	10	10
**AFR**	GWD	Gambian	10	10
**AFR**	ESN	Esan	10	10
**AFR**	ACB	African-Caribbean	12	12
**SAS**	STU	Sri Lankan	10	10
**SAS**	BEB	Bengali	10	10
**SAS**	ITU	Indian	10	10
**SAS**	PJL	Punjabi	10	10
**SAS**	GIH	Gujarati	10	10
**EUR**	GBR	British	10	10
**EUR**	IBS	Spanish	10	10
**EUR**	TSI	Tuscan	10	10
**EUR**	FIN	Finnish	10	10
**EUR**	CEU	CEPH*	11	11
**EAS**	KHV	Kinh Vietnamese	10	10
**EAS**	CHS	Southern Han Chinese	10	10
**EAS**	JPT	Japanese	10	10
**EAS**	CDX	Dai Chinese	10	10
**EAS**	CHB	Han Chinese	11	11

* Centre d'Étude du Polymorphisme Humain – Utah.

Table S3. Continental, super-population level summary of ntRoot GAI results on representative 1kGP whole-genome sequencing datasets (n=266, WGS validation dataset), compared to their Assigned Label Counts.

Super-Population Code	Super-Population Name	Assigned Label Count	GAI Correct Prediction Count
**AFR**	African	73	73
**AMR**	Admixed American	41	41
**EAS**	East Asian	51	51
**EUR**	European	51	51
**SAS**	South Asian	50	50
**TOTAL**	-	266	266 (100.0%)

Table S4. ntRoot super-population level GAI summary on the 1kGP WGS validation dataset. Reported metrics include accuracy, precision, recall, and F1 score. The super-populations are EAS: East Asian, SAS: South Asian, AMR: Admixed American, AFR: African, EUR: European.

Accession	Alternate ID	Population Label	Super-Population Label	GAI	SNV Count	Non-zero AF SNV Count	GAI score
**ERR3242189**	HG01882	ACB	AFR	AFR	3,186,519	3,167,755	0.4414
**ERR3242193**	HG02014	ACB	AFR	AFR	3,137,786	3,111,573	0.4421
**ERR3242218**	HG01914	ACB	AFR	AFR	3,172,303	3,143,270	0.4403
**ERR3242297**	HG01988	ACB	AFR	AFR	2,988,030	2,930,917	0.4388
**ERR3242299**	HG02051	ACB	AFR	AFR	3,206,909	3,189,368	0.4415
**ERR3242306**	HG02143	ACB	AFR	AFR	3,168,705	3,136,300	0.4389
**ERR3242308**	HG02307	ACB	AFR	AFR	3,170,854	3,150,587	0.4423
**ERR3242318**	HG02330	ACB	AFR	AFR	3,127,777	3,102,525	0.4428
**ERR3242321**	HG02419	ACB	AFR	AFR	3,124,724	3,103,323	0.4442
**ERR3242322**	HG02420	ACB	AFR	AFR	3,016,590	2,963,682	0.4395
**ERR3242326**	HG02442	ACB	AFR	AFR	3,160,348	3,142,986	0.4434
**ERR3242740**	HG02541	ACB	AFR	AFR	3,128,220	3,094,466	0.4407
**ERR3239918**	NA19711	ASW	AFR	AFR	3,149,808	3,124,561	0.4416
**ERR3239961**	NA19834	ASW	AFR	AFR	3,137,894	3,100,899	0.4392
**ERR3239963**	NA19900	ASW	AFR	AFR	3,129,227	3,098,140	0.4408
**ERR3239965**	NA19904	ASW	AFR	AFR	3,159,895	3,129,145	0.4394
**ERR3239973**	NA19982	ASW	AFR	AFR	3,133,911	3,107,279	0.4415
**ERR3239974**	NA20126	ASW	AFR	AFR	3,060,072	3,018,048	0.4404
**ERR3239982**	NA20291	ASW	AFR	AFR	3,141,807	3,124,530	0.4442
**ERR3239990**	NA20340	ASW	AFR	AFR	3,146,409	3,117,144	0.4410
**ERR3239993**	NA20348	ASW	AFR	AFR	3,158,165	3,127,319	0.4400
**ERR3240095**	NA19922	ASW	AFR	AFR	3,081,920	3,040,542	0.4396
**ERR3242481**	HG02923	ESN	AFR	AFR	3,158,237	3,143,080	0.4445
**ERR3242487**	HG03100	ESN	AFR	AFR	3,197,023	3,181,912	0.4428
**ERR3242587**	HG03268	ESN	AFR	AFR	3,167,022	3,151,949	0.4441
**ERR3242589**	HG03271	ESN	AFR	AFR	3,185,636	3,170,682	0.4432
**ERR3242612**	HG03160	ESN	AFR	AFR	3,211,873	3,196,857	0.4417
**ERR3242623**	HG03343	ESN	AFR	AFR	3,231,466	3,216,418	0.4417
**ERR3242982**	HG03127	ESN	AFR	AFR	3,202,430	3,187,118	0.4427
**ERR3242986**	HG03175	ESN	AFR	AFR	3,212,686	3,197,683	0.4416
**ERR3242991**	HG03265	ESN	AFR	AFR	3,132,528	3,117,465	0.4460
**ERR3242993**	HG03298	ESN	AFR	AFR	3,211,047	3,195,779	0.4419
**ERR3242357**	HG02461	GWD	AFR	AFR	3,172,040	3,155,906	0.4448
**ERR3242365**	HG02573	GWD	AFR	AFR	3,192,014	3,174,718	0.4425
**ERR3242373**	HG02594	GWD	AFR	AFR	3,156,410	3,140,070	0.4449
**ERR3242379**	HG02623	GWD	AFR	AFR	3,155,106	3,139,185	0.4447
**ERR3242385**	HG02645	GWD	AFR	AFR	3,169,900	3,153,671	0.4438
**ERR3242391**	HG02678	GWD	AFR	AFR	3,118,185	3,101,143	0.4460
**ERR3242395**	HG02721	GWD	AFR	AFR	3,159,119	3,142,210	0.4441
**ERR3242423**	HG02768	GWD	AFR	AFR	3,196,630	3,180,432	0.4427
**ERR3242429**	HG02804	GWD	AFR	AFR	3,206,479	3,190,618	0.4419
**ERR3242457**	HG02816	GWD	AFR	AFR	3,190,090	3,173,346	0.4438
**ERR3239689**	NA19026	LWK	AFR	AFR	3,110,637	3,092,710	0.4452
**ERR3239698**	NA19041	LWK	AFR	AFR	3,174,494	3,156,605	0.4435
**ERR3239706**	NA19309	LWK	AFR	AFR	3,202,156	3,184,246	0.4421
**ERR3239708**	NA19312	LWK	AFR	AFR	3,163,420	3,145,640	0.4431
**ERR3239714**	NA19319	LWK	AFR	AFR	3,192,714	3,174,618	0.4424
**ERR3239726**	NA19347	LWK	AFR	AFR	3,163,453	3,145,926	0.4435
**ERR3239727**	NA19350	LWK	AFR	AFR	3,130,396	3,112,541	0.4443
**ERR3239738**	NA19380	LWK	AFR	AFR	3,123,986	3,105,442	0.4452
**ERR3239744**	NA19393	LWK	AFR	AFR	3,185,803	3,167,871	0.4418
**ERR3239752**	NA19428	LWK	AFR	AFR	3,155,303	3,137,504	0.4435
**ERR3239768**	NA19451	LWK	AFR	AFR	3,177,078	3,159,053	0.4425
**ERR3242471**	HG03084	MSL	AFR	AFR	3,199,546	3,183,933	0.4404
**ERR3242476**	HG03451	MSL	AFR	AFR	3,190,982	3,175,496	0.4420
**ERR3242551**	HG03439	MSL	AFR	AFR	3,219,858	3,204,118	0.4396
**ERR3242559**	HG03066	MSL	AFR	AFR	3,225,280	3,209,755	0.4396
**ERR3242568**	HG03484	MSL	AFR	AFR	3,243,027	3,227,586	0.4393
**ERR3242570**	HG03547	MSL	AFR	AFR	3,205,453	3,189,830	0.4408
**ERR3242578**	HG03565	MSL	AFR	AFR	3,228,611	3,212,922	0.4395

**ERR3242952**	HG03060	MSL	AFR	AFR	3,212,658	3,197,009	0.4403
**ERR3242966**	HG03382	MSL	AFR	AFR	3,166,825	3,150,852	0.4417
**ERR3242968**	HG03432	MSL	AFR	AFR	3,227,029	3,211,516	0.4399
**ERR3239341**	NA18504	YRI	AFR	AFR	3,220,233	3,205,057	0.4418
**ERR3239349**	NA18519	YRI	AFR	AFR	3,210,002	3,194,891	0.4428
**ERR3239387**	NA18871	YRI	AFR	AFR	3,239,814	3,224,800	0.4413
**ERR3239429**	NA19119	YRI	AFR	AFR	3,222,033	3,207,023	0.4423
**ERR3239551**	NA18910	YRI	AFR	AFR	3,202,713	3,187,530	0.4429
**ERR3239556**	NA18934	YRI	AFR	AFR	3,226,763	3,211,770	0.4419
**ERR3239619**	NA19096	YRI	AFR	AFR	3,203,540	3,188,547	0.4422
**ERR3239622**	NA19117	YRI	AFR	AFR	3,154,229	3,139,147	0.4441
**ERR3239626**	NA19146	YRI	AFR	AFR	3,187,343	3,172,239	0.4430
**ERR3239629**	NA19184	YRI	AFR	AFR	3,217,574	3,202,556	0.4426
**ERR3241834**	HG01136	CLM	AMR	AMR	2,752,323	2,701,315	0.4759
**ERR3241862**	HG01250	CLM	AMR	AMR	2,749,878	2,700,209	0.4783
**ERR3241875**	HG01344	CLM	AMR	AMR	2,681,692	2,641,536	0.4895
**ERR3241878**	HG01356	CLM	AMR	AMR	2,716,041	2,672,917	0.4816
**ERR3241888**	HG01353	CLM	AMR	AMR	2,723,381	2,683,396	0.4834
**ERR3241894**	HG01374	CLM	AMR	AMR	2,682,698	2,646,860	0.4892
**ERR3241898**	HG01437	CLM	AMR	AMR	2,662,534	2,625,832	0.4863
**ERR3241904**	HG01461	CLM	AMR	AMR	2,781,384	2,709,874	0.4648
**ERR3242787**	HG01130	CLM	AMR	AMR	2,707,307	2,661,499	0.4807
**ERR3242791**	HG01280	CLM	AMR	AMR	2,689,021	2,652,649	0.4884
**ERR3239897**	NA19652	MXL	AMR	AMR	2,684,554	2,641,504	0.4852
**ERR3239902**	NA19661	MXL	AMR	AMR	2,677,412	2,635,126	0.4885
**ERR3239904**	NA19664	MXL	AMR	AMR	2,666,127	2,622,038	0.4884
**ERR3239911**	NA19682	MXL	AMR	AMR	2,653,571	2,616,722	0.4934
**ERR3239928**	NA19726	MXL	AMR	AMR	2,658,275	2,623,563	0.4974
**ERR3239940**	NA19759	MXL	AMR	AMR	2,618,380	2,582,644	0.4998
**ERR3239946**	NA19774	MXL	AMR	AMR	2,678,609	2,634,280	0.4873
**ERR3239950**	NA19780	MXL	AMR	AMR	2,661,645	2,621,548	0.4883
**ERR3239956**	NA19789	MXL	AMR	AMR	2,699,620	2,651,139	0.4829
**ERR3240091**	NA19741	MXL	AMR	AMR	2,598,423	2,566,358	0.5047
**ERR3240094**	NA19792	MXL	AMR	AMR	2,706,649	2,661,706	0.4837
**ERR3241988**	HG01577	PEL	AMR	AMR	2,684,977	2,639,000	0.4869
**ERR3241996**	HG01923	PEL	AMR	AMR	2,546,247	2,517,362	0.5129
**ERR3241998**	HG01926	PEL	AMR	AMR	2,540,590	2,512,437	0.5154
**ERR3242004**	HG01938	PEL	AMR	AMR	2,575,381	2,547,140	0.5125
**ERR3242010**	HG01947	PEL	AMR	AMR	2,633,025	2,594,955	0.4970
**ERR3242018**	HG01970	PEL	AMR	AMR	2,533,693	2,502,472	0.5081
**ERR3242024**	HG01979	PEL	AMR	AMR	2,628,838	2,596,064	0.5021
**ERR3242028**	HG02008	PEL	AMR	AMR	2,640,034	2,608,356	0.5048
**ERR3242241**	HG02271	PEL	AMR	AMR	2,569,248	2,537,914	0.5113
**ERR3242811**	HG01961	PEL	AMR	AMR	2,533,822	2,506,141	0.5160
**ERR3241726**	HG00637	PUR	AMR	AMR	2,759,816	2,699,019	0.4655
**ERR3241728**	HG00640	PUR	AMR	AMR	2,668,638	2,624,708	0.4794
**ERR3241763**	HG01054	PUR	AMR	AMR	2,664,162	2,623,429	0.4811
**ERR3241811**	HG01072	PUR	AMR	AMR	2,718,191	2,671,825	0.4755
**ERR3241816**	HG01085	PUR	AMR	AMR	2,722,139	2,665,212	0.4698
**ERR3241822**	HG01104	PUR	AMR	AMR	2,694,699	2,649,246	0.4775
**ERR3241844**	HG01173	PUR	AMR	AMR	2,676,271	2,631,773	0.4780
**ERR3241854**	HG01197	PUR	AMR	AMR	2,685,646	2,643,155	0.4803
**ERR3242774**	HG01161	PUR	AMR	AMR	2,776,409	2,708,994	0.4611
**ERR3242776**	HG01164	PUR	AMR	AMR	2,700,734	2,652,064	0.4753
**ERR3242164**	HG01816	CDX	EAS	EAS	2,575,551	2,532,627	0.5351
**ERR3242214**	HG02373	CDX	EAS	EAS	2,668,456	2,625,211	0.5298
**ERR3242216**	HG02396	CDX	EAS	EAS	2,631,855	2,588,417	0.5328
**ERR3242217**	HG02397	CDX	EAS	EAS	2,613,135	2,569,460	0.5329
**ERR3242262**	HG02353	CDX	EAS	EAS	2,627,644	2,583,837	0.5315
**ERR3242265**	HG02360	CDX	EAS	EAS	2,608,205	2,564,862	0.5335
**ERR3242275**	HG02384	CDX	EAS	EAS	2,604,758	2,560,807	0.5337
**ERR3242279**	HG02390	CDX	EAS	EAS	2,590,254	2,546,518	0.5342
**ERR3242285**	HG02401	CDX	EAS	EAS	2,623,556	2,579,880	0.5316
**ERR3242289**	HG02409	CDX	EAS	EAS	2,637,355	2,593,908	0.5327
**ERR3239362**	NA18558	CHB	EAS	EAS	2,604,389	2,559,175	0.5323
**ERR3239363**	NA18561	CHB	EAS	EAS	2,612,695	2,565,500	0.5314
**ERR3239370**	NA18572	CHB	EAS	EAS	2,679,439	2,635,041	0.5302
**ERR3239378**	NA18605	CHB	EAS	EAS	2,642,200	2,596,043	0.5311

**ERR3239379**	NA18608	CHB	EAS	EAS	2,621,978	2,575,231	0.5311
**ERR3239496**	NA18544	CHB	EAS	EAS	2,631,366	2,585,755	0.5312
**ERR3239501**	NA18559	CHB	EAS	EAS	2,659,175	2,614,869	0.5315
**ERR3239510**	NA18612	CHB	EAS	EAS	2,676,866	2,632,454	0.5287
**ERR3239511**	NA18613	CHB	EAS	EAS	2,632,870	2,587,786	0.5316
**ERR3239534**	NA18639	CHB	EAS	EAS	2,608,848	2,566,038	0.5344
**ERR3239660**	NA18629	CHB	EAS	EAS	2,662,410	2,619,065	0.5311
**ERR3240183**	HG00442	CHS	EAS	EAS	2,643,529	2,601,142	0.5326
**ERR3241665**	HG00403	CHS	EAS	EAS	2,637,645	2,595,071	0.5316
**ERR3241673**	HG00436	CHS	EAS	EAS	2,618,115	2,575,429	0.5339
**ERR3241675**	HG00457	CHS	EAS	EAS	2,651,857	2,609,443	0.5321
**ERR3241681**	HG00478	CHS	EAS	EAS	2,617,084	2,573,850	0.5347
**ERR3241685**	HG00524	CHS	EAS	EAS	2,637,335	2,594,462	0.5321
**ERR3241697**	HG00556	CHS	EAS	EAS	2,643,691	2,600,925	0.5328
**ERR3241707**	HG00589	CHS	EAS	EAS	2,633,437	2,590,648	0.5336
**ERR3241715**	HG00610	CHS	EAS	EAS	2,646,040	2,602,961	0.5317
**ERR3241723**	HG00628	CHS	EAS	EAS	2,655,588	2,612,379	0.5309
**ERR3239401**	NA18952	JPT	EAS	EAS	2,639,995	2,597,141	0.5314
**ERR3239564**	NA18966	JPT	EAS	EAS	2,662,444	2,619,348	0.5287
**ERR3239574**	NA18995	JPT	EAS	EAS	2,644,569	2,601,671	0.5312
**ERR3239578**	NA19000	JPT	EAS	EAS	2,658,942	2,616,126	0.5306
**ERR3239583**	NA19009	JPT	EAS	EAS	2,663,461	2,620,483	0.5298
**ERR3239590**	NA19058	JPT	EAS	EAS	2,624,383	2,581,149	0.5315
**ERR3239673**	NA18982	JPT	EAS	EAS	2,609,073	2,566,064	0.5318
**ERR3239675**	NA18984	JPT	EAS	EAS	2,613,772	2,571,434	0.5319
**ERR3239678**	NA18988	JPT	EAS	EAS	2,628,364	2,585,633	0.5314
**ERR3239681**	NA19006	JPT	EAS	EAS	2,623,314	2,580,367	0.5321
**ERR3242036**	HG02017	KHV	EAS	EAS	2,660,111	2,611,411	0.5281
**ERR3242038**	HG02020	KHV	EAS	EAS	2,619,942	2,573,659	0.5306
**ERR3242043**	HG02029	KHV	EAS	EAS	2,640,424	2,597,479	0.5320
**ERR3242047**	HG02070	KHV	EAS	EAS	2,617,710	2,572,571	0.5324
**ERR3242049**	HG02073	KHV	EAS	EAS	2,631,017	2,586,134	0.5322
**ERR3242069**	HG01842	KHV	EAS	EAS	2,645,543	2,599,017	0.5301
**ERR3242079**	HG01852	KHV	EAS	EAS	2,660,322	2,613,546	0.5296
**ERR3242092**	HG01867	KHV	EAS	EAS	2,647,088	2,599,620	0.5293
**ERR3242210**	HG02122	KHV	EAS	EAS	2,646,683	2,601,392	0.5297
**ERR3242343**	HG02047	KHV	EAS	EAS	2,651,613	2,605,275	0.5298
**ERR3239281**	NA07051	CEU	EUR	EUR	2,610,640	2,570,725	0.4967
**ERR3239285**	NA10851	CEU	EUR	EUR	2,629,778	2,590,367	0.4958
**ERR3239286**	NA11829	CEU	EUR	EUR	2,656,801	2,616,970	0.4951
**ERR3239288**	NA11831	CEU	EUR	EUR	2,636,146	2,595,221	0.4950
**ERR3239294**	NA11919	CEU	EUR	EUR	2,625,154	2,585,104	0.4961
**ERR3239302**	NA12005	CEU	EUR	EUR	2,587,088	2,547,189	0.4985
**ERR3239309**	NA12155	CEU	EUR	EUR	2,629,238	2,588,866	0.4961
**ERR3239334**	NA12878	CEU	EUR	EUR	2,630,986	2,590,319	0.4951
**ERR3239462**	NA11893	CEU	EUR	EUR	2,628,750	2,589,168	0.4973
**ERR3239473**	NA12342	CEU	EUR	EUR	2,644,497	2,604,098	0.4961
**ERR3239643**	NA11932	CEU	EUR	EUR	2,636,597	2,595,220	0.4950
**ERR3240170**	HG00183	FIN	EUR	EUR	2,634,112	2,592,974	0.4944
**ERR3240176**	HG00190	FIN	EUR	EUR	2,640,560	2,599,453	0.4944
**ERR3240231**	HG00308	FIN	EUR	EUR	2,602,565	2,559,963	0.4953
**ERR3240255**	HG00351	FIN	EUR	EUR	2,657,287	2,615,144	0.4928
**ERR3240260**	HG00358	FIN	EUR	EUR	2,655,974	2,614,280	0.4921
**ERR3240266**	HG00366	FIN	EUR	EUR	2,649,661	2,607,979	0.4937
**ERR3241786**	HG00277	FIN	EUR	EUR	2,622,037	2,581,315	0.4957
**ERR3241793**	HG00310	FIN	EUR	EUR	2,624,698	2,584,007	0.4952
**ERR3241795**	HG00325	FIN	EUR	EUR	2,641,685	2,600,695	0.4945
**ERR3241802**	HG00341	FIN	EUR	EUR	2,630,414	2,583,433	0.4923
**ERR3240123**	HG00107	GBR	EUR	EUR	2,616,699	2,577,380	0.4974
**ERR3240130**	HG00114	GBR	EUR	EUR	2,627,487	2,586,688	0.4957
**ERR3240142**	HG00136	GBR	EUR	EUR	2,623,098	2,582,788	0.4963
**ERR3240144**	HG00138	GBR	EUR	EUR	2,650,144	2,609,268	0.4941
**ERR3240147**	HG00141	GBR	EUR	EUR	2,615,804	2,575,567	0.4964
**ERR3240150**	HG00145	GBR	EUR	EUR	2,605,787	2,564,751	0.4971
**ERR3240152**	HG00148	GBR	EUR	EUR	2,608,382	2,568,049	0.4966
**ERR3240156**	HG00155	GBR	EUR	EUR	2,603,938	2,563,680	0.4978
**ERR3240157**	HG00157	GBR	EUR	EUR	2,662,884	2,621,612	0.4947
**ERR3240199**	HG00126	GBR	EUR	EUR	2,642,676	2,602,318	0.4951

**ERR3241920**	HG01506	IBS	EUR	EUR	2,625,902	2,572,914	0.4914
**ERR3241930**	HG01521	IBS	EUR	EUR	2,603,334	2,561,611	0.4977
**ERR3241938**	HG01536	IBS	EUR	EUR	2,647,249	2,597,681	0.4905
**ERR3241947**	HG01608	IBS	EUR	EUR	2,619,911	2,567,829	0.4914
**ERR3241952**	HG01617	IBS	EUR	EUR	2,628,237	2,576,407	0.4910
**ERR3241961**	HG01630	IBS	EUR	EUR	2,641,893	2,576,645	0.4841
**ERR3241962**	HG01631	IBS	EUR	EUR	2,605,974	2,561,397	0.4955
**ERR3241967**	HG01672	IBS	EUR	EUR	2,625,316	2,577,958	0.4925
**ERR3241971**	HG01678	IBS	EUR	EUR	2,606,650	2,559,642	0.4945
**ERR3241978**	HG01700	IBS	EUR	EUR	2,593,540	2,551,064	0.4968
**ERR3239795**	NA20512	TSI	EUR	EUR	2,645,142	2,600,933	0.4934
**ERR3239799**	NA20516	TSI	EUR	EUR	2,636,281	2,592,586	0.4938
**ERR3239806**	NA20524	TSI	EUR	EUR	2,645,857	2,602,317	0.4936
**ERR3239809**	NA20528	TSI	EUR	EUR	2,603,839	2,557,156	0.4942
**ERR3239813**	NA20532	TSI	EUR	EUR	2,600,291	2,555,513	0.4951
**ERR3239824**	NA20544	TSI	EUR	EUR	2,635,784	2,591,811	0.4930
**ERR3239835**	NA20755	TSI	EUR	EUR	2,653,922	2,609,649	0.4919
**ERR3239843**	NA20763	TSI	EUR	EUR	2,662,212	2,616,493	0.4924
**ERR3239856**	NA20778	TSI	EUR	EUR	2,649,875	2,605,475	0.4929
**ERR3239882**	NA20814	TSI	EUR	EUR	2,653,039	2,607,218	0.4914
**ERR3242653**	HG03824	BEB	SAS	SAS	2,652,207	2,607,521	0.4928
**ERR3242660**	HG03914	BEB	SAS	SAS	2,660,181	2,616,934	0.4929
**ERR3242840**	HG03809	BEB	SAS	SAS	2,704,732	2,661,438	0.4918
**ERR3242842**	HG03009	BEB	SAS	SAS	2,672,611	2,629,858	0.4928
**ERR3242845**	HG03902	BEB	SAS	SAS	2,728,678	2,684,967	0.4881
**ERR3242854**	HG03585	BEB	SAS	SAS	2,696,732	2,651,785	0.4904
**ERR3242860**	HG03593	BEB	SAS	SAS	2,687,971	2,645,036	0.4918
**ERR3243067**	HG03821	BEB	SAS	SAS	2,679,757	2,637,002	0.4917
**ERR3243070**	HG03600	BEB	SAS	SAS	2,658,259	2,620,724	0.4957
**ERR3243071**	HG03603	BEB	SAS	SAS	2,709,493	2,666,785	0.4910
**ERR3240003**	NA20852	GIH	SAS	SAS	2,645,171	2,605,783	0.4943
**ERR3240013**	NA20870	GIH	SAS	SAS	2,661,167	2,617,930	0.4905
**ERR3240027**	NA20889	GIH	SAS	SAS	2,692,850	2,655,284	0.4931
**ERR3240028**	NA20890	GIH	SAS	SAS	2,662,659	2,621,135	0.4916
**ERR3240034**	NA20897	GIH	SAS	SAS	2,671,429	2,633,128	0.4945
**ERR3240040**	NA20904	GIH	SAS	SAS	2,708,734	2,666,694	0.4891
**ERR3240048**	NA21090	GIH	SAS	SAS	2,681,326	2,644,474	0.4924
**ERR3240059**	NA21104	GIH	SAS	SAS	2,663,310	2,625,738	0.4939
**ERR3240103**	NA20864	GIH	SAS	SAS	2,704,737	2,663,714	0.4903
**ERR3240109**	NA21095	GIH	SAS	SAS	2,605,740	2,567,500	0.4968
**ERR3242711**	HG03775	ITU	SAS	SAS	2,678,841	2,641,804	0.4933
**ERR3242720**	HG03869	ITU	SAS	SAS	2,697,305	2,659,594	0.4920
**ERR3242912**	HG03718	ITU	SAS	SAS	2,642,393	2,606,312	0.4956
**ERR3242917**	HG03785	ITU	SAS	SAS	2,657,891	2,622,265	0.4951
**ERR3242926**	HG03867	ITU	SAS	SAS	2,650,886	2,613,634	0.4949
**ERR3242930**	HG03720	ITU	SAS	SAS	2,681,784	2,644,343	0.4930
**ERR3242935**	HG04015	ITU	SAS	SAS	2,676,290	2,639,040	0.4936
**ERR3243115**	HG03969	ITU	SAS	SAS	2,701,366	2,664,008	0.4929
**ERR3243145**	HG03875	ITU	SAS	SAS	2,685,899	2,646,326	0.4909
**ERR3243149**	HG03871	ITU	SAS	SAS	2,682,697	2,645,506	0.4933
**ERR3242415**	HG02733	PJL	SAS	SAS	2,664,741	2,623,744	0.4918
**ERR3242501**	HG02724	PJL	SAS	SAS	2,690,821	2,651,704	0.4924
**ERR3242505**	HG02783	PJL	SAS	SAS	2,686,933	2,648,537	0.4926
**ERR3242636**	HG03015	PJL	SAS	SAS	2,681,531	2,643,282	0.4928
**ERR3243009**	HG02493	PJL	SAS	SAS	2,686,277	2,642,281	0.4897
**ERR3243013**	HG02651	PJL	SAS	SAS	2,676,535	2,635,209	0.4915
**ERR3243015**	HG02681	PJL	SAS	SAS	2,662,687	2,620,964	0.4913
**ERR3243023**	HG02774	PJL	SAS	SAS	2,653,644	2,613,378	0.4926
**ERR3243029**	HG03624	PJL	SAS	SAS	2,659,315	2,616,840	0.4910
**ERR3243047**	HG01589	PJL	SAS	SAS	2,645,592	2,603,620	0.4923
**ERR3242668**	HG03679	STU	SAS	SAS	2,668,802	2,630,742	0.4933
**ERR3242675**	HG03693	STU	SAS	SAS	2,701,970	2,664,741	0.4930
**ERR3242863**	HG03755	STU	SAS	SAS	2,706,007	2,667,816	0.4910
**ERR3242864**	HG03850	STU	SAS	SAS	2,681,238	2,643,482	0.4932
**ERR3242874**	HG03685	STU	SAS	SAS	2,662,519	2,623,703	0.4941
**ERR3242880**	HG03694	STU	SAS	SAS	2,686,169	2,640,580	0.4905
**ERR3242885**	HG03711	STU	SAS	SAS	2,656,401	2,618,904	0.4942
**ERR3242889**	HG03744	STU	SAS	SAS	2,699,962	2,662,049	0.4925

**ERR3242895**	HG03837	STU	SAS	SAS	2,604,773	2,568,134	0.4968
**ERR3242901**	HG03890	STU	SAS	SAS	2,717,308	2,680,085	0.4910
			**Accuracy**	1.000			
			**Precision**	1.000			
			**Recall**	1.000			
			**F1**	1.000			

Table S5. ntRoot super-population level ancestry fraction estimates (LAI-based) on the 1kGP WGS validation set using 5 Mbp (5Mbt) and 2 Mbp (2Mbt) tiles. Reported metrics include accuracy, precision, recall, and F1 score, in addition to per-sample ancestry fraction estimates. The super-populations are: EAS, East Asian; SAS, South Asian; AMR, Admixed American; AFR, African; EUR, European.

Accession	Alternate ID	Population Code	LAI EAS fraction 5Mbt (%)	LAI SAS fraction 5Mbt (%)	LAI AMR fraction 5Mbt (%)	LAI AFR fraction 5Mbt (%)	LAI EUR fraction 5Mbt (%)	1kGP Assigned Label	GAI Predicted from LAI, 5Mbt	GAI Predicted from LAI, 2Mbt
**ERR3242189**	HG01882	ACB	0.18	0.18	1.58	97.72	0.35	AFR	AFR	AFR
**ERR3242193**	HG02014	ACB	0.18	0.00	4.20	95.45	0.18	AFR	AFR	AFR
**ERR3242218**	HG01914	ACB	0.00	0.35	6.48	91.77	1.40	AFR	AFR	AFR
**ERR3242297**	HG01988	ACB	0.18	1.58	12.61	74.78	10.86	AFR	AFR	AFR
**ERR3242299**	HG02051	ACB	0.18	0.00	0.35	99.47	0.00	AFR	AFR	AFR
**ERR3242306**	HG02143	ACB	0.35	0.35	5.95	92.12	1.23	AFR	AFR	AFR
**ERR3242308**	HG02307	ACB	0.00	0.00	1.93	98.07	0.00	AFR	AFR	AFR
**ERR3242318**	HG02330	ACB	0.00	0.00	5.60	93.87	0.53	AFR	AFR	AFR
**ERR3242321**	HG02419	ACB	0.70	0.00	3.33	95.80	0.18	AFR	AFR	AFR
**ERR3242322**	HG02420	ACB	0.00	1.40	12.78	80.56	5.25	AFR	AFR	AFR
**ERR3242326**	HG02442	ACB	0.00	0.00	0.35	99.65	0.00	AFR	AFR	AFR
**ERR3242740**	HG02541	ACB	0.00	0.35	4.38	92.82	2.45	AFR	AFR	AFR
**ERR3239918**	NA19711	ASW	0.53	0.35	2.80	95.97	0.35	AFR	AFR	AFR
**ERR3239961**	NA19834	ASW	0.18	0.18	10.51	88.44	0.70	AFR	AFR	AFR
**ERR3239963**	NA19900	ASW	0.18	0.18	4.55	92.99	2.10	AFR	AFR	AFR
**ERR3239965**	NA19904	ASW	0.00	0.70	6.83	91.24	1.23	AFR	AFR	AFR
**ERR3239973**	NA19982	ASW	0.18	0.00	2.28	97.02	0.53	AFR	AFR	AFR
**ERR3239974**	NA20126	ASW	0.70	1.23	7.88	85.11	5.08	AFR	AFR	AFR
**ERR3239982**	NA20291	ASW	0.00	0.18	0.53	99.30	0.00	AFR	AFR	AFR
**ERR3239990**	NA20340	ASW	0.18	0.18	5.95	91.94	1.75	AFR	AFR	AFR
**ERR3239993**	NA20348	ASW	0.18	0.70	5.08	92.64	1.40	AFR	AFR	AFR
**ERR3240095**	NA19922	ASW	0.18	1.23	8.41	85.46	4.73	AFR	AFR	AFR
**ERR3242481**	HG02923	ESN	0.00	0.18	0.70	99.12	0.00	AFR	AFR	AFR
**ERR3242487**	HG03100	ESN	0.00	0.18	0.53	99.30	0.00	AFR	AFR	AFR
**ERR3242587**	HG03268	ESN	0.00	0.00	0.18	99.82	0.00	AFR	AFR	AFR
**ERR3242589**	HG03271	ESN	0.00	0.00	0.18	99.82	0.00	AFR	AFR	AFR
**ERR3242612**	HG03160	ESN	0.00	0.00	0.00	100.00	0.00	AFR	AFR	AFR
**ERR3242623**	HG03343	ESN	0.00	0.00	0.35	99.65	0.00	AFR	AFR	AFR
**ERR3242982**	HG03127	ESN	0.00	0.00	0.18	99.82	0.00	AFR	AFR	AFR
**ERR3242986**	HG03175	ESN	0.00	0.00	0.00	100.00	0.00	AFR	AFR	AFR
**ERR3242991**	HG03265	ESN	0.00	0.00	0.53	99.47	0.00	AFR	AFR	AFR
**ERR3242993**	HG03298	ESN	0.00	0.00	0.18	99.82	0.00	AFR	AFR	AFR
**ERR3242357**	HG02461	GWD	0.00	0.00	0.35	99.65	0.00	AFR	AFR	AFR
**ERR3242365**	HG02573	GWD	0.18	0.00	0.18	99.65	0.00	AFR	AFR	AFR
**ERR3242373**	HG02594	GWD	0.00	0.00	1.05	98.95	0.00	AFR	AFR	AFR
**ERR3242379**	HG02623	GWD	0.00	0.00	0.35	99.65	0.00	AFR	AFR	AFR
**ERR3242385**	HG02645	GWD	0.00	0.00	0.53	99.47	0.00	AFR	AFR	AFR
**ERR3242391**	HG02678	GWD	0.00	0.18	1.05	98.77	0.00	AFR	AFR	AFR
**ERR3242395**	HG02721	GWD	0.00	0.00	0.53	99.47	0.00	AFR	AFR	AFR
**ERR3242423**	HG02768	GWD	0.00	0.00	0.70	99.30	0.00	AFR	AFR	AFR
**ERR3242429**	HG02804	GWD	0.00	0.00	0.18	99.82	0.00	AFR	AFR	AFR
**ERR3242457**	HG02816	GWD	0.00	0.00	1.23	98.60	0.18	AFR	AFR	AFR
**ERR3239689**	NA19026	LWK	0.35	0.00	1.23	98.42	0.00	AFR	AFR	AFR
**ERR3239698**	NA19041	LWK	0.00	0.00	0.70	99.30	0.00	AFR	AFR	AFR
**ERR3239706**	NA19309	LWK	0.00	0.00	1.40	98.60	0.00	AFR	AFR	AFR
**ERR3239708**	NA19312	LWK	0.00	0.00	0.88	99.12	0.00	AFR	AFR	AFR
**ERR3239714**	NA19319	LWK	0.18	0.00	0.35	99.47	0.00	AFR	AFR	AFR
**ERR3239726**	NA19347	LWK	0.18	0.00	0.53	99.30	0.00	AFR	AFR	AFR
**ERR3239727**	NA19350	LWK	0.00	0.00	1.40	98.60	0.00	AFR	AFR	AFR
**ERR3239738**	NA19380	LWK	0.00	0.00	1.40	98.60	0.00	AFR	AFR	AFR
**ERR3239744**	NA19393	LWK	0.00	0.00	0.53	99.47	0.00	AFR	AFR	AFR
**ERR3239752**	NA19428	LWK	0.00	0.00	0.88	99.12	0.00	AFR	AFR	AFR
**ERR3239768**	NA19451	LWK	0.00	0.35	0.88	98.77	0.00	AFR	AFR	AFR
**ERR3242471**	HG03084	MSL	0.00	0.00	0.35	99.47	0.18	AFR	AFR	AFR
**ERR3242476**	HG03451	MSL	0.00	0.00	0.53	99.30	0.18	AFR	AFR	AFR
**ERR3242551**	HG03439	MSL	0.00	0.00	0.70	99.30	0.00	AFR	AFR	AFR

**ERR3242559**	HG03066	MSL	0.00	0.00	0.35	99.65	0.00	AFR	AFR	AFR
**ERR3242568**	HG03484	MSL	0.00	0.00	0.18	99.82	0.00	AFR	AFR	AFR
**ERR3242570**	HG03547	MSL	0.00	0.18	0.70	99.12	0.00	AFR	AFR	AFR
**ERR3242578**	HG03565	MSL	0.00	0.00	0.53	99.47	0.00	AFR	AFR	AFR
**ERR3242952**	HG03060	MSL	0.00	0.00	0.53	99.47	0.00	AFR	AFR	AFR
**ERR3242966**	HG03382	MSL	0.00	0.00	0.18	99.82	0.00	AFR	AFR	AFR
**ERR3242968**	HG03432	MSL	0.00	0.18	0.35	99.47	0.00	AFR	AFR	AFR
**ERR3239341**	NA18504	YRI	0.00	0.00	0.18	99.82	0.00	AFR	AFR	AFR
**ERR3239349**	NA18519	YRI	0.00	0.00	0.53	99.47	0.00	AFR	AFR	AFR
**ERR3239387**	NA18871	YRI	0.00	0.00	0.35	99.65	0.00	AFR	AFR	AFR
**ERR3239429**	NA19119	YRI	0.00	0.00	0.70	99.30	0.00	AFR	AFR	AFR
**ERR3239551**	NA18910	YRI	0.18	0.00	0.35	99.47	0.00	AFR	AFR	AFR
**ERR3239556**	NA18934	YRI	0.00	0.00	0.53	99.47	0.00	AFR	AFR	AFR
**ERR3239619**	NA19096	YRI	0.00	0.18	0.53	99.30	0.00	AFR	AFR	AFR
**ERR3239622**	NA19117	YRI	0.00	0.00	0.70	99.30	0.00	AFR	AFR	AFR
**ERR3239626**	NA19146	YRI	0.00	0.00	0.70	99.30	0.00	AFR	AFR	AFR
**ERR3239629**	NA19184	YRI	0.00	0.00	0.70	99.30	0.00	AFR	AFR	AFR
**ERR3241834**	HG01136	CLM	4.90	4.03	51.31	16.99	22.77	AMR	AMR	AMR
**ERR3241862**	HG01250	CLM	7.36	4.20	54.29	14.19	19.96	AMR	AMR	AMR
**ERR3241875**	HG01344	CLM	9.81	3.50	56.39	9.46	20.84	AMR	AMR	AMR
**ERR3241878**	HG01356	CLM	6.48	4.20	53.59	12.61	23.12	AMR	AMR	AMR
**ERR3241888**	HG01353	CLM	3.15	5.08	46.94	9.46	35.38	AMR	AMR	AMR
**ERR3241894**	HG01374	CLM	2.98	7.01	53.06	6.65	30.30	AMR	AMR	AMR
**ERR3241898**	HG01437	CLM	3.68	6.65	39.93	8.23	41.51	AMR	EUR	EUR
**ERR3241904**	HG01461	CLM	10.16	2.98	50.96	26.27	9.63	AMR	AMR	AMR
**ERR3242787**	HG01130	CLM	5.08	4.20	49.74	14.19	26.80	AMR	AMR	AMR
**ERR3242791**	HG01280	CLM	4.38	4.90	56.92	7.71	26.09	AMR	AMR	AMR
**ERR3239897**	NA19652	MXL	5.60	6.30	50.79	11.56	25.74	AMR	AMR	AMR
**ERR3239902**	NA19661	MXL	14.54	3.85	57.79	11.73	12.08	AMR	AMR	AMR
**ERR3239904**	NA19664	MXL	16.29	3.50	59.54	11.03	9.63	AMR	AMR	AMR
**ERR3239911**	NA19682	MXL	14.89	5.43	59.72	5.95	14.01	AMR	AMR	AMR
**ERR3239928**	NA19726	MXL	17.34	2.98	66.02	5.60	8.06	AMR	AMR	AMR
**ERR3239940**	NA19759	MXL	22.07	1.75	66.02	6.13	4.03	AMR	AMR	AMR
**ERR3239946**	NA19774	MXL	10.86	3.33	59.19	12.26	14.36	AMR	AMR	AMR
**ERR3239950**	NA19780	MXL	8.76	3.15	59.72	8.41	19.96	AMR	AMR	AMR
**ERR3239956**	NA19789	MXL	11.56	5.08	58.67	11.91	12.78	AMR	AMR	AMR
**ERR3240091**	NA19741	MXL	28.55	1.93	64.10	3.68	1.75	AMR	AMR	AMR
**ERR3240094**	NA19792	MXL	7.53	6.30	54.82	12.61	18.74	AMR	AMR	AMR
**ERR3241988**	HG01577	PEL	9.46	4.03	60.95	13.31	12.26	AMR	AMR	AMR
**ERR3241996**	HG01923	PEL	29.95	1.05	66.20	2.28	0.53	AMR	AMR	AMR
**ERR3241998**	HG01926	PEL	32.92	0.53	64.97	1.40	0.18	AMR	AMR	AMR
**ERR3242004**	HG01938	PEL	29.77	1.05	66.20	1.93	1.05	AMR	AMR	AMR
**ERR3242010**	HG01947	PEL	15.41	2.28	68.48	7.71	6.13	AMR	AMR	AMR
**ERR3242018**	HG01970	PEL	17.86	3.50	69.53	3.33	5.78	AMR	AMR	AMR
**ERR3242024**	HG01979	PEL	19.61	4.38	66.20	4.73	5.08	AMR	AMR	AMR
**ERR3242028**	HG02008	PEL	26.27	1.58	64.62	4.38	3.15	AMR	AMR	AMR
**ERR3242241**	HG02271	PEL	32.57	1.05	64.80	1.23	0.35	AMR	AMR	AMR
**ERR3242811**	HG01961	PEL	31.17	0.53	67.95	0.35	0.00	AMR	AMR	AMR
**ERR3241726**	HG00637	PUR	2.28	5.43	44.13	23.82	24.34	AMR	AMR	AMR
**ERR3241728**	HG00640	PUR	3.68	8.06	41.33	14.19	32.75	AMR	AMR	EUR
**ERR3241763**	HG01054	PUR	3.50	4.73	47.46	14.36	29.95	AMR	AMR	AMR
**ERR3241811**	HG01072	PUR	4.03	5.95	47.64	14.01	28.37	AMR	AMR	AMR
**ERR3241816**	HG01085	PUR	2.63	5.08	42.73	21.02	28.55	AMR	AMR	AMR
**ERR3241822**	HG01104	PUR	3.15	6.13	44.48	14.01	32.22	AMR	AMR	AMR
**ERR3241844**	HG01173	PUR	3.15	7.71	39.93	13.49	35.73	AMR	AMR	EUR
**ERR3241854**	HG01197	PUR	3.68	5.60	44.31	15.24	31.17	AMR	AMR	AMR
**ERR3242774**	HG01161	PUR	3.68	3.50	45.01	27.67	20.14	AMR	AMR	AMR
**ERR3242776**	HG01164	PUR	2.10	5.78	44.13	17.86	30.12	AMR	AMR	AMR
**ERR3242164**	HG01816	CDX	98.25	0.18	0.70	0.70	0.18	EAS	EAS	EAS
**ERR3242214**	HG02373	CDX	97.72	0.70	0.35	1.23	0.00	EAS	EAS	EAS
**ERR3242216**	HG02396	CDX	97.72	0.53	0.88	0.53	0.35	EAS	EAS	EAS
**ERR3242217**	HG02397	CDX	97.55	0.88	0.70	0.88	0.00	EAS	EAS	EAS
**ERR3242262**	HG02353	CDX	97.02	0.70	0.88	1.23	0.18	EAS	EAS	EAS
**ERR3242265**	HG02360	CDX	97.90	0.35	0.88	0.53	0.35	EAS	EAS	EAS
**ERR3242275**	HG02384	CDX	97.02	0.70	1.05	0.88	0.35	EAS	EAS	EAS
**ERR3242279**	HG02390	CDX	97.37	0.88	0.18	1.40	0.18	EAS	EAS	EAS
**ERR3242285**	HG02401	CDX	97.20	0.70	0.88	1.23	0.00	EAS	EAS	EAS
**ERR3242289**	HG02409	CDX	97.02	1.05	1.23	0.35	0.35	EAS	EAS	EAS

**ERR3239362**	NA18558	CHB	97.02	1.05	0.88	0.88	0.18	EAS	EAS	EAS
**ERR3239363**	NA18561	CHB	95.45	0.88	1.93	1.58	0.18	EAS	EAS	EAS
**ERR3239370**	NA18572	CHB	98.07	0.18	1.05	0.53	0.18	EAS	EAS	EAS
**ERR3239378**	NA18605	CHB	97.55	0.70	0.35	1.05	0.35	EAS	EAS	EAS
**ERR3239379**	NA18608	CHB	96.15	1.23	1.05	1.40	0.18	EAS	EAS	EAS
**ERR3239496**	NA18544	CHB	96.67	1.23	1.05	0.53	0.53	EAS	EAS	EAS
**ERR3239501**	NA18559	CHB	97.55	0.35	0.53	0.88	0.70	EAS	EAS	EAS
**ERR3239510**	NA18612	CHB	97.55	0.70	1.23	0.35	0.18	EAS	EAS	EAS
**ERR3239511**	NA18613	CHB	98.25	0.53	0.70	0.53	0.00	EAS	EAS	EAS
**ERR3239534**	NA18639	CHB	98.42	0.35	0.35	0.88	0.00	EAS	EAS	EAS
**ERR3239660**	NA18629	CHB	97.37	0.70	1.05	0.70	0.18	EAS	EAS	EAS
**ERR3240183**	HG00442	CHS	97.72	0.35	0.70	0.88	0.35	EAS	EAS	EAS
**ERR3241665**	HG00403	CHS	97.37	0.53	0.88	1.05	0.18	EAS	EAS	EAS
**ERR3241673**	HG00436	CHS	97.90	0.88	0.53	0.70	0.00	EAS	EAS	EAS
**ERR3241675**	HG00457	CHS	97.72	0.53	0.53	0.88	0.35	EAS	EAS	EAS
**ERR3241681**	HG00478	CHS	97.20	0.70	0.88	1.23	0.00	EAS	EAS	EAS
**ERR3241685**	HG00524	CHS	97.37	0.53	1.40	0.70	0.00	EAS	EAS	EAS
**ERR3241697**	HG00556	CHS	97.20	0.70	0.53	1.40	0.18	EAS	EAS	EAS
**ERR3241707**	HG00589	CHS	98.07	0.35	0.88	0.70	0.00	EAS	EAS	EAS
**ERR3241715**	HG00610	CHS	98.07	0.70	0.35	0.88	0.00	EAS	EAS	EAS
**ERR3241723**	HG00628	CHS	97.20	1.75	0.70	0.35	0.00	EAS	EAS	EAS
**ERR3239401**	NA18952	JPT	97.37	0.70	0.88	1.05	0.00	EAS	EAS	EAS
**ERR3239564**	NA18966	JPT	97.90	0.88	0.88	0.18	0.18	EAS	EAS	EAS
**ERR3239574**	NA18995	JPT	96.85	0.88	0.88	1.05	0.35	EAS	EAS	EAS
**ERR3239578**	NA19000	JPT	97.55	0.35	1.05	0.88	0.18	EAS	EAS	EAS
**ERR3239583**	NA19009	JPT	97.20	0.53	0.88	1.05	0.35	EAS	EAS	EAS
**ERR3239590**	NA19058	JPT	96.67	0.53	1.40	1.40	0.00	EAS	EAS	EAS
**ERR3239673**	NA18982	JPT	97.37	0.70	0.35	1.23	0.35	EAS	EAS	EAS
**ERR3239675**	NA18984	JPT	97.55	0.35	1.40	0.70	0.00	EAS	EAS	EAS
**ERR3239678**	NA18988	JPT	97.90	0.18	0.88	1.05	0.00	EAS	EAS	EAS
**ERR3239681**	NA19006	JPT	97.72	0.53	1.05	0.70	0.00	EAS	EAS	EAS
**ERR3242036**	HG02017	KHV	97.02	0.88	0.70	1.05	0.35	EAS	EAS	EAS
**ERR3242038**	HG02020	KHV	97.20	1.40	0.70	0.70	0.00	EAS	EAS	EAS
**ERR3242043**	HG02029	KHV	96.67	0.70	0.88	1.58	0.18	EAS	EAS	EAS
**ERR3242047**	HG02070	KHV	97.37	0.88	0.53	0.53	0.70	EAS	EAS	EAS
**ERR3242049**	HG02073	KHV	97.55	0.53	0.70	1.23	0.00	EAS	EAS	EAS
**ERR3242069**	HG01842	KHV	97.37	0.35	1.40	0.88	0.00	EAS	EAS	EAS
**ERR3242079**	HG01852	KHV	97.20	0.53	1.40	0.70	0.18	EAS	EAS	EAS
**ERR3242092**	HG01867	KHV	96.15	0.88	0.70	1.93	0.35	EAS	EAS	EAS
**ERR3242210**	HG02122	KHV	96.32	1.05	1.23	1.40	0.00	EAS	EAS	EAS
**ERR3242343**	HG02047	KHV	97.20	0.53	0.70	1.40	0.18	EAS	EAS	EAS
**ERR3239281**	NA07051	CEU	1.40	11.03	11.38	6.30	69.88	EUR	EUR	EUR
**ERR3239285**	NA10851	CEU	0.88	9.28	11.73	4.73	73.38	EUR	EUR	EUR
**ERR3239286**	NA11829	CEU	1.05	10.16	12.08	5.43	71.28	EUR	EUR	EUR
**ERR3239288**	NA11831	CEU	1.58	10.68	14.89	4.03	68.83	EUR	EUR	EUR
**ERR3239294**	NA11919	CEU	1.05	10.33	13.13	5.78	69.70	EUR	EUR	EUR
**ERR3239302**	NA12005	CEU	1.75	10.51	12.61	4.38	70.75	EUR	EUR	EUR
**ERR3239309**	NA12155	CEU	1.40	9.81	13.31	4.73	70.75	EUR	EUR	EUR
**ERR3239334**	NA12878	CEU	1.05	8.93	14.01	5.95	70.05	EUR	EUR	EUR
**ERR3239462**	NA11893	CEU	1.75	9.46	12.78	5.08	70.93	EUR	EUR	EUR
**ERR3239473**	NA12342	CEU	0.88	10.86	13.31	6.13	68.83	EUR	EUR	EUR
**ERR3239643**	NA11932	CEU	1.05	9.11	14.54	5.95	69.35	EUR	EUR	EUR
**ERR3240170**	HG00183	FIN	3.68	9.11	13.84	6.13	67.25	EUR	EUR	EUR
**ERR3240176**	HG00190	FIN	2.98	10.68	12.08	5.78	68.48	EUR	EUR	EUR
**ERR3240231**	HG00308	FIN	5.60	9.28	12.26	6.83	66.02	EUR	EUR	EUR
**ERR3240255**	HG00351	FIN	4.90	10.33	12.43	5.08	67.25	EUR	EUR	EUR
**ERR3240260**	HG00358	FIN	5.95	8.93	12.08	4.03	69.00	EUR	EUR	EUR
**ERR3240266**	HG00366	FIN	4.73	9.46	12.61	4.90	68.30	EUR	EUR	EUR
**ERR3241786**	HG00277	FIN	3.50	11.91	15.94	5.60	63.05	EUR	EUR	EUR
**ERR3241793**	HG00310	FIN	4.90	9.11	12.26	4.55	69.18	EUR	EUR	EUR
**ERR3241795**	HG00325	FIN	5.43	10.16	12.08	6.13	66.20	EUR	EUR	EUR
**ERR3241802**	HG00341	FIN	2.80	12.61	14.19	6.65	63.75	EUR	EUR	EUR
**ERR3240123**	HG00107	GBR	2.63	8.93	13.31	6.13	69.00	EUR	EUR	EUR
**ERR3240130**	HG00114	GBR	1.40	8.76	14.36	5.60	69.88	EUR	EUR	EUR
**ERR3240142**	HG00136	GBR	1.58	9.28	13.13	5.25	70.75	EUR	EUR	EUR
**ERR3240144**	HG00138	GBR	1.40	8.58	13.31	5.60	71.10	EUR	EUR	EUR
**ERR3240147**	HG00141	GBR	1.75	8.58	13.31	4.38	71.98	EUR	EUR	EUR
**ERR3240150**	HG00145	GBR	1.23	8.58	13.31	7.71	69.18	EUR	EUR	EUR

**ERR3240152**	HG00148	GBR	1.75	7.53	11.38	6.65	72.68	EUR	EUR	EUR
**ERR3240156**	HG00155	GBR	1.40	9.81	14.89	5.78	68.13	EUR	EUR	EUR
**ERR3240157**	HG00157	GBR	1.75	9.98	14.54	5.25	68.48	EUR	EUR	EUR
**ERR3240199**	HG00126	GBR	1.93	8.41	13.13	6.65	69.88	EUR	EUR	EUR
**ERR3241920**	HG01506	IBS	1.75	8.93	14.89	9.81	64.62	EUR	EUR	EUR
**ERR3241930**	HG01521	IBS	1.05	7.71	17.16	5.43	68.65	EUR	EUR	EUR
**ERR3241938**	HG01536	IBS	1.40	8.06	16.99	9.63	63.92	EUR	EUR	EUR
**ERR3241947**	HG01608	IBS	0.88	8.41	17.34	9.28	64.10	EUR	EUR	EUR
**ERR3241952**	HG01617	IBS	1.58	9.28	16.99	8.76	63.40	EUR	EUR	EUR
**ERR3241961**	HG01630	IBS	1.23	7.88	22.77	10.51	57.62	EUR	EUR	EUR
**ERR3241962**	HG01631	IBS	1.40	8.93	15.76	5.43	68.48	EUR	EUR	EUR
**ERR3241967**	HG01672	IBS	1.23	8.58	16.81	8.23	65.15	EUR	EUR	EUR
**ERR3241971**	HG01678	IBS	2.10	8.23	16.11	7.88	65.67	EUR	EUR	EUR
**ERR3241978**	HG01700	IBS	1.40	5.95	18.21	4.90	69.53	EUR	EUR	EUR
**ERR3239795**	NA20512	TSI	1.23	11.38	13.49	7.36	66.55	EUR	EUR	EUR
**ERR3239799**	NA20516	TSI	1.75	10.51	14.71	6.30	66.73	EUR	EUR	EUR
**ERR3239806**	NA20524	TSI	1.75	10.86	14.89	6.48	66.02	EUR	EUR	EUR
**ERR3239809**	NA20528	TSI	1.23	9.81	16.81	6.83	65.32	EUR	EUR	EUR
**ERR3239813**	NA20532	TSI	1.58	10.51	15.41	7.18	65.32	EUR	EUR	EUR
**ERR3239824**	NA20544	TSI	1.75	11.91	16.29	6.65	63.40	EUR	EUR	EUR
**ERR3239835**	NA20755	TSI	0.88	12.61	14.36	6.65	65.50	EUR	EUR	EUR
**ERR3239843**	NA20763	TSI	1.23	11.21	14.54	7.71	65.32	EUR	EUR	EUR
**ERR3239856**	NA20778	TSI	1.40	14.36	13.84	7.53	62.87	EUR	EUR	EUR
**ERR3239882**	NA20814	TSI	2.10	9.46	13.31	9.28	65.85	EUR	EUR	EUR
**ERR3242653**	HG03824	BEB	21.37	65.67	4.03	5.25	3.68	SAS	SAS	SAS
**ERR3242660**	HG03914	BEB	17.51	73.38	2.98	3.68	2.45	SAS	SAS	SAS
**ERR3242840**	HG03809	BEB	17.16	72.50	3.68	3.68	2.98	SAS	SAS	SAS
**ERR3242842**	HG03009	BEB	15.06	72.50	3.50	4.38	4.55	SAS	SAS	SAS
**ERR3242845**	HG03902	BEB	16.29	72.33	3.85	3.68	3.85	SAS	SAS	SAS
**ERR3242854**	HG03585	BEB	17.69	68.65	4.20	5.78	3.68	SAS	SAS	SAS
**ERR3242860**	HG03593	BEB	17.51	72.50	3.50	3.68	2.80	SAS	SAS	SAS
**ERR3243067**	HG03821	BEB	16.11	74.61	2.10	4.38	2.80	SAS	SAS	SAS
**ERR3243070**	HG03600	BEB	8.93	83.19	1.75	4.38	1.75	SAS	SAS	SAS
**ERR3243071**	HG03603	BEB	17.16	73.38	2.98	4.38	2.10	SAS	SAS	SAS
**ERR3240003**	NA20852	GIH	7.18	80.21	3.33	3.85	5.43	SAS	SAS	SAS
**ERR3240013**	NA20870	GIH	4.03	70.40	5.95	5.95	13.66	SAS	SAS	SAS
**ERR3240027**	NA20889	GIH	5.08	86.51	2.28	3.50	2.63	SAS	SAS	SAS
**ERR3240028**	NA20890	GIH	5.25	76.01	3.85	6.48	8.41	SAS	SAS	SAS
**ERR3240034**	NA20897	GIH	9.46	82.31	3.15	4.03	1.05	SAS	SAS	SAS
**ERR3240040**	NA20904	GIH	6.65	71.80	7.01	4.38	10.16	SAS	SAS	SAS
**ERR3240048**	NA21090	GIH	5.25	87.57	1.40	3.33	2.45	SAS	SAS	SAS
**ERR3240059**	NA21104	GIH	7.18	83.54	2.28	4.03	2.98	SAS	SAS	SAS
**ERR3240103**	NA20864	GIH	4.90	77.76	3.50	5.43	8.41	SAS	SAS	SAS
**ERR3240109**	NA21095	GIH	5.25	83.19	3.15	3.85	4.55	SAS	SAS	SAS
**ERR3242711**	HG03775	ITU	7.53	83.01	1.40	5.25	2.80	SAS	SAS	SAS
**ERR3242720**	HG03869	ITU	8.23	81.79	2.28	5.25	2.45	SAS	SAS	SAS
**ERR3242912**	HG03718	ITU	7.18	88.09	1.23	2.63	0.88	SAS	SAS	SAS
**ERR3242917**	HG03785	ITU	8.93	85.11	1.05	3.15	1.75	SAS	SAS	SAS
**ERR3242926**	HG03867	ITU	13.13	78.11	1.58	4.20	2.98	SAS	SAS	SAS
**ERR3242930**	HG03720	ITU	8.58	82.66	2.98	4.38	1.40	SAS	SAS	SAS
**ERR3242935**	HG04015	ITU	7.53	85.46	1.93	3.33	1.75	SAS	SAS	SAS
**ERR3243115**	HG03969	ITU	6.30	83.54	2.45	4.90	2.80	SAS	SAS	SAS
**ERR3243145**	HG03875	ITU	6.13	78.81	3.15	5.25	6.65	SAS	SAS	SAS
**ERR3243149**	HG03871	ITU	6.30	85.64	2.28	3.85	1.93	SAS	SAS	SAS
**ERR3242415**	HG02733	PJL	5.08	76.88	4.03	4.90	9.11	SAS	SAS	SAS
**ERR3242501**	HG02724	PJL	8.58	80.04	3.68	4.38	3.33	SAS	SAS	SAS
**ERR3242505**	HG02783	PJL	8.41	81.44	2.10	4.38	3.68	SAS	SAS	SAS
**ERR3242636**	HG03015	PJL	8.93	78.98	3.15	4.90	4.03	SAS	SAS	SAS
**ERR3243009**	HG02493	PJL	4.73	76.53	3.33	7.36	8.06	SAS	SAS	SAS
**ERR3243013**	HG02651	PJL	5.60	75.48	4.90	4.38	9.63	SAS	SAS	SAS
**ERR3243015**	HG02681	PJL	5.25	71.28	5.95	5.43	12.08	SAS	SAS	SAS
**ERR3243023**	HG02774	PJL	6.30	78.11	4.20	5.08	6.30	SAS	SAS	SAS
**ERR3243029**	HG03624	PJL	2.98	73.03	6.83	5.60	11.56	SAS	SAS	SAS
**ERR3243047**	HG01589	PJL	5.78	73.20	5.25	4.20	11.56	SAS	SAS	SAS
**ERR3242668**	HG03679	STU	6.83	84.41	3.33	3.50	1.93	SAS	SAS	SAS
**ERR3242675**	HG03693	STU	9.11	82.49	2.28	3.50	2.63	SAS	SAS	SAS
**ERR3242863**	HG03755	STU	8.23	82.31	2.45	4.90	2.10	SAS	SAS	SAS
**ERR3242864**	HG03850	STU	8.06	83.19	2.28	4.38	2.10	SAS	SAS	SAS

**ERR3242874**	HG03685	STU	6.83	83.36	2.98	5.25	1.58	SAS	SAS	SAS
**ERR3242880**	HG03694	STU	12.96	68.65	4.73	6.83	6.83	SAS	SAS	SAS
**ERR3242885**	HG03711	STU	7.71	83.19	2.10	4.90	2.10	SAS	SAS	SAS
**ERR3242889**	HG03744	STU	9.63	81.09	1.93	5.78	1.58	SAS	SAS	SAS
**ERR3242895**	HG03837	STU	8.41	81.09	3.33	4.90	2.28	SAS	SAS	SAS
**ERR3242901**	HG03890	STU	8.93	81.44	2.28	4.38	2.98	SAS	SAS	SAS
								**Accuracy**	0.996	0.989
								**Precision**	0.996	0.989
								**Recall**	0.995	0.985
								**F1**	0.996	0.987

Table S6. ntRoot super-population level LAI-based ancestry fraction estimates on the SGDP WGS discovery set. The LAI fraction estimates were generated using 5Mbp tiles. The super-populations are EAS: East Asian, SAS: South Asian, AMR: Admixed American, AFR: African, EUR: European.

Accession	Dataset Fold Coverage	Library	Population	Region	LAI EAS Fraction (%)	LAI SAS Fraction (%)	LAI AMR Fraction (%)	LAI AFR Fraction (%)	LAI EUR Fraction (%)
**ERR1019043^**	41.0	LP6005441-DNA_F01	BantuHerero	Africa	0.00	0.18	0.18	99.65	0.00
**ERR1347714**	45.2	LP6005443-DNA_E02	BantuHerero	Africa	0.00	0.00	0.18	99.82	0.00
**ERR1019044^**	37.3	LP6005441-DNA_B02	BantuKenya	Africa	0.00	0.00	0.53	99.47	0.00
**ERR1347680**	50.7	LP6005443-DNA_A01	BantuKenya	Africa	0.00	0.00	0.53	99.47	0.00
**ERR1347723^**	39.9	LP6005443-DNA_F02	BantuTswana	Africa	0.00	0.00	0.35	99.65	0.00
**ERR1347732**	44.3	LP6005443-DNA_G02	BantuTswana	Africa	0.00	0.00	0.18	99.82	0.00
**ERR1025612^**	44.3	LP6005441-DNA_G02	Biaka	Africa	0.00	0.00	0.70	99.30	0.00
**ERR1419161**	43.7	LP6005441-DNA_H02	Biaka	Africa	0.00	0.00	0.18	99.82	0.00
**ERR1347694^**	46.3	LP6005443-DNA_B09	Dinka	Africa	0.18	0.00	0.35	99.47	0.00
**ERR1395603**	48.4	SS6004480	Dinka	Africa	0.00	0.00	0.70	99.30	0.00
**ERR1395606**	46.7	LP6005443-DNA_H08	Dinka	Africa	0.00	0.00	1.23	98.77	0.00
**ERR1025621^**	35.6	LP6005442-DNA_A10	Esan	Africa	0.00	0.18	0.53	99.30	0.00
**ERR1025622**	41.9	LP6005442-DNA_B10	Esan	Africa	0.00	0.00	0.35	99.65	0.00
**ERR1347670^**	40.8	LP6005442-DNA_G10	Gambian	Africa	0.35	0.35	0.35	98.95	0.00
**ERR1347677**	39.0	LP6005442-DNA_H10	Gambian	Africa	0.00	0.18	0.70	99.12	0.00
**ERR1347737^**	47.2	LP6005443-DNA_G08	Ju hoan North	Africa	0.00	0.00	0.70	99.30	0.00
**ERR1395596**	42.7	SS6004473	Ju hoan North	Africa	0.00	0.00	1.05	98.95	0.00
**ERR1419096**	38.7	LP6005441-DNA_A11	Ju hoan North	Africa	0.00	0.00	1.23	98.77	0.00
**ERR1419108**	34.8	LP6005441-DNA_B11	Ju hoan North	Africa	0.18	0.18	0.88	98.77	0.00
**ERR1395572^**	53.9	LP6005592-DNA_C05	Khomani San	Africa	0.00	0.18	0.18	99.65	0.00
**ERR1395588**	48.7	LP6005677-DNA_D03	Khomani San	Africa	0.00	0.00	1.05	98.77	0.18
**ERR1347655^**	36.3	LP6005442-DNA_E11	Luhya	Africa	0.00	0.00	1.40	98.60	0.00
**ERR1347662**	39.0	LP6005442-DNA_F11	Luhya	Africa	0.00	0.00	1.05	98.95	0.00
**ERR1347660^**	37.7	LP6005442-DNA_F09	Luo	Africa	0.00	0.00	0.35	99.65	0.00
**ERR1395591**	38.9	LP6005677-DNA_G01	Luo	Africa	0.00	0.00	1.40	98.60	0.00
**ERR1025640^**	35.1	LP6005441-DNA_E07	Mandenka	Africa	0.00	0.00	0.53	99.47	0.00
**ERR1395593**	41.4	SS6004470	Mandenka	Africa	0.00	0.00	0.53	99.12	0.35
**ERR1419145**	39.0	LP6005441-DNA_F07	Mandenka	Africa	0.00	0.00	0.35	99.65	0.00
**ERR1347716^**	39.6	LP6005443-DNA_E06	Masai	Africa	0.00	0.18	7.01	92.29	0.53
**ERR1347726**	48.7	LP6005443-DNA_F06	Masai	Africa	0.18	0.35	6.48	92.47	0.53

**ERR1019077^**	38.1	LP6005441-DNA_B08	Mbuti	Africa	0.00	0.00	0.35	99.65	0.00
**ERR1395571**	42.7	LP6005592-DNA_C03	Mbuti	Africa	0.00	0.00	0.18	99.82	0.00
**ERR1395594**	41.5	SS6004471	Mbuti	Africa	0.00	0.00	0.53	99.47	0.00
**ERR1419093**	39.5	LP6005441-DNA_A08	Mbuti	Africa	0.00	0.00	0.53	99.47	0.00
**ERR1347671^**	40.8	LP6005442-DNA_G11	Mende	Africa	0.18	0.00	0.35	99.47	0.00
**ERR1347678**	37.6	LP6005442-DNA_H11	Mende	Africa	0.00	0.18	0.35	99.47	0.00
**ERR1025601^**	38.0	LP6005441-DNA_G08	Mozabite	Africa	0.18	4.03	27.15	41.86	26.80
**ERR1025602**	40.4	LP6005441-DNA_H08	Mozabite	Africa	0.53	3.33	27.67	50.44	18.04
**ERR1395581^**	47.9	LP6005619-DNA_B01	Saharawi	Africa	0.35	7.01	30.65	40.81	21.19
**ERR1395582**	51.3	LP6005619-DNA_C01	Saharawi	Africa	0.35	4.03	26.80	47.81	21.02
**ERR1025600^**	42.0	LP6005442-DNA_D09	Somali	Africa	0.18	1.23	13.49	82.84	2.28
**ERR1395598^**	44.2	SS6004475	Yoruba	Africa	0.00	0.00	0.18	99.82	0.00
**ERR1419171**	34.5	LP6005442-DNA_A02	Yoruba	Africa	0.00	0.00	0.88	99.12	0.00
**ERR1419179**	36.2	LP6005442-DNA_B02	Yoruba	Africa	0.00	0.18	0.35	99.47	0.00
**ERR1395547^**	59.3	LP6005519-DNA_D01	Chane	America	34.85	0.35	63.57	0.70	0.53
**ERR1025635^**	46.6	LP6005441-DNA_G06	Karitiana	America	36.25	0.70	61.47	1.58	0.00
**ERR1025636**	39.1	LP6005441-DNA_H06	Karitiana	America	38.18	0.53	60.07	1.23	0.00
**ERR1395599**	39.6	SS6004476	Karitiana	America	36.43	0.18	61.82	1.05	0.53
**ERR1025642^**	38.0	LP6005441-DNA_G07	Mayan	America	36.60	0.53	60.95	1.75	0.18
**ERR1419165**	45.6	LP6005441-DNA_H07	Mayan	America	35.20	0.53	62.35	1.75	0.18
**ERR1347721^**	47.8	LP6005443-DNA_E11	Mixe	America	39.05	0.35	59.02	1.40	0.18
**ERR1347730**	47.4	LP6005443-DNA_F11	Mixe	America	35.20	0.53	62.00	2.28	0.00
**ERR1395602**	45.6	SS6004479	Mixe	America	38.53	0.18	59.72	1.23	0.35
**ERR1347738^**	45.1	LP6005443-DNA_G11	Mixtec	America	32.40	1.75	61.12	3.68	1.05
**ERR1395607**	47.5	LP6005443-DNA_H11	Mixtec	America	21.54	2.63	65.85	5.43	4.55
**ERR1019067^**	42.5	LP6005441-DNA_B04	Piapoco	America	36.95	0.53	61.12	1.40	0.00
**ERR1419090**	41.4	LP6005441-DNA_A04	Piapoco	America	35.03	0.70	63.75	0.53	0.00
**ERR1025604^**	38.4	LP6005441-DNA_F10	Pima	America	41.86	0.70	56.04	1.23	0.18
**ERR1419139‡**	21.8	LP6005441-DNA_E10	Pima	America	39.58	0.53	58.14	1.75	0.00
**ERR1395558^**	60.9	LP6005519-DNA_G02	Quechua	America	34.85	0.88	63.22	0.70	0.35
**ERR1395589**	45.8	LP6005677-DNA_E01	Quechua	America	33.80	0.53	64.80	0.70	0.18
**ERR1395590**	42.4	LP6005677-DNA_F01	Quechua	America	31.35	1.05	66.02	1.58	0.00
**ERR1019070^**	41.9	LP6005441-DNA_A12	Surui	America	39.40	0.53	58.32	1.75	0.00
**ERR1019071**	36.1	LP6005441-DNA_B12	Surui	America	38.53	0.70	59.02	1.75	0.00
**ERR1347686^**	46.3	LP6005443-DNA_A12	Zapotec	America	38.88	0.70	59.37	1.05	0.00
**ERR1395622**	39.8	LP6005677-DNA_D01	Zapotec	America	32.40	0.53	64.45	2.28	0.35

**ERR1347682^**	47.0	LP6005443-DNA_A03	Aleut	CentralAsiaSiberia	44.13	7.53	33.63	3.33	11.38
**ERR1347740**	38.8	LP6005443-DNA_H02	Aleut	CentralAsiaSiberia	41.68	8.06	34.50	4.03	11.73
**ERR1419200^**	40.8	LP6005442-DNA_F02	Altaian	CentralAsiaSiberia	74.78	8.93	8.23	3.50	4.55
**ERR1347698^**	47.3	LP6005443-DNA_C03	Chukchi	CentralAsiaSiberia	39.23	11.03	29.95	6.13	13.66
**ERR1347706^**	46.1	LP6005443-DNA_D03	Eskimo Chaplin	CentralAsiaSiberia	79.16	1.23	17.16	1.75	0.70
**ERR1347724^**	49.2	LP6005443-DNA_F03	Eskimo Naukan	CentralAsiaSiberia	76.88	2.28	18.04	2.63	0.18
**ERR1347733**	46.8	LP6005443-DNA_G03	Eskimo Naukan	CentralAsiaSiberia	77.76	2.45	16.99	2.28	0.53
**ERR1347689^**	44.0	LP6005443-DNA_B03	Eskimo Sireniki	CentralAsiaSiberia	78.98	1.58	17.34	1.75	0.35
**ERR1347741**	49.4	LP6005443-DNA_H03	Eskimo Sireniki	CentralAsiaSiberia	75.31	3.15	19.26	1.93	0.35
**ERR1347690^**	50.8	LP6005443-DNA_B04	Even	CentralAsiaSiberia	90.37	1.75	4.38	2.98	0.53
**ERR1347699**	40.7	LP6005443-DNA_C04	Even	CentralAsiaSiberia	91.77	2.28	3.33	1.75	0.88
**ERR1395577**	50.3	LP6005592-DNA_F03	Even	CentralAsiaSiberia	75.66	6.30	8.76	3.68	5.60
**ERR1347707^**	48.3	LP6005443-DNA_D04	Itelman	CentralAsiaSiberia	84.76	3.68	8.06	2.63	0.88
**ERR1395583^**	39.6	LP6005677-DNA_A02	Kyrgyz	CentralAsiaSiberia	68.48	11.38	9.98	2.98	7.18
**ERR1395586**	48.9	LP6005677-DNA_B02	Kyrgyz	CentralAsiaSiberia	69.18	11.91	8.41	3.15	7.36
**ERR1347725^**	39.3	LP6005443-DNA_F04	Mansi	CentralAsiaSiberia	47.64	14.54	18.74	5.25	13.84
**ERR1347734**	46.7	LP6005443-DNA_G04	Mansi	CentralAsiaSiberia	38.00	13.84	21.72	4.73	21.72
**ERR1025645^**	45.1	LP6005441-DNA_F08	Mongola	CentralAsiaSiberia	93.35	2.45	2.28	1.75	0.18
**ERR1419137**	39.5	LP6005441-DNA_E08	Mongola	CentralAsiaSiberia	93.52	1.93	2.28	1.40	0.88
**ERR1347708^**	48.5	LP6005443-DNA_D05	Tlingit	CentralAsiaSiberia	34.33	8.06	34.50	3.50	19.61
**ERR1347715**	45.8	LP6005443-DNA_E05	Tlingit	CentralAsiaSiberia	17.34	12.26	36.60	5.60	28.20
**ERR1347657^**	42.1	LP6005442-DNA_E12	Tubalar	CentralAsiaSiberia	60.25	12.78	12.08	4.20	10.68
**ERR1347663**	39.1	LP6005442-DNA_F12	Tubalar	CentralAsiaSiberia	56.04	14.54	13.84	4.90	10.68
**ERR1347672^**	40.8	LP6005442-DNA_G12	Ulchi	CentralAsiaSiberia	93.87	1.75	2.28	1.75	0.35
**ERR1347679**	43.6	LP6005442-DNA_H12	Ulchi	CentralAsiaSiberia	93.70	1.40	2.63	2.10	0.18
**ERR1347658^**	42.1	LP6005442-DNA_F01	Yakut	CentralAsiaSiberia	85.29	5.08	5.25	2.45	1.93
**ERR1347705**	48.2	LP6005443-DNA_D02	Yakut	CentralAsiaSiberia	87.04	4.38	4.38	1.93	2.28
**ERR1347735^**	47.0	LP6005443-DNA_G05	Ami	EastAsia	97.72	1.05	0.70	0.53	0.00
**ERR1419188**	36.9	LP6005442-DNA_C07	Ami	EastAsia	97.37	0.88	0.53	1.05	0.18
**ERR1419198^**	37.3	LP6005442-DNA_E07	Atayal	EastAsia	97.37	0.35	0.70	1.58	0.00
**ERR1395610^**	55.0	LP6005519-DNA_A06	Burmese	EastAsia	87.22	9.46	0.88	1.93	0.53
**ERR1395613**	60.0	LP6005519-DNA_B06	Burmese	EastAsia	93.17	2.80	2.10	1.58	0.35
**ERR1025617^**	43.0	LP6005441-DNA_G03	Cambodian	EastAsia	91.24	4.03	1.58	2.80	0.35
**ERR1419162**	39.1	LP6005441-DNA_H03	Cambodian	EastAsia	89.49	5.78	1.05	2.98	0.70

**ERR1347687^**	40.7	LP6005443-DNA_B01	Dai	EastAsia	97.72	0.53	0.70	0.70	0.35
**ERR1395573**	50.4	LP6005592-DNA_D03	Dai	EastAsia	97.02	0.88	0.88	0.88	0.35
**ERR1395618**	41.3	SS6004467	Dai	EastAsia	97.20	0.88	1.23	0.70	0.00
**ERR1419123**	39.3	LP6005441-DNA_D04	Dai	EastAsia	96.67	1.40	1.05	0.53	0.35
**ERR1425294^**	46.9	LP6005441-DNA_F04	Daur	EastAsia	93.52	2.28	1.75	1.58	0.88
**ERR1019056^**	43.0	LP6005441-DNA_C05	Han	EastAsia	97.90	0.88	0.88	0.18	0.18
**ERR1395592**	39.9	SS6004469	Han	EastAsia	97.20	0.18	0.88	1.40	0.35
**ERR1419124**	70.5	LP6005441-DNA_D05	Han	EastAsia	97.20	1.05	1.40	0.35	0.00
**ERR1025628^**	41.1	LP6005441-DNA_H05	Hezhen	EastAsia	94.57	1.23	2.28	1.40	0.53
**ERR1419153**	43.4	LP6005441-DNA_G05	Hezhen	EastAsia	94.92	1.58	1.58	1.40	0.53
**ERR1019038^**	87.0	LP6005441-DNA_C06	Japanese	EastAsia	98.07	0.70	0.53	0.53	0.18
**ERR1395570**	48.7	LP6005592-DNA_C02	Japanese	EastAsia	97.20	1.05	0.88	0.53	0.35
**ERR1419125**	43.5	LP6005441-DNA_D06	Japanese	EastAsia	98.07	0.35	1.05	0.53	0.00
**ERR1025638^**	37.3	LP6005442-DNA_C11	Kinh	EastAsia	96.15	0.88	1.40	1.23	0.35
**ERR1419196**	38.3	LP6005442-DNA_D11	Kinh	EastAsia	97.72	0.70	0.70	0.70	0.18
**ERR1347700^**	53.8	LP6005443-DNA_C06	Korean	EastAsia	97.20	1.05	0.88	0.88	0.00
**ERR1347709**	44.7	LP6005443-DNA_D06	Korean	EastAsia	96.32	0.70	1.23	1.40	0.35
**ERR1019057^**	38.4	LP6005441-DNA_B07	Lahu	EastAsia	97.55	1.05	0.70	0.70	0.00
**ERR1347713**	59.4	LP6005443-DNA_E01	Lahu	EastAsia	95.80	1.93	0.88	1.40	0.00
**ERR1019060^**	35.4	LP6005441-DNA_C08	Miao	EastAsia	97.37	0.53	1.23	0.88	0.00
**ERR1419127**	37.7	LP6005441-DNA_D08	Miao	EastAsia	97.02	0.88	0.53	1.23	0.35
**ERR1019039^**	41.5	LP6005441-DNA_A09	Naxi	EastAsia	97.20	0.70	0.88	1.05	0.18
**ERR1019079**	43.5	LP6005441-DNA_B09	Naxi	EastAsia	96.32	0.70	1.05	1.58	0.35
**ERR1347719**	42.2	LP6005443-DNA_E09	Naxi	EastAsia	97.37	0.70	0.88	0.70	0.35
**ERR1025647^**	39.8	LP6005441-DNA_F09	Oroqen	EastAsia	93.70	1.75	1.93	1.40	1.23
**ERR1419138**	39.5	LP6005441-DNA_E09	Oroqen	EastAsia	94.92	0.88	1.93	2.10	0.18
**ERR1347722^**	49.1	LP6005443-DNA_F01	She	EastAsia	96.67	0.35	1.05	1.58	0.35
**ERR1347731**	49.7	LP6005443-DNA_G01	She	EastAsia	97.72	0.53	0.53	1.05	0.18
**ERR1347684^**	51.9	LP6005443-DNA_A07	Thai	EastAsia	87.39	7.71	1.75	2.10	1.05
**ERR1347692**	46.5	LP6005443-DNA_B07	Thai	EastAsia	95.62	2.10	1.23	1.05	0.00
**ERR1347739^**	50.8	LP6005443-DNA_H01	Tu	EastAsia	90.37	3.85	1.58	2.80	1.40
**ERR1419131**	39.2	LP6005441-DNA_D12	Tu	EastAsia	93.87	2.10	1.75	1.75	0.53
**ERR1025652^**	40.9	LP6005441-DNA_F12	Tujia	EastAsia	97.20	0.70	1.23	0.70	0.18
**ERR1347681**	50.2	LP6005443-DNA_A02	Tujia	EastAsia	97.90	0.35	0.70	0.70	0.35
**ERR1025655^**	40.0	LP6005442-DNA_B01	Uygur	EastAsia	54.12	18.21	10.86	4.03	12.78

**ERR1347688**	51.1	LP6005443-DNA_B02	Uygur	EastAsia	47.46	23.64	9.63	5.08	14.19
**ERR1025656^**	38.0	LP6005442-DNA_D01	Xibo	EastAsia	92.82	2.98	1.58	1.93	0.70
**ERR1347697**	52.6	LP6005443-DNA_C02	Xibo	EastAsia	94.75	1.23	2.28	1.05	0.70
**ERR1347664^**	44.2	LP6005442-DNA_G01	Yi	EastAsia	96.85	0.53	1.58	1.05	0.00
**ERR1347673**	42.1	LP6005442-DNA_H01	Yi	EastAsia	95.97	0.70	1.05	1.58	0.70
**ERR1395600^**	44.4	SS6004477	Australian	Oceania	55.52	26.97	4.73	11.38	1.40
**ERR1395601**	46.2	SS6004478	Australian	Oceania	54.12	27.15	4.38	12.78	1.58
**ERR1019048^**	42.2	LP6005441-DNA_B03	Bougainville	Oceania	64.80	18.39	2.80	11.56	2.45
**ERR1019049**	38.5	LP6005441-DNA_A03	Bougainville	Oceania	67.08	16.99	4.03	9.98	1.93
**ERR1395554^**	51.0	LP6005519-DNA_E06	Dusun	Oceania	97.02	0.88	1.05	0.88	0.18
**ERR1395557**	53.0	LP6005519-DNA_F06	Dusun	Oceania	96.67	1.05	0.53	1.40	0.35
**ERR1395580^**	46.8	LP6005592-DNA_H03	Hawaiian	Oceania	92.12	1.23	0.88	5.25	0.53
**ERR1395546^**	40.6	LP6005519-DNA_C06	Igorot	Oceania	97.02	1.23	0.53	0.88	0.35
**ERR1395551**	48.9	LP6005519-DNA_D06	Igorot	Oceania	96.15	0.88	0.53	2.28	0.18
**ERR1395566^**	40.6	LP6005592-DNA_B02	Maori	Oceania	42.73	15.24	15.06	10.51	16.46
**ERR1019080^**	46.4	LP6005441-DNA_B10	Papuan	Oceania	56.92	24.87	3.68	11.91	2.63
**ERR1347685**	46.0	LP6005443-DNA_A08	Papuan	Oceania	52.19	26.27	5.25	13.66	2.63
**ERR1347693**	42.7	LP6005443-DNA_B08	Papuan	Oceania	53.24	26.80	4.38	12.78	2.80
**ERR1347701**	43.3	LP6005443-DNA_C07	Papuan	Oceania	55.69	26.80	3.68	11.91	1.93
**ERR1347702**	45.0	LP6005443-DNA_C08	Papuan	Oceania	54.99	26.44	3.85	12.61	2.10
**ERR1347710**	52.3	LP6005443-DNA_D07	Papuan	Oceania	53.94	26.27	4.03	14.01	1.75
**ERR1347711**	44.1	LP6005443-DNA_D08	Papuan	Oceania	53.77	26.44	5.25	12.78	1.75
**ERR1347717**	49.9	LP6005443-DNA_E07	Papuan	Oceania	53.77	24.34	4.55	15.41	1.93
**ERR1347718**	35.9	LP6005443-DNA_E08	Papuan	Oceania	52.54	26.97	4.38	13.84	2.28
**ERR1347727**	49.8	LP6005443-DNA_F07	Papuan	Oceania	53.42	27.50	4.55	13.31	1.23
**ERR1347728**	44.1	LP6005443-DNA_F08	Papuan	Oceania	54.47	27.15	3.15	12.61	2.63
**ERR1347736**	45.4	LP6005443-DNA_G07	Papuan	Oceania	55.17	25.92	3.85	13.31	1.75
**ERR1395595**	47.3	SS6004472	Papuan	Oceania	50.61	28.20	3.68	15.06	2.45
**ERR1395605**	48.0	LP6005443-DNA_H07	Papuan	Oceania	54.29	26.97	3.50	13.66	1.58
**ERR1419095**	35.1	LP6005441-DNA_A10	Papuan	Oceania	56.57	23.82	4.73	12.26	2.63
**ERR1019035^**	43.0	LP6005441-DNA_D01	Balochi	SouthAsia	3.50	62.87	7.36	11.21	15.06
**ERR1419110**	43.3	LP6005441-DNA_C01	Balochi	SouthAsia	3.68	63.22	7.88	7.36	17.86
**ERR1347669^**	44.5	LP6005442-DNA_G09	Bengali	SouthAsia	15.41	73.56	3.50	3.85	3.68
**ERR1347676**	34.0	LP6005442-DNA_H09	Bengali	SouthAsia	14.36	73.91	4.03	4.38	3.33
**ERR1395559^**	52.9	LP6005519-DNA_G03	Brahmin	SouthAsia	8.93	75.31	2.80	5.78	7.18

**ERR1395561**	36.6	LP6005519-DNA_H03	Brahmin	SouthAsia	7.53	76.01	4.03	7.36	5.08
**ERR1419112^**	35.7	LP6005441-DNA_C03	Brahui	SouthAsia	6.65	54.29	11.03	8.06	19.96
**ERR1419122**	38.3	LP6005441-DNA_D03	Brahui	SouthAsia	4.20	55.87	9.28	10.51	20.14
**ERR1025616^**	42.3	LP6005441-DNA_E03	Burusho	SouthAsia	12.96	60.07	7.53	5.95	13.49
**ERR1419142**	41.6	LP6005441-DNA_F03	Burusho	SouthAsia	9.98	60.77	7.36	6.65	15.24
**ERR1025625^**	64.8	LP6005441-DNA_F05	Hazara	SouthAsia	49.39	22.59	10.16	6.13	11.73
**ERR1419134**	67.7	LP6005441-DNA_E05	Hazara	SouthAsia	50.96	23.82	9.98	5.60	9.63
**ERR1395545^**	50.2	LP6005519-DNA_C05	Irula	SouthAsia	13.13	77.58	1.93	5.60	1.75
**ERR1395550**	53.1	LP6005519-DNA_D05	Irula	SouthAsia	12.78	76.71	2.63	5.25	2.63
**ERR1025633^**	37.3	LP6005441-DNA_E06	Kalash	SouthAsia	4.90	60.60	11.03	5.25	18.21
**ERR1419144**	34.9	LP6005441-DNA_F06	Kalash	SouthAsia	6.30	57.27	10.16	8.06	18.21
**ERR1395608^**	52.1	LP6005519-DNA_A04	Kapu	SouthAsia	10.33	79.33	2.45	4.20	3.68
**ERR1395611**	52.8	LP6005519-DNA_B04	Kapu	SouthAsia	16.11	74.96	2.98	4.03	1.93
**ERR1395553^**	50.3	LP6005519-DNA_E05	Khonda Dora	SouthAsia	50.09	43.78	1.23	4.55	0.35
**ERR1347703^**	45.4	LP6005443-DNA_C09	Kusunda	SouthAsia	71.80	20.84	2.98	3.68	0.70
**ERR1347712**	44.4	LP6005443-DNA_D09	Kusunda	SouthAsia	75.83	17.86	2.10	3.50	0.70
**ERR1395560^**	47.5	LP6005519-DNA_G04	Madiga	SouthAsia	10.16	80.21	2.80	4.03	2.80
**ERR1395562**	49.5	LP6005519-DNA_H04	Madiga	SouthAsia	11.56	79.33	1.75	5.25	2.10
**ERR1019059^**	40.3	LP6005441-DNA_D07	Makrani	SouthAsia	2.80	52.19	9.98	14.01	21.02
**ERR1419115**	43.2	LP6005441-DNA_C07	Makrani	SouthAsia	1.93	56.74	11.38	8.93	21.02
**ERR1395552^**	49.6	LP6005519-DNA_E04	Mala	SouthAsia	11.03	80.39	1.58	4.55	2.45
**ERR1395556**	48.4	LP6005519-DNA_F04	Mala	SouthAsia	11.03	81.26	2.10	3.85	1.75
**ERR1019065^**	42.4	LP6005441-DNA_D10	Pathan	SouthAsia	4.73	70.23	6.65	4.90	13.49
**ERR1419118**	41.4	LP6005441-DNA_C10	Pathan	SouthAsia	3.68	65.15	7.53	6.30	17.34
**ERR1025664^**	34.0	LP6005442-DNA_B12	Punjabi	SouthAsia	9.98	80.21	2.45	3.68	3.68
**ERR1395564**	35.3	LP6005592-DNA_A04	Punjabi	SouthAsia	8.41	67.60	6.48	5.08	12.43
**ERR1395568**	35.0	LP6005592-DNA_B04	Punjabi	SouthAsia	8.93	80.04	3.68	4.20	3.15
**ERR1419177**	39.0	LP6005442-DNA_A12	Punjabi	SouthAsia	8.58	81.09	2.28	4.38	3.68
**ERR1395609^**	46.8	LP6005519-DNA_A05	Relli	SouthAsia	19.79	71.98	2.28	4.38	1.58
**ERR1395612**	49.4	LP6005519-DNA_B05	Relli	SouthAsia	10.86	77.58	3.50	4.73	3.33
**ERR1025648^**	40.0	LP6005441-DNA_G11	Sindhi	SouthAsia	5.25	67.78	7.88	5.78	13.31
**ERR1025649**	39.4	LP6005441-DNA_H11	Sindhi	SouthAsia	4.55	74.26	5.60	7.18	8.41
**ERR1395549^**	50.2	LP6005519-DNA_D04	Yadava	SouthAsia	8.76	81.26	2.80	4.90	2.28
**ERR1395615**	55.5	LP6005519-DNA_C04	Yadava	SouthAsia	9.98	80.21	1.75	5.08	2.98

**ERR1025608^**	37.9	LP6005442-DNA_C02	Abkhasian	WestEurasia	5.08	28.20	13.49	6.48	46.76
**ERR1419191**	39.6	LP6005442-DNA_D02	Abkhasian	WestEurasia	4.20	27.85	12.08	7.71	48.16
**ERR1419088^**	41.7	LP6005441-DNA_A01	Adygei	WestEurasia	2.63	26.44	13.66	6.30	50.96
**ERR1419098**	35.4	LP6005441-DNA_B01	Adygei	WestEurasia	4.38	24.87	12.96	7.88	49.91
**ERR1395585^**	38.2	LP6005677-DNA_B01	Albanian	WestEurasia	1.40	14.19	15.06	7.53	61.82
**ERR1347665^**	40.4	LP6005442-DNA_G02	Armenian	WestEurasia	1.75	24.69	16.64	7.18	49.74
**ERR1395555**	46.2	LP6005519-DNA_F03	Armenian	WestEurasia	1.40	26.80	16.46	8.58	46.76
**ERR1019045^**	42.5	LP6005441-DNA_C02	Basque	WestEurasia	1.58	8.58	15.41	5.08	69.35
**ERR1019046**	38.8	LP6005441-DNA_D02	Basque	WestEurasia	1.23	7.71	17.86	6.30	66.90
**ERR1019047^**	42.3	LP6005441-DNA_F02	BedouinB	WestEurasia	2.28	15.24	23.12	19.09	40.28
**ERR1419132**	44.7	LP6005441-DNA_E02	BedouinB	WestEurasia	2.45	16.99	21.37	18.21	40.98
**ERR1019036^**	80.3	LP6005441-DNA_B06	Bergamo	WestEurasia	2.45	12.43	15.59	7.01	62.52
**ERR1419092**	70.9	LP6005441-DNA_A06	Bergamo	WestEurasia	1.75	10.68	15.06	8.58	63.92
**ERR1025614^**	42.4	LP6005442-DNA_A03	Bulgarian	WestEurasia	1.93	13.84	14.36	6.83	63.05
**ERR1025615**	37.9	LP6005442-DNA_B03	Bulgarian	WestEurasia	2.10	12.26	14.71	5.60	65.32
**ERR1025599^**	46.6	LP6005442-DNA_D03	Chechen	WestEurasia	3.15	26.44	16.81	7.36	46.23
**ERR1395617^**	44.1	LP6007068-DNA_A01	Crete	WestEurasia	2.63	17.16	15.59	9.63	54.99
**ERR1425293**	43.7	LP6007069-DNA_A01	Crete	WestEurasia	2.63	15.59	15.94	8.93	56.92
**ERR1395604^**	50.9	LP6005443-DNA_H05	Czech	WestEurasia	1.75	10.33	17.34	6.83	63.75
**ERR1347704^**	51.2	LP6005443-DNA_D01	Druze	WestEurasia	1.23	20.32	14.71	15.41	48.34
**ERR1419152**	45.0	LP6005441-DNA_G04	Druze	WestEurasia	2.10	21.54	17.51	12.08	46.76
**ERR1347661^**	46.0	LP6005442-DNA_F10	English	WestEurasia	1.75	8.76	13.66	5.08	70.75
**ERR1419199**	41.7	LP6005442-DNA_E10	English	WestEurasia	1.93	8.58	13.31	6.48	69.70
**ERR1347666^**	35.6	LP6005442-DNA_G03	Estonian	WestEurasia	2.80	8.76	14.89	5.60	67.95
**ERR1347674**	38.6	LP6005442-DNA_H03	Estonian	WestEurasia	3.33	10.86	13.84	5.43	66.55
**ERR1025659^**	38.2	LP6005442-DNA_D10	Finnish	WestEurasia	4.90	7.88	15.59	4.38	67.25
**ERR1346534**	35.2	LP6005442-DNA_C10	Finnish	WestEurasia	3.50	10.86	12.26	6.13	67.25
**ERR1395563**	47.9	LP6005592-DNA_A02	Finnish	WestEurasia	3.68	11.56	12.96	5.43	66.37
**ERR1019053^**	44.0	LP6005441-DNA_A05	French	WestEurasia	1.58	9.98	16.46	6.30	65.67
**ERR1019054**	39.9	LP6005441-DNA_B05	French	WestEurasia	0.88	9.98	17.51	5.60	66.02
**ERR1395619**	45.9	SS6004468	French	WestEurasia	1.23	10.16	16.11	5.78	66.73
**ERR1025624^**	33.9	LP6005442-DNA_A04	Georgian	WestEurasia	1.93	27.15	13.49	7.71	49.74
**ERR1419181**	43.0	LP6005442-DNA_B04	Georgian	WestEurasia	1.93	26.44	15.06	8.41	48.16
**ERR1347668^**	37.9	LP6005442-DNA_G07	Greek	WestEurasia	1.93	14.19	13.66	7.88	62.35

**ERR1347683**	46.4	LP6005443-DNA_A06	Greek	WestEurasia	1.40	26.09	12.43	6.65	53.42
**ERR1025630^**	41.0	LP6005442-DNA_A08	Hungarian	WestEurasia	2.10	11.21	14.19	6.13	66.37
**ERR1419182**	40.1	LP6005442-DNA_B08	Hungarian	WestEurasia	3.15	11.56	15.41	5.43	64.45
**ERR1025631^**	38.7	LP6005442-DNA_D08	Icelandic	WestEurasia	2.28	11.38	14.36	4.73	67.25
**ERR1347691**	49.9	LP6005443-DNA_B06	Icelandic	WestEurasia	1.93	9.63	14.36	5.60	68.48
**ERR1025632^**	38.0	LP6005442-DNA_C04	Iranian	WestEurasia	3.50	34.15	13.66	11.56	37.13
**ERR1347695**	46.6	LP6005443-DNA_B10	Iranian	WestEurasia	3.15	35.38	13.49	9.98	38.00
**ERR1395576^**	43.1	LP6005592-DNA_F01	Iraqi Jew	WestEurasia	1.40	24.34	17.86	9.46	46.94
**ERR1395620**	48.4	LP6005592-DNA_E01	Iraqi Jew	WestEurasia	1.40	22.77	15.06	12.26	48.51
**ERR1025661^**	39.7	LP6005442-DNA_E04	Jordanian	WestEurasia	1.93	18.91	18.74	19.61	40.81
**ERR1347659**	36.6	LP6005442-DNA_F04	Jordanian	WestEurasia	2.10	17.16	23.12	18.74	38.88
**ERR1395578**	48.4	LP6005592-DNA_G03	Jordanian	WestEurasia	1.93	18.74	20.49	18.21	40.63
**ERR1347667^**	40.2	LP6005442-DNA_G04	Lezgin	WestEurasia	1.93	26.97	13.66	8.06	49.39
**ERR1347675**	40.4	LP6005442-DNA_H04	Lezgin	WestEurasia	1.75	28.20	15.24	7.18	47.64
**ERR1347720^**	44.0	LP6005443-DNA_E10	North Ossetian	WestEurasia	6.83	27.85	16.64	7.53	41.16
**ERR1347729**	47.3	LP6005443-DNA_F10	North Ossetian	WestEurasia	5.60	26.27	15.06	6.83	46.23
**ERR1395565^**	50.1	LP6005592-DNA_B01	Norwegian	WestEurasia	2.10	8.41	15.59	6.13	67.78
**ERR1019062^**	39.8	LP6005441-DNA_C09	Orcadian	WestEurasia	1.58	9.81	16.99	6.65	64.97
**ERR1419128**	39.8	LP6005441-DNA_D09	Orcadian	WestEurasia	0.88	10.68	14.36	5.78	68.30
**ERR1025662^**	42.1	LP6005441-DNA_G09	Palestinian	WestEurasia	1.93	19.44	20.84	17.69	40.11
**ERR1395567**	46.7	LP6005592-DNA_B03	Palestinian	WestEurasia	1.58	17.34	20.32	21.72	39.05
**ERR1419167**	41.2	LP6005441-DNA_H09	Palestinian	WestEurasia	1.05	15.59	21.72	18.91	42.73
**ERR1395575^**	50.3	LP6005592-DNA_E02	Polish	WestEurasia	2.45	12.43	13.49	7.36	64.27
**ERR1025605^**	39.0	LP6005441-DNA_G10	Russian	WestEurasia	6.48	14.19	16.99	5.95	56.39
**ERR1025606**	39.1	LP6005441-DNA_H10	Russian	WestEurasia	5.95	13.13	16.11	4.73	60.07
**ERR1395569^**	50.2	LP6005592-DNA_C01	Saami	WestEurasia	14.36	12.78	20.14	4.55	48.16
**ERR1395616**	45.3	LP6005592-DNA_D01	Saami	WestEurasia	19.26	11.38	19.79	5.95	43.61
**ERR1395574^**	43.0	LP6005592-DNA_D04	Samaritan	WestEurasia	1.05	19.44	19.61	13.49	46.41
**ERR1019068^**	37.2	LP6005441-DNA_C11	Sardinian	WestEurasia	0.53	8.06	14.89	8.23	68.30
**ERR1395597**	41.7	SS6004474	Sardinian	WestEurasia	1.40	8.23	15.94	8.93	65.50
**ERR1419130**	34.8	LP6005441-DNA_D11	Sardinian	WestEurasia	0.70	7.01	19.44	7.88	64.97
**ERR1419176^**	35.5	LP6005442-DNA_A11	Spanish	WestEurasia	1.40	8.76	16.29	8.58	64.97
**ERR1419184**	43.0	LP6005442-DNA_B11	Spanish	WestEurasia	0.53	9.28	16.64	9.46	64.10
**ERR1395548^**	48.1	LP6005519-DNA_D03	Tajik	WestEurasia	5.43	37.65	13.84	7.88	35.20

**ERR1395614**	54.2	LP6005519-DNA_C03	Tajik	WestEurasia	7.36	41.16	13.13	5.25	33.10
**ERR1395584^**	44.6	LP6005677-DNA_A03	Turkish	WestEurasia	6.30	25.57	18.04	7.53	42.56
**ERR1395587**	45.5	LP6005677-DNA_C03	Turkish	WestEurasia	7.18	23.47	14.71	9.46	45.18
**ERR1025653^**	40.2	LP6005441-DNA_H12	Tuscan	WestEurasia	1.75	10.68	14.01	8.58	64.97
**ERR1419160**	43.6	LP6005441-DNA_G12	Tuscan	WestEurasia	1.93	12.43	16.29	8.23	61.12
**ERR1395579^**	51.7	LP6005592-DNA_H01	Yemenite Jew	WestEurasia	2.28	17.16	21.02	17.69	41.86
**ERR1395621**	56.2	LP6005592-DNA_G01	Yemenite Jew	WestEurasia	2.28	15.94	21.02	18.91	41.86

***We selected a unique download Accession for each record (Library) to avoid duplicates. ^Population representatives (129 individuals) are plotted in Figure 1d when multiple individuals from the same population are identified, to minimize crowding on the display. ‡Half the WGS was downloaded and processed for this individual of American ancestry and ntRoot predictions, consistent with the region of origin (population Pima from America), are achievable on WGS data as low as 22-fold coverage.**

Table S7. ntRoot super-population level GAI summary on complete and draft genome sequences, with matching raw whole-genome read sequencing data (where applicable). The super-populations are EAS: East Asian, SAS: South Asian, AMR: Admixed American, AFR: African, EUR: European.

Individual	Type	Assigned Label	ntRoot GAI	SNV Count	Non-zero AF SNV Count	GAI score
**HuRef**	WGS	CEU(EUR)	EUR	2,636,477	2,592,289	0.4942
	WGA HuRef‡	CEU(EUR)	EUR	1,943,423	1,903,006	0.5361
	WGA HuRefPrime‡	CEU(EUR)	EUR	1,945,709	1,905,081	0.5355
**CN1**	WGA maternal	EAS	EAS	2,002,956	1,960,790	0.5756
	WGA paternal	EAS	EAS	2,011,544	1,969,732	0.5754
**HuRef/CN1 simulated diploid**	WGA	mixed	EAS	2,776,922	2,640,999	0.4840
**KOREF**	WGA*	EAS	EAS	1,996,558	1,953,649	0.5785
	WGS(Illumina)	EAS	EAS	2,622,427	2,578,255	0.5314
	WGS(PacBio SII)	EAS	EAS	2,672,832	2,629,079	0.5292
**NA01243**	WGS(Illumina)	AMR	AFR	3,266,892	3,180,440	0.4190
**NA24385**	WGA maternal	EUR	EUR	2,098,033	1,978,829	0.4842
	WGA paternal	EUR	EUR	2,096,296	1,980,081	0.4882
	WGS(Illumina)	EUR	EUR	2,904,614	2,773,521	0.4528
	WGS(ONT V14)	EUR	EUR	2,859,212	2,729,585	0.4541
	WGS(PacBio SII)	EUR	EUR	2,886,074	2,756,569	0.4539
**HG02055**	WGS(Illumina)	AFR	AFR	3,187,354	3,161,469	0.4402
	WGA(GoldRush*,^)	AFR	AFR	1,168,009	1,144,993	0.5060
	WGA(Shasta*,^)	AFR	AFR	2,055,892	2,035,138	0.4968
	WGA(Flye*,^)	AFR	AFR	2,123,506	2,102,196	0.4859
	WGA maternal	AFR	AFR	2,249,827	2,226,829	0.4892
	WGA paternal	AFR	AFR	2,271,615	2,253,601	0.4910

***Pseudohaploid assembly, assembled with WGS Oxford Nanopore datasets (Wong et al., 2022, Table 6). The mat/pat suffix indicates maternal and paternal haplotypes, respectively. ‡HuRef and HuRefPrime haploid WGAs each refer to the principal and alternate haplotypes, respectively.**

Table S8. ntRoot (unless specified) super-population level LAI-based ancestry fraction estimates on complete and draft genome sequences, with matching raw whole-genome read sequencing data (where applicable). The LAI fraction estimates were generated using 5Mbp tiles. The super-populations are EAS: East Asian, SAS: South Asian, AMR: Admixed American, AFR: African, EUR: European.

Individual	Type	Assigned Label	Predicted from LAI	LAI EAS fraction (%)	LAI SAS fraction (%)	LAI AMR fraction (%)	LAI AFR fraction (%)	LAI EUR fraction (%)
**HuRef**	WGS	CEU(EUR)	EUR	1.75	11.38	14.01	7.01	65.85
	WGA HuRef‡	CEU(EUR)	EUR	2.28	11.03	16.11	8.93	61.65
	WGA HuRefPrime‡	CEU(EUR)	EUR	2.45	11.91	15.76	9.11	60.77
**CN1**	WGA maternal	EAS	EAS	95.27	0.88	0.70	2.10	1.05
	WGA paternal	EAS	EAS	95.27	0.88	2.10	1.58	0.18
**HuRef/CN1 simulated diploid**	WGA	mixed	EAS	54.63	11.29	12.57	7.77	13.74
**KOREF**	WGA*	EAS	EAS	94.57	0.70	1.23	2.98	0.53
	WGS(Illumina)	EAS	EAS	97.72	0.53	0.53	0.88	0.35
	WGS(PacBio)	EAS	EAS	97.90	0.35	0.53	0.88	0.35
**NA24385**	WGA maternal	EUR	EUR	4.73	14.36	21.89	18.91	40.11
	WGA paternal	EUR	EUR	3.33	15.59	23.29	17.69	40.11
	WGS(Illumina)	EUR	EUR	1.58	14.01	22.94	16.64	44.83
	WGS(ONT V14)	EUR	EUR	1.23	13.84	23.47	17.34	44.13
	WGS(PacBio SII)	EUR	EUR	1.40	14.19	22.94	16.99	44.48
**NA01243**	WGS(Illumina)	AMR						
ntRoot			AFR	0.70	0.70	15.76	77.58	5.25
ADMIXTURE			AFR	0.00	0.00	29.12	70.87	0.00
**HG02055**	WGS(Illumina)	AFR	AFR	0.00	0.00	5.43	93.35	1.23
	WGA(GoldRush*,^)	AFR	AFR	0.18	1.05	4.90	93.17	0.70
	WGA(Shasta*,^)	AFR	AFR	0.18	0.35	5.78	92.47	1.23
	WGA(Flye*,^)	AFR	AFR	0.18	0.35	5.60	92.82	1.05
	WGA maternal	AFR	AFR	1.05	1.58	3.68	83.89	9.81
	WGA paternal	AFR	AFR	0.35	1.23	2.28	93.87	2.28

***Pseudohaploid assembly. ^Assembled with WGS Oxford Nanopore datasets (Wong et al., 2022, Table 6). The mat/pat suffix indicates maternal and paternal haplotypes, respectively. ‡HuRef and HuRefPrime haploid WGAs each refer to the principal and alternate haplotypes, respectively.**

Table S9. Resource usage for ntRoot, SNVstory and ADMIXTURE.

Individual	Type	Input sequence file size# (GB)	Wall-clock run time (h)	Peak RAM (GB)
**HuRef**	WGS~47X	75.0	1.30	82.05
	WGA HuRef‡	2.7	0.47	12.77
	WGA HuRefPrime‡	2.7	0.48	12.72
**CN1**	WGA maternal	2.9	0.53	12.99
	WGA paternal	2.8	0.55	12.79
**HuRef/CN1 simulated diploid+**	WGA	5.4	0.50	14.84
**KOREF**	WGS~42X (Illumina)	72.0	1.27	84.77
	WGS~38X (PacBio SII)	79.0	1.27	77.30
	WGA*	2.7	0.47	12.86
**NA24385 (HG002)**	WGA maternal	2.9	0.41	14.54
	WGA paternal	2.8	0.39	14.30
	WGS~60X(Illumina)	42.0	1.26	77.67
	WGS~72X(ONT V14)	177.0	2.15	148.19
	WGS~34X(PacBio SII)	39.0	1.19	75.90
**HG02055**	WGS~38X	26.0	0.98	66.48
	WGA(GoldRush*,^)	2.9	0.42	13.08
	WGA(Shasta*,^)	2.8	0.47	12.99
	WGA(Flye*,^)	2.8	0.47	12.90
	WGA maternal	2.9	0.48	12.99
	WGA paternal	2.9	0.49	12.82
**Validation set, n=266 1kGP**	WGS~30X	~26.6	1.14 +/- 0.11	67.29 +/- 1.68
**Benchmark set, n=100 1kGP**	WGS~30X	~27.2		
**ntRoot**			1.14 +/- 0.18	67.08 +/- 1.73
**SNVstory**			4.42 +/- 0.21	87.35 +/- 6.71
**ADMIXTURE**			273.70	449.15
**Discovery set, n=279 SGDP**	WGS~44X [min:22X-max:87X]	~116.1	1.60 +/- 0.22	68.19 +/- 7.32

***Pseudohaploid assembly. ^Assembled with WGS Oxford Nanopore datasets (Wong et al., 2022, Table 6). The mat/pat suffix indicates maternal and paternal haplotypes, respectively. ‡HuRef and HuRefPrime haploid WGAs each refer to the principal and alternate haplotypes, respectively. #The WGS datasets input file size is gzip compressed. +Unlike with other WGA input, we used a 2 x 2.7 Gbp (~5.4 Gbp) genome input for this ntRoot analysis.**

Table S10. Effect of HG002 (NA24385, Illumina) WGS coverage on ntRoot ancestry predictions. The largest ancestry fraction is shown with bold face.

Fold coverage	Read pairs sampled (M)	LAI EAS fraction (%)	LAI SAS fraction (%)	LAI AMR fraction (%)	LAI AFR fraction (%)	LAI EUR fraction (%)
59.9 (full)	179.9	1.58	14.01	22.94	16.64	**44.83**
55.0	165.0	1.40	14.01	23.12	17.16	**44.31**
50.0	150.0	1.58	14.01	23.29	17.16	**43.96**
45.0	135.0	1.40	14.01	23.12	17.34	**44.13**
40.0	120.0	1.23	13.84	23.29	17.69	**43.96**
35.0	105.0	1.23	14.54	23.12	17.51	**43.61**
30.0	90.0	1.23	13.66	23.82	18.21	**43.08**
25.0	75.0	1.05	13.84	23.12	18.56	**43.43**
20.0	60.0	1.05	14.36	23.64	18.21	**42.73**
17.5	52.5	1.23	14.71	23.29	18.21	**42.56**
15.0	45.0	1.23	14.54	23.47	18.39	**42.38**
12.5	37.5	1.23	14.36	23.82	18.39	**42.21**
10.0	30.0	0.70	0.88	0.70	**97.55**	0.18
7.5	22.5	0.35	1.05	1.40	**97.19**	0.00
5.0	15.0	0.35	1.05	1.05	**97.36**	0.18
2.5	7.5	0.70	1.23	1.23	**96.66**	0.18

Table S11. Rationale for selecting the parameter k in ntRoot. Performance (Sensitivity, Precision, F1, PRAUC: Precision-Recall Area Under the Curve) of ntRoot in identifying ancestry-discriminant SNVs against a benchmark set of known HG002 variants[5] as a function of parameter k (word length) and its effect on ancestry fraction predictions using the HG002 (NA24385, Illumina) WGS dataset. The largest ancestry fraction is shown in bold face.

k	Sensitivity	Precision	F1	PRAUC	LAI EAS fraction (%)	LAI SAS fraction (%)	LAI AMR fraction (%)	LAI AFR fraction (%)	LAI EUR fraction (%)
70	0.7739	0.8651	0.8170	0.0397	1.75	14.71	18.74	11.73	**53.06**
65	0.7830	0.8577	0.8186	0.0395	1.75	15.24	18.39	12.43	**52.19**
60	0.7956	0.8447	0.8194	0.0394	1.93	14.71	20.14	13.84	**49.39**
55	0.8051	0.8262	0.8155	0.0388	1.58	14.01	22.94	16.64	**44.83**
50	0.8093	0.8089	0.8091	0.0383	1.23	12.96	25.22	20.49	**40.11**
45	0.8189	0.7813	0.7997	0.0374	1.05	12.08	28.02	23.64	**35.20**
40	0.8287	0.7354	0.7793	0.0355	0.88	9.98	**31.87**	31.17	26.09
35	0.8299	0.6599	0.7352	0.0340	0.70	9.28	31.52	**37.48**	21.02
30	0.8302	0.7034	0.7616	0.0323	0.53	7.18	31.00	**47.81**	13.49

Table S12. Rationale for selecting the parameter Y in ntRoot. Performance (Sensitivity, Precision, F1, PRAUC: Precision-Recall Area Under the Curve) of ntRoot in identifying ancestry-discriminant SNVs against a benchmark set of known HG002 variants[5] as a function of the parameter Y (Bloom filter hit fraction threshold) and its effect on ancestry fraction predictions using the HG002 (NA24385, Illumina) WGS dataset. The largest ancestry fraction is shown in bold face.

Y	Sensitivity	Precision	F1	PRAUC	LAI EAS fraction (%)	LAI SAS fraction (%)	LAI AMR fraction (%)	LAI AFR fraction (%)	LAI EUR fraction (%)
0.10	0.8675	0.7496	0.8042	0.0356	0.70	11.38	30.47	25.92	**31.52**
0.15	0.8596	0.7625	0.8081	0.0361	0.70	12.08	29.25	24.87	**33.10**
0.20	0.8523	0.7721	0.8102	0.0364	1.05	11.73	28.20	23.82	**35.20**
0.25	0.8452	0.7816	0.8121	0.0368	1.05	12.78	26.97	22.94	**36.25**
0.30	0.8380	0.7904	0.8135	0.0371	1.05	13.31	25.74	21.72	**38.18**
0.35	0.8312	0.7987	0.8146	0.0375	1.05	13.49	25.22	20.84	**39.40**
0.40	0.8245	0.8061	0.8152	0.0378	1.23	13.84	25.04	19.44	**40.46**
0.45	0.8180	0.8131	0.8155	0.0381	1.23	13.49	24.52	18.91	**41.86**
0.50	0.8114	0.8200	0.8157	0.0384	1.40	14.01	23.29	18.04	**43.26**
0.55	0.8051	0.8262	0.8155	0.0388	1.58	14.01	22.94	16.64	**44.83**
0.60	0.7986	0.8324	0.8152	0.0390	1.58	14.36	22.77	16.64	**44.66**
0.65	0.7951	0.8350	0.8150	0.0392	1.58	14.36	22.75	16.64	**44.67**
0.70	0.7924	0.8383	0.8147	0.0394	1.58	14.36	21.89	15.59	**46.58**
0.75	0.7861	0.8440	0.8140	0.0397	1.58	14.36	21.37	15.24	**47.46**
0.80	0.7800	0.8495	0.8133	0.0400	1.58	14.71	19.79	14.54	**49.39**
0.85	0.7738	0.8548	0.8123	0.0404	1.75	15.06	19.44	13.31	**50.44**
0.90	0.7675	0.8599	0.8111	0.0406	1.93	15.06	18.74	12.78	**51.49**
0.95	0.7612	0.8650	0.8098	0.0409	1.93	14.71	18.74	12.43	**52.19**
1.00	0.7552	0.8699	0.8085	0.0411	1.93	14.71	18.56	12.08	**52.71**

Table S13. Performance of ntRoot ancestry fraction predictions as a function of parameters k and Y on the balanced 1kGP WGS benchmark set of 100 samples (20 from each super-population). Metrics reported include ancestry fractions, precision, recall, F1 score, AUC, and overall accuracy across all classes for Y=0.55, k = 45, 55, and 65 and k=55, Y=0.45 and 0.65. Across all five super-populations (EAS: East Asian, SAS: South Asian, AMR: Admixed American, AFR: African, EUR: European), predictions were highly accurate, with precision, recall, F1, and per-class AUC values generally at or near 1.0, indicating robust and consistent performance across k values and populations.

ACCESSION	ID	1kGP assi-gned Label	k45 Y0.55					k55 Y0.55					k65 Y0.55					k55 Y0.45					k55 Y0.65				
			EAS	SAS	AMR	AFR	EUR	EAS	SAS	AMR	AFR	EUR	EAS	SAS	AMR	AFR	EUR	EAS	SAS	AMR	AFR	EUR	EAS	SAS	AMR	AFR	EUR
**ERR3242231**	HG02282	AFR	0.00	0.00	0.05	0.93	0.02	0.00	0.00	0.06	0.91	0.03	0.00	0.01	0.06	0.92	0.02	0.00	0.01	0.05	0.92	0.02	0.00	0.01	0.05	0.92	0.02
**ERR3989003**	HG02257	AFR	0.00	0.00	0.03	0.96	0.01	0.00	0.00	0.04	0.95	0.01	0.00	0.00	0.04	0.95	0.01	0.00	0.00	0.04	0.96	0.01	0.00	0.00	0.04	0.96	0.01
**ERR3242485**	HG02974	AFR	0.00	0.00	0.00	1.00	0.00	0.00	0.00	0.00	1.00	0.00	0.00	0.00	0.00	1.00	0.00	0.00	0.00	0.00	1.00	0.00	0.00	0.00	0.00	1.00	0.00
**ERR3989126**	HG03125	AFR	0.00	0.00	0.00	1.00	0.00	0.00	0.00	0.00	1.00	0.00	0.00	0.00	0.00	0.99	0.00	0.00	0.00	0.00	0.99	0.00	0.00	0.00	0.00	0.99	0.00
**ERR3989162**	HG03371	AFR	0.00	0.00	0.00	1.00	0.00	0.00	0.00	0.00	1.00	0.00	0.00	0.00	0.00	1.00	0.00	0.00	0.00	0.00	1.00	0.00	0.00	0.00	0.00	1.00	0.00
**ERR3989174**	HG03516	AFR	0.00	0.00	0.00	1.00	0.00	0.00	0.00	0.00	1.00	0.00	0.00	0.00	0.00	1.00	0.00	0.00	0.00	0.00	1.00	0.00	0.00	0.00	0.00	1.00	0.00
**ERR3242376**	HG02614	AFR	0.00	0.00	0.01	0.99	0.00	0.00	0.00	0.01	0.99	0.00	0.00	0.00	0.01	0.99	0.00	0.00	0.00	0.01	0.99	0.00	0.00	0.00	0.01	0.99	0.00
**ERR3242458**	HG02817	AFR	0.00	0.00	0.01	0.99	0.00	0.00	0.00	0.01	0.99	0.00	0.00	0.00	0.01	0.99	0.00	0.00	0.00	0.01	0.99	0.00	0.00	0.00	0.01	0.99	0.00
**ERR3989031**	HG02587	AFR	0.00	0.00	0.01	0.99	0.00	0.00	0.00	0.01	1.00	0.00	0.00	0.00	0.01	0.99	0.00	0.00	0.00	0.01	0.99	0.00	0.00	0.00	0.01	0.99	0.00
**ERR3989060**	HG02723	AFR	0.00	0.00	0.01	0.99	0.00	0.00	0.00	0.01	0.99	0.00	0.00	0.00	0.01	0.99	0.00	0.00	0.00	0.01	0.99	0.00	0.00	0.00	0.01	0.99	0.00
**ERR3989080**	HG02818	AFR	0.00	0.00	0.01	0.99	0.00	0.00	0.00	0.01	0.99	0.00	0.00	0.00	0.01	0.99	0.00	0.00	0.00	0.01	0.99	0.00	0.00	0.00	0.01	0.99	0.00
**ERR3989091**	HG02886	AFR	0.00	0.00	0.00	1.00	0.00	0.00	0.00	0.00	1.00	0.00	0.00	0.00	0.00	1.00	0.00	0.00	0.00	0.00	1.00	0.00	0.00	0.00	0.00	1.00	0.00
**ERR3239692**	NA19030	AFR	0.00	0.00	0.00	1.00	0.00	0.00	0.00	0.01	0.99	0.00	0.00	0.00	0.01	0.99	0.00	0.00	0.00	0.01	0.99	0.00	0.00	0.00	0.01	0.99	0.00
**ERR3239700**	NA19043	AFR	0.00	0.00	0.01	0.99	0.00	0.00	0.00	0.01	0.99	0.00	0.00	0.00	0.01	0.99	0.00	0.00	0.00	0.01	0.99	0.00	0.00	0.00	0.01	0.99	0.00
**ERR3242560**	HG03069	AFR	0.00	0.00	0.00	1.00	0.00	0.00	0.00	0.00	1.00	0.00	0.00	0.00	0.00	1.00	0.00	0.00	0.00	0.00	1.00	0.00	0.00	0.00	0.00	1.00	0.00
**ERR3242571**	HG03548	AFR	0.00	0.00	0.00	1.00	0.00	0.00	0.00	0.00	1.00	0.00	0.00	0.00	0.00	1.00	0.00	0.00	0.00	0.00	1.00	0.00	0.00	0.00	0.00	1.00	0.00
**ERR3989118**	HG03065	AFR	0.00	0.00	0.00	1.00	0.00	0.00	0.00	0.01	1.00	0.00	0.00	0.00	0.01	0.99	0.00	0.00	0.00	0.00	1.00	0.00	0.00	0.00	0.00	1.00	0.00
**ERR3989180**	HG03579	AFR	0.00	0.00	0.01	0.99	0.00	0.00	0.00	0.01	1.00	0.00	0.00	0.00	0.01	0.99	0.00	0.00	0.00	0.01	0.99	0.00	0.00	0.00	0.01	0.99	0.00
**ERR3239351**	NA18522	AFR	0.00	0.00	0.00	1.00	0.00	0.00	0.00	0.01	0.99	0.00	0.00	0.00	0.01	0.99	0.00	0.00	0.00	0.00	0.99	0.00	0.00	0.00	0.01	0.99	0.00
**ERR3239453**	NA19238	AFR	0.00	0.00	0.00	1.00	0.00	0.00	0.00	0.00	1.00	0.00	0.00	0.00	0.00	1.00	0.00	0.00	0.00	0.00	1.00	0.00	0.00	0.00	0.00	1.00	0.00
**ERR3242786**	HG01122	AMR	0.02	0.05	0.53	0.21	0.19	0.04	0.05	0.48	0.11	0.32	0.04	0.05	0.49	0.12	0.31	0.03	0.05	0.52	0.16	0.25	0.03	0.05	0.50	0.14	0.28
**ERR3242795**	HG01363	AMR	0.04	0.02	0.50	0.35	0.09	0.07	0.04	0.48	0.27	0.14	0.07	0.03	0.48	0.27	0.14	0.05	0.03	0.50	0.32	0.11	0.06	0.02	0.49	0.30	0.13
**ERR3242796**	HG01369	AMR	0.06	0.02	0.57	0.28	0.06	0.10	0.03	0.56	0.21	0.10	0.09	0.03	0.57	0.22	0.10	0.07	0.02	0.59	0.23	0.08	0.08	0.03	0.58	0.22	0.09
**ERR3242797**	HG01372	AMR	0.04	0.04	0.60	0.20	0.12	0.06	0.04	0.60	0.11	0.20	0.06	0.04	0.60	0.11	0.19	0.05	0.04	0.61	0.16	0.14	0.05	0.04	0.61	0.13	0.17
**ERR3242805**	HG01468	AMR	0.02	0.06	0.50	0.22	0.22	0.03	0.07	0.46	0.09	0.35	0.03	0.07	0.47	0.09	0.34	0.02	0.07	0.49	0.15	0.27	0.02	0.07	0.48	0.12	0.30
**ERR3988843**	HG01114	AMR	0.01	0.05	0.51	0.18	0.25	0.03	0.06	0.47	0.06	0.39	0.03	0.06	0.47	0.06	0.38	0.01	0.05	0.50	0.12	0.31	0.02	0.05	0.49	0.10	0.34
**ERR3988876**	HG01361	AMR	0.03	0.05	0.54	0.22	0.16	0.07	0.06	0.50	0.11	0.26	0.05	0.06	0.53	0.12	0.24	0.04	0.06	0.54	0.17	0.19	0.04	0.06	0.53	0.15	0.21
**ERR3988882**	HG01433	AMR	0.04	0.03	0.52	0.25	0.16	0.05	0.04	0.48	0.16	0.27	0.05	0.03	0.49	0.17	0.26	0.04	0.03	0.50	0.21	0.21	0.04	0.04	0.50	0.20	0.23
**ERR3988889**	HG01496	AMR	0.06	0.02	0.57	0.29	0.05	0.10	0.04	0.56	0.19	0.11	0.09	0.03	0.57	0.20	0.11	0.07	0.02	0.58	0.26	0.07	0.07	0.03	0.58	0.24	0.09
**ERR3989413**	NA19650	AMR	0.02	0.06	0.44	0.22	0.26	0.05	0.06	0.42	0.08	0.39	0.05	0.06	0.40	0.09	0.41	0.04	0.06	0.42	0.15	0.34	0.04	0.06	0.41	0.12	0.37
**ERR3242814**	HG02102	AMR	0.15	0.02	0.74	0.09	0.01	0.24	0.01	0.70	0.03	0.02	0.22	0.01	0.71	0.04	0.02	0.17	0.02	0.75	0.06	0.01	0.20	0.02	0.72	0.05	0.02
**ERR3988951**	HG01928	AMR	0.19	0.01	0.75	0.06	0.00	0.31	0.01	0.67	0.01	0.00	0.30	0.01	0.69	0.01	0.00	0.24	0.01	0.73	0.02	0.00	0.26	0.01	0.71	0.02	0.00
**ERR3988952**	HG01934	AMR	0.14	0.01	0.67	0.14	0.03	0.22	0.02	0.64	0.07	0.05	0.22	0.02	0.64	0.08	0.05	0.18	0.02	0.67	0.11	0.04	0.19	0.02	0.66	0.09	0.04
**ERR3988955**	HG01943	AMR	0.15	0.01	0.73	0.10	0.00	0.27	0.01	0.67	0.04	0.01	0.24	0.01	0.70	0.04	0.01	0.18	0.01	0.73	0.07	0.01	0.20	0.01	0.72	0.05	0.01
**ERR3988958**	HG01952	AMR	0.18	0.00	0.69	0.13	0.00	0.26	0.01	0.67	0.06	0.01	0.26	0.00	0.67	0.07	0.01	0.21	0.00	0.70	0.09	0.00	0.22	0.00	0.70	0.08	0.01
**ERR3988964**	HG01975	AMR	0.16	0.01	0.72	0.10	0.00	0.26	0.01	0.69	0.04	0.00	0.24	0.01	0.70	0.05	0.00	0.20	0.01	0.72	0.07	0.00	0.22	0.01	0.71	0.06	0.00
**ERR3241754**	HG00731	AMR	0.00	0.04	0.47	0.22	0.26	0.02	0.05	0.43	0.09	0.41	0.01	0.06	0.42	0.10	0.41	0.01	0.05	0.45	0.17	0.32	0.01	0.05	0.44	0.13	0.36
**ERR3241755**	HG00732	AMR	0.02	0.06	0.44	0.27	0.21	0.03	0.06	0.42	0.15	0.34	0.03	0.06	0.43	0.15	0.32	0.02	0.05	0.46	0.21	0.26	0.02	0.05	0.44	0.19	0.29
**ERR3242761**	HG01395	AMR	0.00	0.04	0.50	0.26	0.20	0.02	0.06	0.46	0.15	0.31	0.02	0.05	0.47	0.16	0.30	0.01	0.05	0.50	0.20	0.24	0.01	0.05	0.49	0.18	0.26
**ERR3242766**	HG01414	AMR	0.01	0.04	0.50	0.31	0.14	0.03	0.04	0.49	0.20	0.24	0.03	0.04	0.49	0.21	0.23	0.02	0.04	0.50	0.26	0.18	0.02	0.04	0.50	0.24	0.19
**ERR3242132**	HG00864	EAS	0.91	0.02	0.02	0.05	0.00	0.97	0.01	0.01	0.01	0.00	0.97	0.01	0.01	0.01	0.00	0.94	0.01	0.01	0.04	0.00	0.95	0.01	0.01	0.03	0.00
**ERR3242142**	HG01046	EAS	0.92	0.01	0.01	0.06	0.00	0.98	0.00	0.01	0.00	0.00	0.98	0.01	0.01	0.01	0.00	0.95	0.01	0.01	0.03	0.00	0.95	0.01	0.01	0.02	0.00
**ERR3242150**	HG01801	EAS	0.91	0.01	0.01	0.07	0.00	0.97	0.01	0.01	0.01	0.00	0.97	0.01	0.01	0.01	0.00	0.95	0.01	0.01	0.03	0.00	0.96	0.01	0.01	0.03	0.00
**ERR3242160**	HG01812	EAS	0.92	0.01	0.02	0.06	0.00	0.98	0.01	0.01	0.01	0.00	0.98	0.01	0.01	0.01	0.00	0.95	0.01	0.01	0.03	0.00	0.97	0.01	0.01	0.01	0.00
**ERR3241678**	HG00473	EAS	0.93	0.00	0.02	0.05	0.00	0.98	0.01	0.01	0.01	0.01	0.98	0.01	0.01	0.01	0.00	0.96	0.00	0.01	0.02	0.00	0.97	0.00	0.01	0.02	0.00
**ERR3241684**	HG00513	EAS	0.93	0.01	0.01	0.05	0.00	0.98	0.01	0.00	0.01	0.00	0.98	0.01	0.00	0.01	0.00	0.96	0.01	0.00	0.02	0.00	0.97	0.01	0.00	0.02	0.00
**ERR3241686**	HG00525	EAS	0.92	0.01	0.01	0.06	0.00	0.97	0.01	0.01	0.01	0.00	0.97	0.01	0.01	0.02	0.00	0.95	0.01	0.01	0.03	0.00	0.96	0.01	0.01	0.03	0.00

**ERR3241750**	HG00705	EAS	0.91	0.01	0.01	0.07	0.00	0.98	0.00	0.00	0.01	0.00	0.98	0.00	0.00	0.01	0.00	0.95	0.01	0.01	0.04	0.00	0.97	0.01	0.00	0.01	0.00
**ERR3242512**	HG00410	EAS	0.92	0.00	0.02	0.06	0.00	0.98	0.00	0.01	0.01	0.00	0.98	0.00	0.01	0.01	0.00	0.95	0.00	0.02	0.03	0.00	0.96	0.00	0.01	0.03	0.00
**ERR3242514**	HG00599	EAS	0.92	0.01	0.02	0.06	0.00	0.98	0.00	0.01	0.01	0.00	0.98	0.00	0.01	0.01	0.00	0.95	0.01	0.01	0.03	0.00	0.96	0.01	0.01	0.03	0.00
**ERR3242515**	HG00728	EAS	0.92	0.01	0.01	0.06	0.00	0.98	0.01	0.01	0.01	0.00	0.98	0.01	0.01	0.01	0.00	0.95	0.01	0.01	0.03	0.00	0.96	0.01	0.01	0.02	0.00
**ERR3242519**	HG00631	EAS	0.93	0.00	0.02	0.05	0.00	0.98	0.01	0.01	0.01	0.00	0.98	0.01	0.01	0.01	0.00	0.96	0.00	0.01	0.02	0.00	0.97	0.01	0.01	0.01	0.00
**ERR3242522**	HG00675	EAS	0.91	0.01	0.01	0.06	0.00	0.97	0.01	0.01	0.02	0.00	0.97	0.01	0.01	0.02	0.00	0.95	0.01	0.01	0.04	0.00	0.96	0.01	0.01	0.03	0.00
**ERR3988762**	HG00408	EAS	0.91	0.01	0.01	0.06	0.00	0.98	0.01	0.00	0.01	0.00	0.98	0.01	0.01	0.01	0.00	0.95	0.01	0.01	0.03	0.00	0.96	0.01	0.01	0.02	0.00
**ERR3988765**	HG00423	EAS	0.93	0.01	0.01	0.06	0.00	0.98	0.00	0.01	0.01	0.00	0.98	0.00	0.01	0.01	0.00	0.96	0.00	0.01	0.02	0.00	0.97	0.00	0.01	0.02	0.00
**ERR3988768**	HG00438	EAS	0.92	0.01	0.01	0.06	0.00	0.97	0.01	0.01	0.01	0.00	0.97	0.01	0.01	0.02	0.00	0.95	0.01	0.02	0.03	0.00	0.96	0.01	0.01	0.02	0.00
**ERR3988780**	HG00512	EAS	0.92	0.01	0.01	0.06	0.00	0.98	0.00	0.01	0.01	0.00	0.98	0.00	0.01	0.01	0.00	0.95	0.01	0.01	0.03	0.00	0.97	0.00	0.01	0.02	0.00
**ERR3242087**	HG01862	EAS	0.90	0.02	0.01	0.07	0.00	0.97	0.01	0.01	0.01	0.00	0.97	0.01	0.01	0.01	0.00	0.94	0.01	0.01	0.04	0.00	0.95	0.01	0.01	0.02	0.00
**ERR3242101**	HG02048	EAS	0.89	0.01	0.02	0.07	0.00	0.97	0.01	0.00	0.01	0.01	0.97	0.01	0.00	0.02	0.00	0.92	0.01	0.01	0.05	0.00	0.95	0.01	0.01	0.03	0.00
**ERR3242342**	HG02040	EAS	0.90	0.01	0.01	0.07	0.00	0.97	0.01	0.01	0.01	0.00	0.97	0.01	0.01	0.01	0.00	0.94	0.01	0.01	0.04	0.00	0.95	0.01	0.01	0.03	0.00
**ERR3239334**	NA12878	EUR	0.01	0.08	0.22	0.20	0.50	0.01	0.09	0.14	0.06	0.70	0.01	0.09	0.15	0.06	0.68	0.01	0.07	0.20	0.12	0.59	0.01	0.08	0.19	0.09	0.63
**ERR3240160**	HG00171	EUR	0.04	0.08	0.26	0.17	0.46	0.05	0.09	0.14	0.05	0.67	0.05	0.10	0.15	0.05	0.65	0.04	0.09	0.21	0.11	0.55	0.04	0.09	0.20	0.08	0.58
**ERR3240240**	HG00323	EUR	0.01	0.09	0.23	0.18	0.49	0.04	0.11	0.12	0.06	0.68	0.04	0.11	0.14	0.06	0.65	0.03	0.09	0.19	0.13	0.56	0.04	0.10	0.17	0.10	0.60
**ERR3241788**	HG00280	EUR	0.03	0.10	0.21	0.17	0.49	0.04	0.10	0.12	0.05	0.69	0.04	0.10	0.14	0.05	0.67	0.03	0.11	0.18	0.11	0.57	0.04	0.10	0.17	0.09	0.61
**ERR3241799**	HG00331	EUR	0.03	0.08	0.22	0.18	0.49	0.05	0.08	0.12	0.05	0.70	0.05	0.09	0.13	0.06	0.67	0.04	0.09	0.18	0.11	0.58	0.04	0.09	0.17	0.09	0.61
**ERR3240114**	HG00096	EUR	0.01	0.10	0.23	0.18	0.49	0.01	0.10	0.13	0.05	0.71	0.01	0.09	0.14	0.06	0.70	0.01	0.09	0.18	0.12	0.60	0.01	0.09	0.17	0.11	0.63
**ERR3240116**	HG00099	EUR	0.00	0.11	0.22	0.19	0.49	0.00	0.10	0.13	0.06	0.71	0.00	0.10	0.14	0.06	0.69	0.00	0.11	0.20	0.12	0.57	0.00	0.11	0.16	0.10	0.63
**ERR3240121**	HG00105	EUR	0.01	0.09	0.23	0.18	0.49	0.02	0.10	0.15	0.05	0.68	0.02	0.10	0.17	0.05	0.66	0.01	0.11	0.21	0.12	0.56	0.02	0.11	0.20	0.09	0.58
**ERR3240126**	HG00110	EUR	0.01	0.07	0.23	0.18	0.50	0.02	0.08	0.14	0.07	0.70	0.02	0.09	0.15	0.07	0.68	0.01	0.08	0.19	0.13	0.59	0.02	0.09	0.16	0.11	0.63
**ERR3240135**	HG00127	EUR	0.01	0.10	0.23	0.17	0.48	0.01	0.12	0.12	0.05	0.71	0.02	0.11	0.13	0.05	0.69	0.01	0.11	0.18	0.11	0.58	0.01	0.11	0.16	0.10	0.62
**ERR3240146**	HG00140	EUR	0.00	0.08	0.25	0.20	0.46	0.01	0.09	0.15	0.07	0.69	0.00	0.09	0.15	0.08	0.67	0.00	0.08	0.21	0.14	0.56	0.00	0.09	0.19	0.12	0.60
**ERR3240155**	HG00151	EUR	0.01	0.08	0.23	0.18	0.51	0.01	0.10	0.13	0.07	0.69	0.01	0.11	0.13	0.07	0.68	0.01	0.09	0.20	0.12	0.58	0.01	0.10	0.17	0.09	0.62
**ERR3240195**	HG00121	EUR	0.01	0.10	0.23	0.19	0.47	0.02	0.10	0.11	0.06	0.71	0.01	0.09	0.13	0.06	0.70	0.01	0.10	0.18	0.12	0.58	0.01	0.10	0.17	0.09	0.63
**ERR3242186**	HG01790	EUR	0.01	0.11	0.25	0.17	0.47	0.02	0.11	0.13	0.06	0.69	0.01	0.10	0.15	0.06	0.67	0.01	0.10	0.20	0.12	0.57	0.01	0.11	0.19	0.09	0.61
**ERR3241917**	HG01501	EUR	0.01	0.06	0.24	0.26	0.43	0.01	0.08	0.18	0.10	0.62	0.01	0.08	0.19	0.11	0.62	0.01	0.07	0.23	0.18	0.51	0.01	0.08	0.21	0.15	0.55
**ERR3241951**	HG01615	EUR	0.00	0.08	0.26	0.22	0.45	0.01	0.09	0.15	0.08	0.67	0.01	0.09	0.16	0.09	0.66	0.00	0.09	0.22	0.16	0.53	0.01	0.08	0.20	0.14	0.57
**ERR3242111**	HG01695	EUR	0.01	0.06	0.26	0.27	0.38	0.03	0.07	0.19	0.16	0.56	0.02	0.07	0.22	0.16	0.53	0.02	0.06	0.24	0.23	0.45	0.02	0.06	0.23	0.19	0.49
**ERR3988892**	HG01505	EUR	0.01	0.08	0.27	0.22	0.43	0.02	0.09	0.16	0.09	0.65	0.02	0.09	0.18	0.10	0.63	0.01	0.08	0.22	0.15	0.54	0.01	0.08	0.20	0.13	0.57
**ERR3239792**	NA20509	EUR	0.00	0.12	0.22	0.20	0.46	0.01	0.15	0.13	0.07	0.64	0.01	0.14	0.14	0.08	0.63	0.01	0.13	0.18	0.14	0.54	0.01	0.14	0.16	0.12	0.57
**ERR3239873**	NA20805	EUR	0.00	0.08	0.23	0.22	0.47	0.01	0.09	0.16	0.06	0.68	0.01	0.09	0.16	0.06	0.67	0.01	0.09	0.20	0.16	0.55	0.01	0.08	0.18	0.11	0.61
**ERR3989224**	HG03831	SAS	0.13	0.64	0.05	0.16	0.02	0.18	0.73	0.03	0.05	0.02	0.18	0.72	0.03	0.05	0.02	0.16	0.68	0.05	0.10	0.02	0.17	0.71	0.04	0.07	0.02
**ERR3989225**	HG03834	SAS	0.13	0.64	0.06	0.16	0.01	0.18	0.73	0.03	0.04	0.02	0.16	0.74	0.03	0.05	0.02	0.15	0.69	0.05	0.10	0.01	0.16	0.72	0.04	0.07	0.02
**ERR3240106**	NA20905	SAS	0.04	0.73	0.05	0.15	0.03	0.06	0.82	0.03	0.04	0.04	0.06	0.82	0.03	0.05	0.04	0.05	0.76	0.05	0.11	0.04	0.05	0.78	0.04	0.09	0.04
**ERR3243148**	HG03868	SAS	0.04	0.75	0.04	0.15	0.02	0.07	0.84	0.01	0.05	0.04	0.06	0.83	0.02	0.05	0.04	0.05	0.79	0.03	0.09	0.03	0.06	0.81	0.02	0.07	0.03
**ERR3989210**	HG03732	SAS	0.05	0.74	0.04	0.16	0.01	0.05	0.84	0.03	0.06	0.02	0.06	0.84	0.03	0.06	0.02	0.05	0.79	0.04	0.11	0.02	0.05	0.80	0.04	0.10	0.01
**ERR3242635**	HG03668	SAS	0.05	0.66	0.08	0.18	0.03	0.08	0.76	0.04	0.05	0.06	0.08	0.77	0.04	0.06	0.06	0.06	0.72	0.06	0.12	0.04	0.07	0.74	0.06	0.09	0.05
**ERR3243049**	HG02731	SAS	0.04	0.68	0.07	0.16	0.05	0.07	0.77	0.04	0.05	0.08	0.06	0.76	0.04	0.05	0.08	0.05	0.72	0.06	0.11	0.06	0.06	0.74	0.05	0.08	0.07
**ERR3243053**	HG03022	SAS	0.04	0.70	0.04	0.19	0.04	0.06	0.82	0.03	0.04	0.05	0.06	0.82	0.03	0.04	0.05	0.05	0.76	0.04	0.11	0.04	0.06	0.78	0.03	0.08	0.05
**ERR3989034**	HG02602	SAS	0.05	0.71	0.06	0.15	0.03	0.07	0.81	0.04	0.04	0.04	0.07	0.81	0.04	0.04	0.04	0.06	0.77	0.05	0.09	0.04	0.07	0.78	0.04	0.08	0.04
**ERR3989056**	HG02698	SAS	0.04	0.67	0.09	0.16	0.04	0.06	0.79	0.05	0.04	0.06	0.05	0.78	0.05	0.05	0.06	0.04	0.74	0.07	0.09	0.05	0.04	0.76	0.06	0.08	0.06
**ERR3989064**	HG02738	SAS	0.04	0.63	0.09	0.17	0.07	0.06	0.72	0.05	0.06	0.11	0.06	0.72	0.06	0.06	0.10	0.04	0.69	0.07	0.11	0.09	0.05	0.70	0.05	0.10	0.09
**ERR3989139**	HG03239	SAS	0.02	0.63	0.09	0.18	0.08	0.05	0.73	0.06	0.05	0.11	0.04	0.73	0.07	0.05	0.11	0.03	0.68	0.09	0.12	0.08	0.04	0.70	0.08	0.09	0.09
**ERR3989173**	HG03492	SAS	0.04	0.68	0.07	0.17	0.04	0.07	0.78	0.05	0.05	0.06	0.06	0.78	0.06	0.05	0.05	0.05	0.72	0.07	0.11	0.05	0.05	0.75	0.06	0.09	0.05
**ERR3989196**	HG03654	SAS	0.03	0.70	0.06	0.17	0.03	0.06	0.78	0.05	0.05	0.06	0.05	0.78	0.05	0.06	0.06	0.04	0.74	0.06	0.12	0.05	0.04	0.76	0.05	0.09	0.05
**ERR3989197**	HG03669	SAS	0.06	0.69	0.07	0.15	0.03	0.09	0.77	0.04	0.05	0.05	0.09	0.77	0.05	0.05	0.05	0.08	0.73	0.06	0.10	0.04	0.08	0.74	0.05	0.08	0.04
**ERR3242695**	HG03898	SAS	0.04	0.77	0.03	0.15	0.02	0.07	0.84	0.02	0.04	0.03	0.07	0.83	0.02	0.05	0.03	0.05	0.81	0.03	0.09	0.02	0.05	0.82	0.02	0.08	0.02
**ERR3989200**	HG03688	SAS	0.04	0.74	0.04	0.15	0.02	0.07	0.84	0.02	0.05	0.03	0.06	0.84	0.03	0.05	0.03	0.05	0.79	0.04	0.10	0.02	0.05	0.81	0.03	0.09	0.02
**ERR3989258**	HG04199	SAS	0.06	0.73	0.04	0.16	0.01	0.10	0.81	0.03	0.05	0.02	0.09	0.81	0.03	0.05	0.02	0.08	0.77	0.04	0.09	0.01	0.08	0.79	0.04	0.08	0.02
**ERR3989259**	HG04204	SAS	0.05	0.73	0.05	0.15	0.01	0.07	0.83	0.03	0.04	0.03	0.07	0.83	0.03	0.05	0.02	0.06	0.77	0.05	0.10	0.02	0.07	0.81	0.04	0.06	0.02
**ERR3989262**	HG04228	SAS	0.07	0.72	0.03	0.16	0.02	0.11	0.81	0.02	0.03	0.03	0.11	0.81	0.02	0.04	0.02	0.09	0.78	0.03	0.09	0.02	0.10	0.78	0.03	0.07	0.02
		**Precision**	1.00	1.00	1.00	1.00	1.00	1.00	1.00	1.00	1.00	1.00	1.00	1.00	1.00	1.00	0.95	1.00	1.00	1.00	1.00	1.00	1.00	1.00	1.00	1.00	1.00
		**Recall**	1.00	1.00	1.00	1.00	1.00	1.00	1.00	1.00	1.00	1.00	1.00	1.00	0.95	1.00	1.00	1.00	1.00	1.00	1.00	1.00	1.00	1.00	1.00	1.00	1.00
		**F1**	1.00	1.00	1.00	1.00	1.00	1.00	1.00	1.00	1.00	1.00	1.00	1.00	0.97	1.00	0.98	1.00	1.00	1.00	1.00	1.00	1.00	1.00	1.00	1.00	1.00
		**AUC**	1.00	1.00	1.00	1.00	1.00	1.00	1.00	1.00	1.00	1.00	1.00	1.00	1.00	1.00	1.00	1.00	1.00	1.00	1.00	1.00	1.00	1.00	1.00	1.00	1.00
		**Accuracy**			1.00					1.00					0.99					1.00					1.00		

Table S14. Comparison of ancestry prediction results from ADMIXTURE (AdM), SNVstory (SnvS), and ntRoot (ntR) on the balanced 1kGP WGS benchmark set of 100 samples (20 from each super-population). Reported metrics include predicted ancestry fractions, precision, recall, F1 score, AUC, and overall accuracy across all classes. For ADMIXTURE and ntRoot, the predicted global ancestry inference (GAI) label is shown. For ntRoot, the highest predicted GAI with its score is provided, along with LAI-based ancestry fraction estimates. For SNVstory, the ancestry inference probability distribution across population groups is reported. The super-populations are: EAS, East Asian; SAS, South Asian; AMR, Admixed American; AFR, African; EUR, European.

ACCESSION	ID	1kGP assigned Label	AdM GAI	ntR GAI	SnvS GAI	ntR GAI score	SnvS GAI score	AdM (ancestry fractions)					ntR (LAI-based ancestry fractions)					SnvS (GAI probabilities)				
								EAS	SAS	AMR	AFR	EUR	EAS	SAS	AMR	AFR	EUR	EAS	SAS	AMR	AFR	EUR
**ERR3242231**	HG02282	AFR	AFR	AFR	AFR	0.4410	0.9930	0.00	0.00	0.00	0.92	0.08	0.00	0.00	0.06	0.91	0.03	0.00	0.00	0.00	0.99	0.00
**ERR3989003**	HG02257	AFR	AFR	AFR	AFR	0.4419	0.9914	0.00	0.00	0.01	0.95	0.04	0.00	0.00	0.04	0.95	0.01	0.00	0.00	0.01	0.99	0.00
**ERR3242485**	HG02974	AFR	AFR	AFR	AFR	0.4441	1.0000	0.00	0.00	0.00	1.00	0.00	0.00	0.00	0.00	1.00	0.00	0.00	0.00	0.00	1.00	0.00
**ERR3989126**	HG03125	AFR	AFR	AFR	AFR	0.4419	0.9983	0.00	0.00	0.00	1.00	0.00	0.00	0.00	0.00	1.00	0.00	0.00	0.00	0.00	1.00	0.00
**ERR3989162**	HG03371	AFR	AFR	AFR	AFR	0.4421	0.9983	0.00	0.00	0.00	1.00	0.00	0.00	0.00	0.00	1.00	0.00	0.00	0.00	0.00	1.00	0.00
**ERR3989174**	HG03516	AFR	AFR	AFR	AFR	0.4431	1.0000	0.00	0.00	0.00	1.00	0.00	0.00	0.00	0.00	1.00	0.00	0.00	0.00	0.00	1.00	0.00
**ERR3242376**	HG02614	AFR	AFR	AFR	AFR	0.4450	0.9978	0.00	0.00	0.00	1.00	0.00	0.00	0.00	0.01	0.99	0.00	0.00	0.00	0.00	1.00	0.00
**ERR3242458**	HG02817	AFR	AFR	AFR	AFR	0.4444	0.9972	0.00	0.00	0.00	1.00	0.00	0.00	0.00	0.01	0.99	0.00	0.00	0.00	0.00	1.00	0.00
**ERR3989031**	HG02587	AFR	AFR	AFR	AFR	0.4443	0.9973	0.00	0.00	0.00	1.00	0.00	0.00	0.00	0.01	1.00	0.00	0.00	0.00	0.00	1.00	0.00
**ERR3989060**	HG02723	AFR	AFR	AFR	AFR	0.4416	0.9974	0.00	0.00	0.00	1.00	0.00	0.00	0.00	0.01	0.99	0.00	0.00	0.00	0.00	1.00	0.00
**ERR3989080**	HG02818	AFR	AFR	AFR	AFR	0.4436	0.9973	0.00	0.00	0.00	1.00	0.00	0.00	0.00	0.01	0.99	0.00	0.00	0.00	0.00	1.00	0.00
**ERR3989091**	HG02886	AFR	AFR	AFR	AFR	0.4419	0.9970	0.00	0.00	0.00	1.00	0.00	0.00	0.00	0.00	1.00	0.00	0.00	0.00	0.00	1.00	0.00
**ERR3239692**	NA19030	AFR	AFR	AFR	AFR	0.4441	0.9958	0.00	0.00	0.00	1.00	0.00	0.00	0.00	0.01	0.99	0.00	0.00	0.00	0.00	1.00	0.00
**ERR3239700**	NA19043	AFR	AFR	AFR	AFR	0.4421	0.9963	0.00	0.00	0.00	1.00	0.00	0.00	0.00	0.01	0.99	0.00	0.00	0.00	0.00	1.00	0.00
**ERR3242560**	HG03069	AFR	AFR	AFR	AFR	0.4377	0.9984	0.00	0.00	0.00	1.00	0.00	0.00	0.00	0.00	1.00	0.00	0.00	0.00	0.00	1.00	0.00
**ERR3242571**	HG03548	AFR	AFR	AFR	AFR	0.4390	0.9979	0.00	0.00	0.00	1.00	0.00	0.00	0.00	0.00	1.00	0.00	0.00	0.00	0.00	1.00	0.00
**ERR3989118**	HG03065	AFR	AFR	AFR	AFR	0.4409	0.9980	0.00	0.00	0.00	1.00	0.00	0.00	0.00	0.01	1.00	0.00	0.00	0.00	0.00	1.00	0.00
**ERR3989180**	HG03579	AFR	AFR	AFR	AFR	0.4412	0.9982	0.00	0.00	0.00	1.00	0.00	0.00	0.00	0.01	1.00	0.00	0.00	0.00	0.00	1.00	0.00
**ERR3239351**	NA18522	AFR	AFR	AFR	AFR	0.4444	0.9985	0.00	0.00	0.00	1.00	0.00	0.00	0.00	0.01	0.99	0.00	0.00	0.00	0.00	1.00	0.00
**ERR3239453**	NA19238	AFR	AFR	AFR	AFR	0.4420	0.9985	0.00	0.00	0.00	1.00	0.00	0.00	0.00	0.00	1.00	0.00	0.00	0.00	0.00	1.00	0.00
**ERR3242786**	HG01122	AMR	AMR	AMR	AMR	0.4800	0.9976	0.00	0.00	0.55	0.16	0.29	0.04	0.05	0.48	0.11	0.32	0.00	0.00	1.00	0.00	0.00
**ERR3242795**	HG01363	AMR	AMR	AMR	AMR	0.4642	0.9954	0.00	0.00	0.64	0.30	0.06	0.07	0.04	0.48	0.27	0.14	0.00	0.00	1.00	0.00	0.00
**ERR3242796**	HG01369	AMR	AMR	AMR	AMR	0.4764	0.9951	0.00	0.00	0.79	0.21	0.00	0.10	0.03	0.56	0.21	0.10	0.00	0.00	1.00	0.00	0.00
**ERR3242797**	HG01372	AMR	AMR	AMR	AMR	0.4878	0.9978	0.00	0.00	0.77	0.08	0.15	0.06	0.04	0.60	0.11	0.20	0.00	0.00	1.00	0.00	0.00
**ERR3242805**	HG01468	AMR	AMR	AMR	AMR	0.4902	0.9975	0.00	0.00	0.56	0.06	0.38	0.03	0.07	0.46	0.09	0.35	0.00	0.00	1.00	0.00	0.00
**ERR3988843**	HG01114	AMR	AMR	AMR	AMR	0.4892	0.9984	0.00	0.00	0.59	0.02	0.39	0.03	0.06	0.47	0.06	0.39	0.00	0.00	1.00	0.00	0.00
**ERR3988876**	HG01361	AMR	AMR	AMR	AMR	0.4833	0.9975	0.00	0.00	0.71	0.10	0.19	0.07	0.06	0.50	0.11	0.26	0.00	0.00	1.00	0.00	0.00
**ERR3988882**	HG01433	AMR	AMR	AMR	AMR	0.4784	0.9971	0.00	0.00	0.69	0.15	0.16	0.05	0.04	0.48	0.16	0.27	0.00	0.00	1.00	0.00	0.00
**ERR3988889**	HG01496	AMR	AMR	AMR	AMR	0.4763	0.9953	0.00	0.00	0.80	0.20	0.00	0.10	0.04	0.56	0.19	0.11	0.00	0.00	1.00	0.00	0.00
**ERR3989413**	NA19650	AMR	AMR	AMR	AMR	0.4825	0.9971	0.00	0.00	0.47	0.08	0.45	0.05	0.06	0.42	0.08	0.39	0.00	0.00	1.00	0.00	0.00
**ERR3242814**	HG02102	AMR	AMR	AMR	AMR	0.5069	0.9970	0.05	0.00	0.95	0.00	0.00	0.24	0.01	0.70	0.03	0.02	0.00	0.00	1.00	0.00	0.00
**ERR3988951**	HG01928	AMR	AMR	AMR	AMR	0.5134	0.9970	0.07	0.00	0.93	0.00	0.00	0.31	0.01	0.67	0.01	0.00	0.00	0.00	1.00	0.00	0.00
**ERR3988952**	HG01934	AMR	AMR	AMR	AMR	0.4951	0.9965	0.04	0.00	0.94	0.02	0.00	0.22	0.02	0.64	0.07	0.05	0.00	0.00	1.00	0.00	0.00
**ERR3988955**	HG01943	AMR	AMR	AMR	AMR	0.5068	0.9972	0.05	0.00	0.95	0.00	0.00	0.27	0.01	0.67	0.04	0.01	0.00	0.00	1.00	0.00	0.00
**ERR3988958**	HG01952	AMR	AMR	AMR	AMR	0.5044	0.9963	0.04	0.00	0.96	0.00	0.00	0.26	0.01	0.67	0.06	0.01	0.00	0.00	1.00	0.00	0.00
**ERR3988964**	HG01975	AMR	AMR	AMR	AMR	0.5031	0.9971	0.05	0.00	0.95	0.00	0.00	0.26	0.01	0.69	0.04	0.00	0.00	0.00	1.00	0.00	0.00
**ERR3241754**	HG00731	AMR	EUR	AMR	AMR	0.4852	0.9979	0.00	0.00	0.27	0.10	0.63	0.02	0.05	0.43	0.09	0.41	0.00	0.00	1.00	0.00	0.00
**ERR3241755**	HG00732	AMR	AMR	AMR	AMR	0.4815	0.9969	0.00	0.00	0.45	0.13	0.42	0.03	0.06	0.42	0.15	0.34	0.00	0.00	1.00	0.00	0.00
**ERR3242761**	HG01395	AMR	AMR	AMR	AMR	0.4776	0.9970	0.00	0.00	0.41	0.18	0.41	0.02	0.06	0.46	0.15	0.31	0.00	0.00	1.00	0.00	0.00

**ERR3242766**	HG01414	AMR	AMR	AMR	AMR	0.4674	0.9966	0.00	0.00	0.47	0.26	0.27	0.03	0.04	0.49	0.20	0.24	0.00	0.00	1.00	0.00	0.00
**ERR3242132**	HG00864	EAS	EAS	EAS	EAS	0.5315	0.9949	0.97	0.02	0.00	0.02	0.00	0.97	0.01	0.01	0.01	0.00	0.99	0.00	0.00	0.00	0.00
**ERR3242142**	HG01046	EAS	EAS	EAS	EAS	0.5328	0.9952	0.99	0.00	0.00	0.01	0.00	0.98	0.00	0.01	0.00	0.00	1.00	0.00	0.00	0.00	0.00
**ERR3242150**	HG01801	EAS	EAS	EAS	EAS	0.5303	0.9952	0.98	0.00	0.00	0.02	0.00	0.97	0.01	0.01	0.01	0.00	1.00	0.00	0.00	0.00	0.00
**ERR3242160**	HG01812	EAS	EAS	EAS	EAS	0.5333	0.9967	0.99	0.00	0.00	0.01	0.00	0.98	0.01	0.01	0.01	0.00	1.00	0.00	0.00	0.00	0.00
**ERR3241678**	HG00473	EAS	EAS	EAS	EAS	0.5303	0.9957	0.99	0.00	0.00	0.01	0.00	0.98	0.01	0.01	0.01	0.01	1.00	0.00	0.00	0.00	0.00
**ERR3241684**	HG00513	EAS	EAS	EAS	EAS	0.5309	0.9964	0.99	0.00	0.00	0.01	0.00	0.98	0.01	0.00	0.01	0.00	1.00	0.00	0.00	0.00	0.00
**ERR3241686**	HG00525	EAS	EAS	EAS	EAS	0.5353	0.9948	0.99	0.00	0.00	0.01	0.00	0.97	0.01	0.01	0.01	0.00	0.99	0.00	0.00	0.00	0.00
**ERR3241750**	HG00705	EAS	EAS	EAS	EAS	0.5356	0.9973	1.00	0.00	0.00	0.00	0.00	0.98	0.00	0.00	0.01	0.00	1.00	0.00	0.00	0.00	0.00
**ERR3242512**	HG00410	EAS	EAS	EAS	EAS	0.5308	0.9958	0.99	0.00	0.00	0.01	0.00	0.98	0.00	0.01	0.01	0.00	1.00	0.00	0.00	0.00	0.00
**ERR3242514**	HG00599	EAS	EAS	EAS	EAS	0.5318	0.9953	0.99	0.00	0.00	0.01	0.00	0.98	0.00	0.01	0.01	0.00	1.00	0.00	0.00	0.00	0.00
**ERR3242515**	HG00728	EAS	EAS	EAS	EAS	0.5334	0.9958	0.99	0.00	0.00	0.01	0.00	0.98	0.01	0.01	0.01	0.00	1.00	0.00	0.00	0.00	0.00
**ERR3242519**	HG00631	EAS	EAS	EAS	EAS	0.5332	0.9957	1.00	0.00	0.00	0.00	0.00	0.98	0.01	0.01	0.01	0.00	1.00	0.00	0.00	0.00	0.00
**ERR3242522**	HG00675	EAS	EAS	EAS	EAS	0.5332	0.9974	0.99	0.00	0.00	0.01	0.00	0.97	0.01	0.01	0.02	0.00	1.00	0.00	0.00	0.00	0.00
**ERR3988762**	HG00408	EAS	EAS	EAS	EAS	0.5320	0.9961	1.00	0.00	0.00	0.00	0.00	0.98	0.01	0.00	0.01	0.00	1.00	0.00	0.00	0.00	0.00
**ERR3988765**	HG00423	EAS	EAS	EAS	EAS	0.5323	0.9963	1.00	0.00	0.00	0.00	0.00	0.98	0.00	0.01	0.01	0.00	1.00	0.00	0.00	0.00	0.00
**ERR3988768**	HG00438	EAS	EAS	EAS	EAS	0.5324	0.9956	1.00	0.00	0.00	0.00	0.00	0.97	0.01	0.01	0.01	0.00	1.00	0.00	0.00	0.00	0.00
**ERR3988780**	HG00512	EAS	EAS	EAS	EAS	0.5329	0.9954	1.00	0.00	0.00	0.00	0.00	0.98	0.00	0.01	0.01	0.00	1.00	0.00	0.00	0.00	0.00
**ERR3242087**	HG01862	EAS	EAS	EAS	EAS	0.5303	0.9950	0.95	0.04	0.00	0.01	0.00	0.97	0.01	0.01	0.01	0.00	1.00	0.00	0.00	0.00	0.00
**ERR3242101**	HG02048	EAS	EAS	EAS	EAS	0.5309	0.9949	0.94	0.04	0.00	0.02	0.00	0.97	0.01	0.00	0.01	0.01	0.99	0.00	0.00	0.00	0.00
**ERR3242342**	HG02040	EAS	EAS	EAS	EAS	0.5297	0.9948	0.95	0.04	0.00	0.02	0.00	0.97	0.01	0.01	0.01	0.00	0.99	0.00	0.00	0.00	0.00
**ERR3239334**	NA12878	EUR	EUR	EUR	EUR	0.4951	0.9952	0.00	0.00	0.00	0.02	0.98	0.01	0.09	0.14	0.06	0.70	0.00	0.00	0.00	0.00	1.00
**ERR3240160**	HG00171	EUR	EUR	EUR	EUR	0.4950	0.9957	0.09	0.00	0.01	0.00	0.89	0.05	0.09	0.14	0.05	0.67	0.00	0.00	0.00	0.00	1.00
**ERR3240240**	HG00323	EUR	EUR	EUR	EUR	0.4950	0.9958	0.08	0.00	0.00	0.02	0.91	0.04	0.11	0.12	0.06	0.68	0.00	0.00	0.00	0.00	1.00
**ERR3241788**	HG00280	EUR	EUR	EUR	EUR	0.4932	0.9956	0.07	0.00	0.00	0.01	0.91	0.04	0.10	0.12	0.05	0.69	0.00	0.00	0.00	0.00	1.00
**ERR3241799**	HG00331	EUR	EUR	EUR	EUR	0.4934	0.9957	0.07	0.00	0.00	0.01	0.91	0.05	0.08	0.12	0.05	0.70	0.00	0.00	0.00	0.00	1.00
**ERR3240114**	HG00096	EUR	EUR	EUR	EUR	0.4950	0.9966	0.00	0.00	0.00	0.02	0.98	0.01	0.10	0.13	0.05	0.71	0.00	0.00	0.00	0.00	1.00
**ERR3240116**	HG00099	EUR	EUR	EUR	EUR	0.4947	0.9956	0.00	0.01	0.00	0.02	0.97	0.00	0.10	0.13	0.06	0.71	0.00	0.00	0.00	0.00	1.00
**ERR3240121**	HG00105	EUR	EUR	EUR	EUR	0.4949	0.9963	0.00	0.00	0.00	0.03	0.96	0.02	0.10	0.15	0.05	0.68	0.00	0.00	0.00	0.00	1.00
**ERR3240126**	HG00110	EUR	EUR	EUR	EUR	0.4981	0.9960	0.00	0.01	0.00	0.02	0.97	0.02	0.08	0.14	0.07	0.70	0.00	0.00	0.00	0.00	1.00
**ERR3240135**	HG00127	EUR	EUR	EUR	EUR	0.4965	0.9955	0.00	0.01	0.00	0.02	0.97	0.01	0.12	0.12	0.05	0.71	0.00	0.00	0.00	0.00	1.00
**ERR3240146**	HG00140	EUR	EUR	EUR	EUR	0.4961	0.9963	0.00	0.03	0.00	0.03	0.95	0.01	0.09	0.15	0.07	0.69	0.00	0.00	0.00	0.00	1.00
**ERR3240155**	HG00151	EUR	EUR	EUR	EUR	0.4941	0.9960	0.00	0.01	0.00	0.03	0.96	0.01	0.10	0.13	0.07	0.69	0.00	0.00	0.00	0.00	1.00
**ERR3240195**	HG00121	EUR	EUR	EUR	EUR	0.4976	0.9969	0.00	0.02	0.00	0.02	0.97	0.02	0.10	0.11	0.06	0.71	0.00	0.00	0.00	0.00	1.00
**ERR3242186**	HG01790	EUR	EUR	EUR	EUR	0.4960	0.9960	0.00	0.01	0.00	0.02	0.97	0.02	0.11	0.13	0.06	0.69	0.00	0.00	0.00	0.00	1.00
**ERR3241917**	HG01501	EUR	EUR	EUR	EUR	0.4875	0.9956	0.00	0.00	0.00	0.12	0.88	0.01	0.08	0.18	0.10	0.62	0.00	0.00	0.00	0.00	1.00
**ERR3241951**	HG01615	EUR	EUR	EUR	EUR	0.4903	0.9959	0.00	0.00	0.00	0.07	0.93	0.01	0.09	0.15	0.08	0.67	0.00	0.00	0.00	0.00	1.00
**ERR3242111**	HG01695	EUR	EUR	EUR	EUR	0.4832	0.9959	0.00	0.00	0.00	0.15	0.85	0.03	0.07	0.19	0.16	0.56	0.00	0.00	0.00	0.00	1.00
**ERR3988892**	HG01505	EUR	EUR	EUR	EUR	0.4943	0.9952	0.00	0.00	0.00	0.07	0.93	0.02	0.09	0.16	0.09	0.65	0.00	0.00	0.00	0.00	1.00
**ERR3239792**	NA20509	EUR	EUR	EUR	EUR	0.4920	0.9958	0.00	0.04	0.00	0.06	0.90	0.01	0.15	0.13	0.07	0.64	0.00	0.00	0.00	0.00	1.00
**ERR3239873**	NA20805	EUR	EUR	EUR	EUR	0.4933	0.9957	0.00	0.02	0.00	0.05	0.93	0.01	0.09	0.16	0.06	0.68	0.00	0.00	0.00	0.00	1.00
**ERR3989224**	HG03831	SAS	SAS	SAS	SAS	0.4910	0.9959	0.08	0.90	0.00	0.02	0.00	0.18	0.73	0.03	0.05	0.02	0.00	1.00	0.00	0.00	0.00
**ERR3989225**	HG03834	SAS	SAS	SAS	SAS	0.4906	0.9953	0.07	0.91	0.00	0.02	0.00	0.18	0.73	0.03	0.04	0.02	0.00	1.00	0.00	0.00	0.00
**ERR3240106**	NA20905	SAS	SAS	SAS	SAS	0.4901	0.9971	0.00	0.96	0.00	0.02	0.01	0.06	0.82	0.03	0.04	0.04	0.00	1.00	0.00	0.00	0.00
**ERR3243148**	HG03868	SAS	SAS	SAS	SAS	0.4933	0.9963	0.00	0.98	0.00	0.02	0.00	0.07	0.84	0.01	0.05	0.04	0.00	1.00	0.00	0.00	0.00
**ERR3989210**	HG03732	SAS	SAS	SAS	SAS	0.4929	0.9990	0.00	0.97	0.00	0.03	0.00	0.05	0.84	0.03	0.06	0.02	0.00	1.00	0.00	0.00	0.00
**ERR3242635**	HG03668	SAS	SAS	SAS	SAS	0.4919	0.9964	0.00	0.95	0.00	0.03	0.02	0.08	0.76	0.04	0.05	0.06	0.00	1.00	0.00	0.00	0.00
**ERR3243049**	HG02731	SAS	SAS	SAS	SAS	0.4909	0.9963	0.00	0.91	0.00	0.03	0.06	0.07	0.77	0.04	0.05	0.08	0.00	1.00	0.00	0.00	0.00
**ERR3243053**	HG03022	SAS	SAS	SAS	SAS	0.4942	0.9963	0.00	0.98	0.00	0.02	0.00	0.06	0.82	0.03	0.04	0.05	0.00	1.00	0.00	0.00	0.00
**ERR3989034**	HG02602	SAS	SAS	SAS	SAS	0.4916	0.9962	0.00	0.96	0.00	0.02	0.01	0.07	0.81	0.04	0.04	0.04	0.00	1.00	0.00	0.00	0.00
**ERR3989056**	HG02698	SAS	SAS	SAS	SAS	0.4913	0.9960	0.00	0.91	0.00	0.02	0.07	0.06	0.79	0.05	0.04	0.06	0.00	1.00	0.00	0.00	0.00
**ERR3989064**	HG02738	SAS	SAS	SAS	SAS	0.4911	0.9956	0.00	0.88	0.00	0.02	0.10	0.06	0.72	0.05	0.06	0.11	0.00	1.00	0.00	0.00	0.00

**ERR3989139**	HG03239	SAS	SAS	SAS	SAS	0.4902	0.9946	0.00	0.81	0.00	0.03	0.16	0.05	0.73	0.06	0.05	0.11	0.00	0.99	0.00	0.00	0.00
**ERR3989173**	HG03492	SAS	SAS	SAS	SAS	0.4896	0.9964	0.00	0.94	0.00	0.03	0.03	0.07	0.78	0.05	0.05	0.06	0.00	1.00	0.00	0.00	0.00
**ERR3989196**	HG03654	SAS	SAS	SAS	SAS	0.4897	0.9964	0.00	0.98	0.00	0.02	0.00	0.06	0.78	0.05	0.05	0.06	0.00	1.00	0.00	0.00	0.00
**ERR3989197**	HG03669	SAS	SAS	SAS	SAS	0.4914	0.9962	0.00	0.97	0.00	0.03	0.00	0.09	0.77	0.04	0.05	0.05	0.00	1.00	0.00	0.00	0.00
**ERR3242695**	HG03898	SAS	SAS	SAS	SAS	0.4939	0.9960	0.00	0.98	0.00	0.02	0.00	0.07	0.84	0.02	0.04	0.03	0.00	1.00	0.00	0.00	0.00
**ERR3989200**	HG03688	SAS	SAS	SAS	SAS	0.4917	0.9973	0.00	0.98	0.00	0.02	0.00	0.07	0.84	0.02	0.05	0.03	0.00	1.00	0.00	0.00	0.00
**ERR3989258**	HG04199	SAS	SAS	SAS	SAS	0.4927	0.9971	0.00	0.98	0.00	0.02	0.00	0.10	0.81	0.03	0.05	0.02	0.00	1.00	0.00	0.00	0.00
**ERR3989259**	HG04204	SAS	SAS	SAS	SAS	0.4917	0.9973	0.00	0.98	0.00	0.02	0.00	0.07	0.83	0.03	0.04	0.03	0.00	1.00	0.00	0.00	0.00
**ERR3989262**	HG04228	SAS	SAS	SAS	SAS	0.4929	0.9980	0.00	0.98	0.00	0.02	0.00	0.11	0.81	0.02	0.03	0.03	0.00	1.00	0.00	0.00	0.00
							**Precision**	1.00	1.00	1.00	1.00	0.95	1.00	1.00	1.00	1.00	1.00	1.00	1.00	1.00	1.00	1.00
							**Recall**	1.00	1.00	0.95	1.00	1.00	1.00	1.00	1.00	1.00	1.00	1.00	1.00	1.00	1.00	1.00
							**F1**	1.00	1.00	0.97	1.00	0.98	1.00	1.00	1.00	1.00	1.00	1.00	1.00	1.00	1.00	1.00
							**AUC**	1.00	1.00	1.00	1.00	1.00	1.00	1.00	1.00	1.00	1.00	1.00	1.00	1.00	1.00	1.00
							**Accuracy**			0.99					1.00					1.00		

We note that ntRoot (ntR) predicts both a GAI and LAI-based ancestry fraction, and the former is not derived from the latter. As such, ntRoot LAI-based fraction estimates are not expected to match the highest GAI score.

Table S15. Resource usage breakdown by steps for ADMIXTURE on the balanced set of 100, 1kGP samples (WGS benchmark set).

Step	Wall-clock run time (h)	Peak RAM (GB)
1-Making VCFs	4.42	87.35
2-Indexing VCFs	0.001715	0.030000
3-Merge Query VCFs	0.67	2.93
4-Reheader the VCF	0.13	0.06
5-Merge with ref VCF	34.44	0.14
6-Filter VCF	24.49	0.08
7-Index filtered VCF	5.83	0.02
8-plink2	2.40	14.06
9-ADMIXTURE	201.32	449.15

Benchmarks are based on the full sample set, with the exception of creating the VCFs and indexing the individual sample VCFs, which are averaged over the 100 samples.

Table S16. Key ntRoot input parameters.

Parameter	Description	Status	Default values	Rationale / Notes
**-k**	k-mer length (nt)	Optimized	55	Tested on HG002 benchmark SNVs; balances sensitivity vs precision for ancestry-discriminant SNVs
**--tile**	Genome tiling size (bp)	Optimized	5,000,000	Impacts LAI fraction calculation; empirically selected to stabilize ancestry fraction estimates
**-Y**	Bloom filter hit fraction threshold	Default	0.55	Tested on HG002 benchmark SNVs; balances sensitivity vs precision for ancestry-discriminant SNVs
**-l**	IVC VCF file input, allele frequency threshold	Fixed	Input File	IVC VCF input file to use for cross-referencing variants with ancestry-determinant SNVs. A minimum allele frequency threshold of 1% was used to discard low frequency SNVs, to reduce noise in ancestry fraction inference
**Bloom filter size**	Internal data structure for k-mers	Automatically set	Variable	BF size is designed to maintain low false positive rate (FPR: 0.008-0.0001)
**Hash functions**	Number of hash functions in Bloom filter	Set to 3 in --genome (WGA) mode	Variable	Chosen for optimal balance of memory usage and false positives
**-t Maximum threads**	Parallelization	Default	4	Default runtime configuration; can be increased for larger datasets

nt: nucleotide, SNV: Single Nucleotide Variant, bp: base pairs, IVC: Integrated Variant Call set, VCF: Variant Call Format, FPR: False Positive Rate.

Supplementary Figures

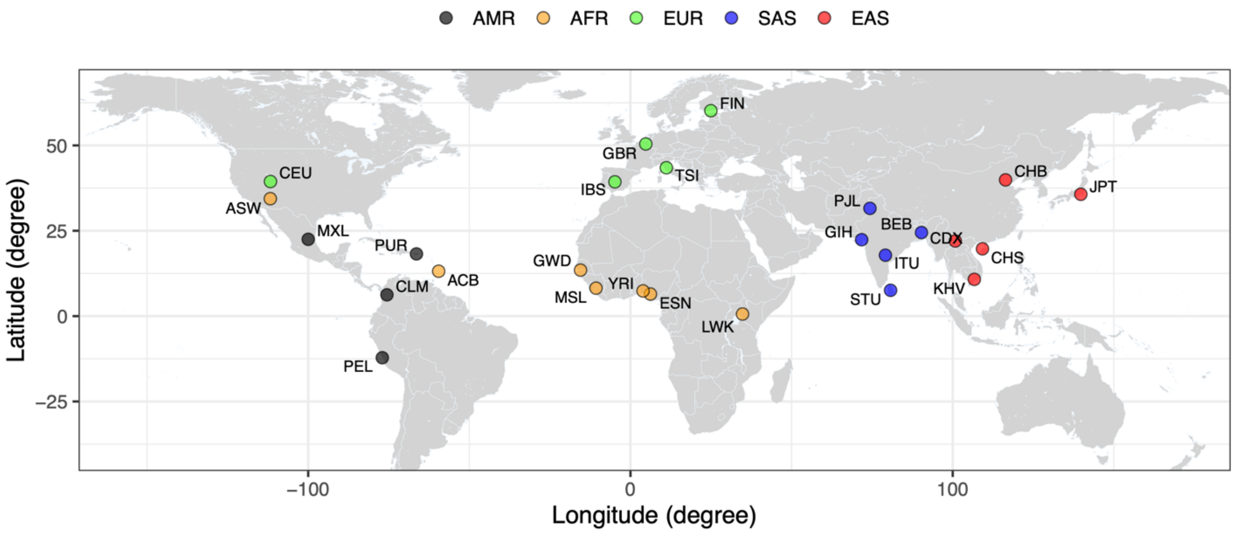

Figure S1. Geolocation of the 26 human populations, as defined by the 1000 Genomes Project. Human populations (MXL: Mexican-American, PUR: Puerto Rican, PEL: Peruvian, CLM: Colombian, ACB: African-Caribbean, ASW: African-American South West, YRI: Yoruba, GWD: Gambian, ESN: Esan, MSL: Mende, LWK: Luhya, CEU: CEPH (Centre d'Étude du Polymorphisme Humain – Utah), GBR: British, IBS: Spanish, TSI: Tuscan, FIN: Finnish, STU: Sri Lankan, BEB: Bengali, ITU: Indian, PJL: Punjabi, GIH: Gujarati, CDX: Dai Chinese, KHV: Kinh Vietnamese, CHS: Southern Han Chinese, CHB: Han Chinese, JPT: Japanese), coloured by the super-population they belong to (AMR: Admixed American, black; AFR: African, orange; EUR: European, green; SAS: South Asian, blue; EAS: East Asian, red) are shown in the global context.

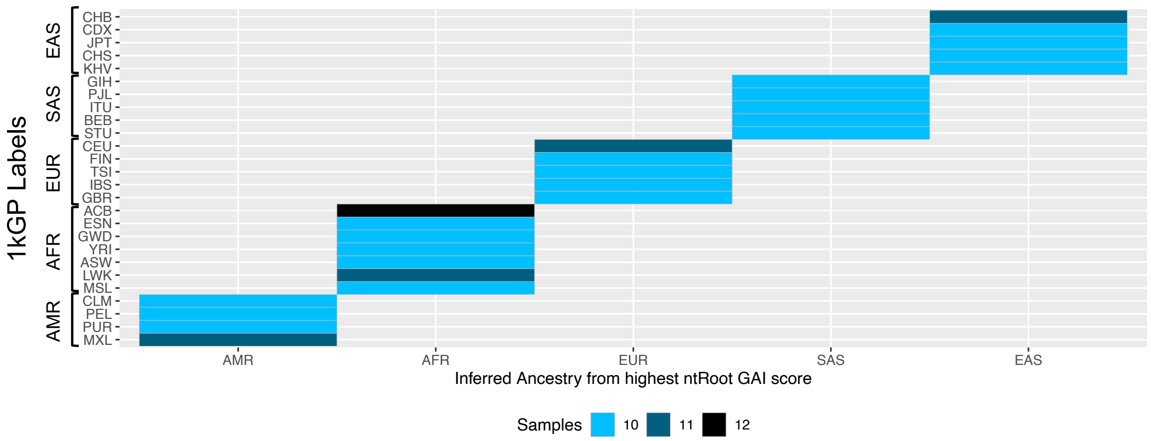

Figure S2. ntRoot super-population level GAI compared to 1kGP labels for the 1kGP WGS validation set. ntRoot super-population GAI predictions (x-axis) are compared to the 1kGP population ancestry labels (y-axis). Super-populations include EAS: East Asian; SAS: South Asian; AMR: Admixed American; AFR: African; EUR: European and are further arranged by human populations MXL: Mexican-American, PUR: Puerto Rican, PEL: Peruvian, CLM: Colombian, ACB: African-Caribbean, ASW: African-American South West, YRI: Yoruba, GWD: Gambian, ESN: Esan, MSL: Mende, LWK: Luhya, CEU: CEPH, GBR: British, IBS: Spanish, TSI: Tuscan, FIN: Finnish, STU: Sri Lankan, BEB: Bengali, ITU: Indian, PJL: Punjabi, GIH: Gujarati, CDX: Dai Chinese, KHV: Kinh Vietnamese, CHS: Southern Han Chinese, CHB: Han Chinese, JPT: Japanese. The light-to-dark blue colour indicates the number of individuals processed for each human population.

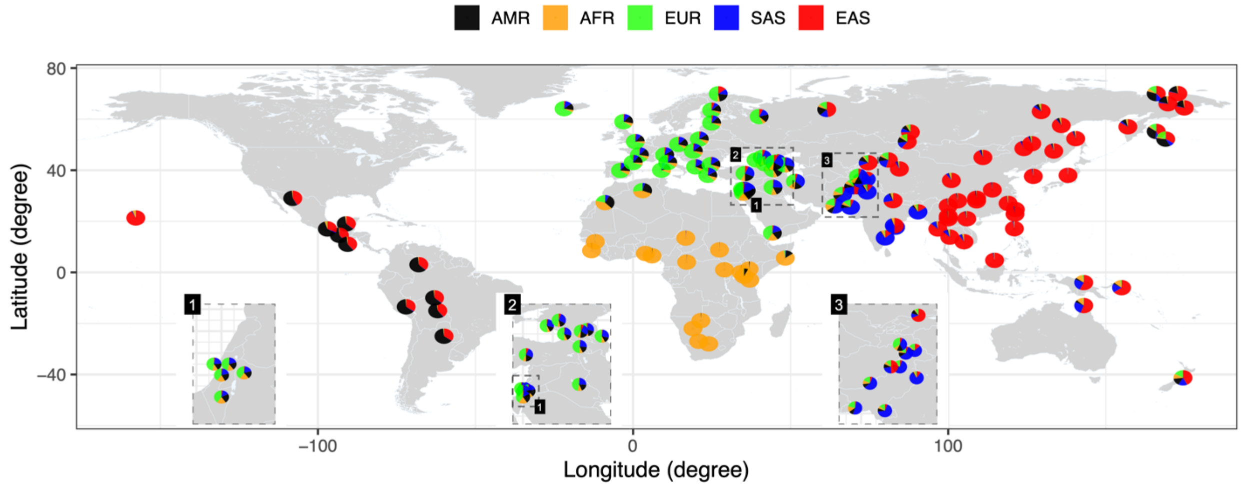

Figure S3. ntRoot ancestry composition estimates derived from LAI on SGDP WGS discovery data set from 129 distinct populations shown in the global context, without population labels and with inset regions zoomed-in. LAI-based ntRoot ancestry fraction estimates on the SGDP WGS discovery set for each population, using a representative individual randomly selected from Table S5 data, above. Ancestry fractions are coloured by super-population (AMR: Admixed American, black; AFR: African, orange; EUR: European, green; SAS: South Asian, blue; EAS: East Asian, red).

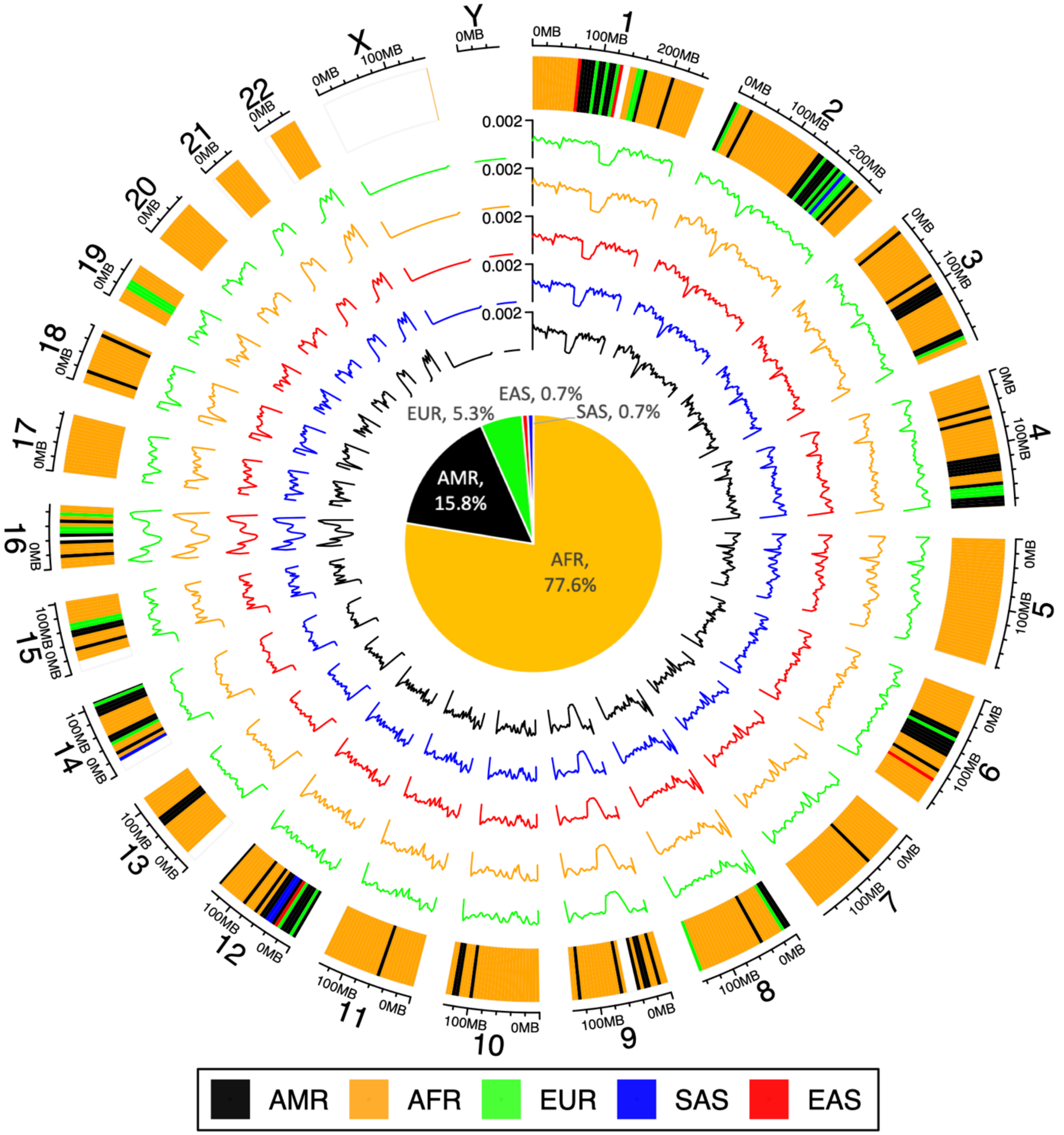

Figure S4. ntRoot LAI predictions on HG01243 Illumina whole genome sequencing data from 1kGP for a Puerto Rican individual of mixed, primarily African ancestry, labeling the likely human super-population matching each 5Mbp tile alongside the genome. The inner tracks show the SNV density (overlapping 5Mbp tiles) for EUR (European, green), AFR (African, orange), EAS (East Asian, red), SAS (South Asian, blue) and AMR (Admixed American, black). The inner pie chart shows the GAI fraction for each, the sum of each tile size assigned to a super-population over the sum of all tile sizes.

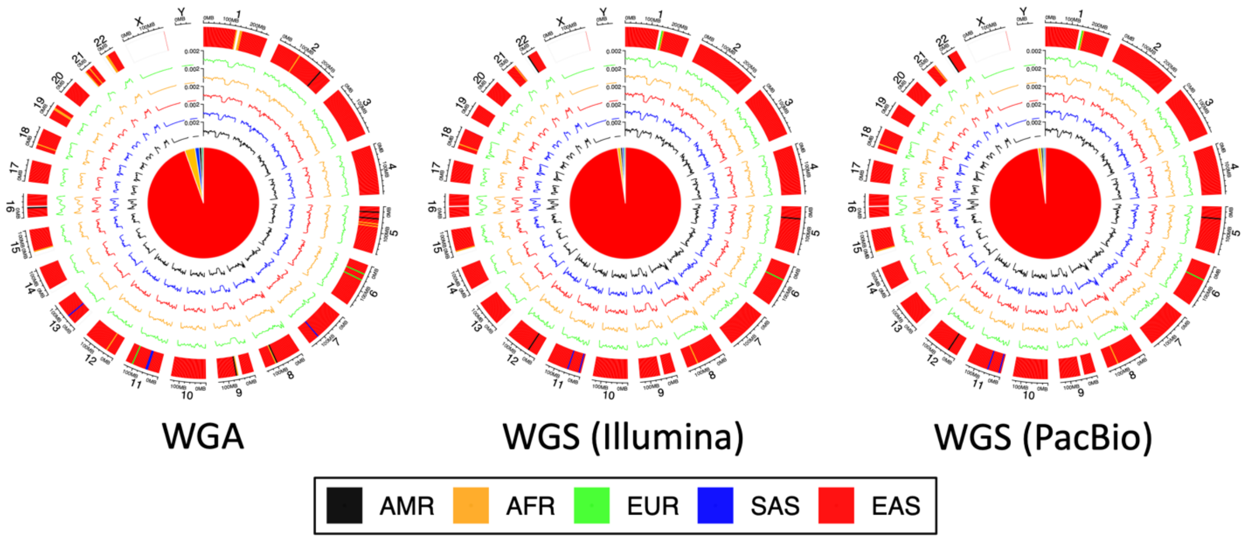

Figure S5. ntRoot LAI predictions on KOREF from a, Chromosome-scale pseudohaploid whole genome assembly reference, **b**, Illumina whole genome sequencing data and **c**, PacBio Sequel II sequencing data. LAI is shown on the outer track, labeling the likely human super-population matching each 5Mbp tile alongside the genome. The inner tracks show the SNV density (overlapping 5Mbp tiles) for EUR (European, green), AFR (African, orange), EAS (East Asian, red), SAS (South Asian, blue) and AMR (Admixed American, black). The inner pie chart shows the GAI fraction for each, the sum of each tile size assigned to a super-population over the sum of all tile sizes.

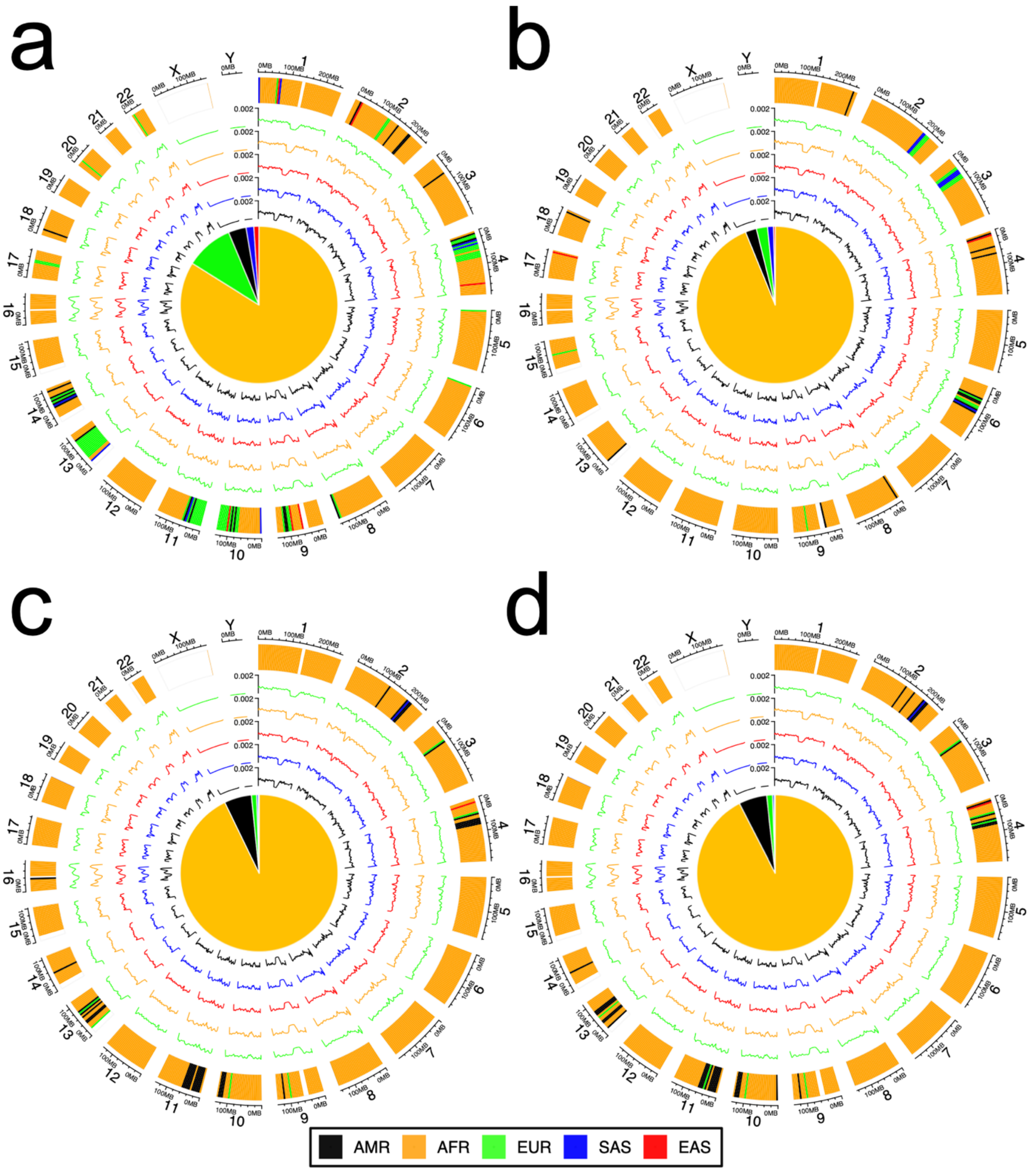

Figure S6. ntRoot LAI predictions on HG02055 directly on **a,** Maternal and **b,** paternal chromosome-complete haploid reference genomes and **c,** Flye and **d,** Shasta pseudohaploid genome assembly drafts. LAI is shown on the outer track, labeling the likely human super-population matching each 5Mbp tile alongside each genome. The inner tracks show the SNV density (overlapping 5Mbp tiles) for EUR (European, green), AFR (African, orange), EAS (East Asian, red), SAS (South Asian, blue) and AMR (Admixed American, black). The inner pie chart shows the GAI fraction for each, the sum of each tile size assigned to a super-population over the sum of all tile sizes.

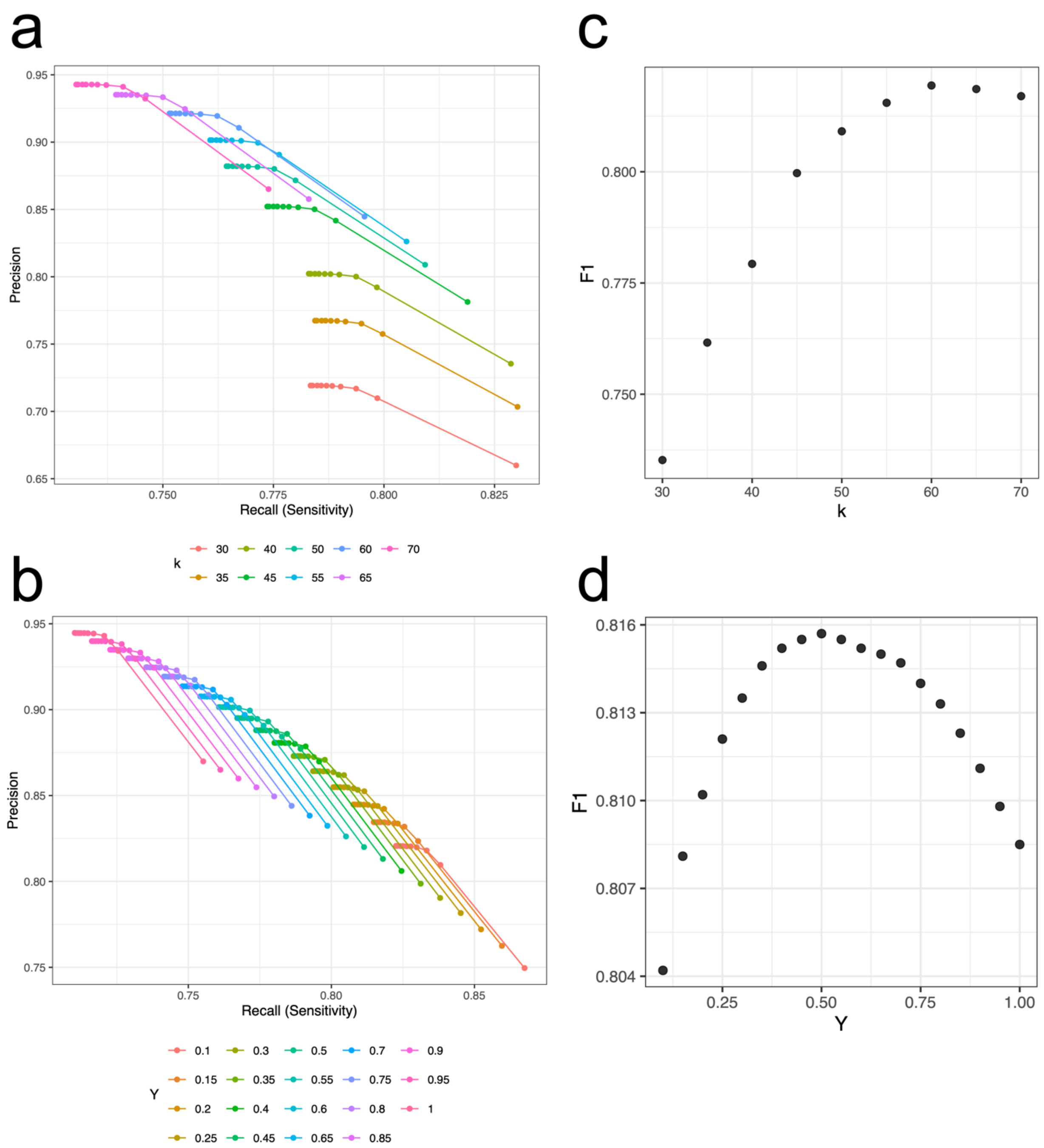

Figure S7. Selection of ntRoot parameters k and Y based on precision-recall and F1 score analyses. Precision–Recall curves across (a) k and (b) Y values, and F1 score across (c) k and (d) Y values for identifying ancestry-discriminant SNVs relative to the Genome in a Bottle HG002 benchmark set[5], which integrates results from multiple sequencing platforms and variant callers. Each datapoint represents ntRoot's agreement with between 1 and 10 benchmark callsets (from left to right); in (a) and (b), colors indicate k and Y values, respectively.

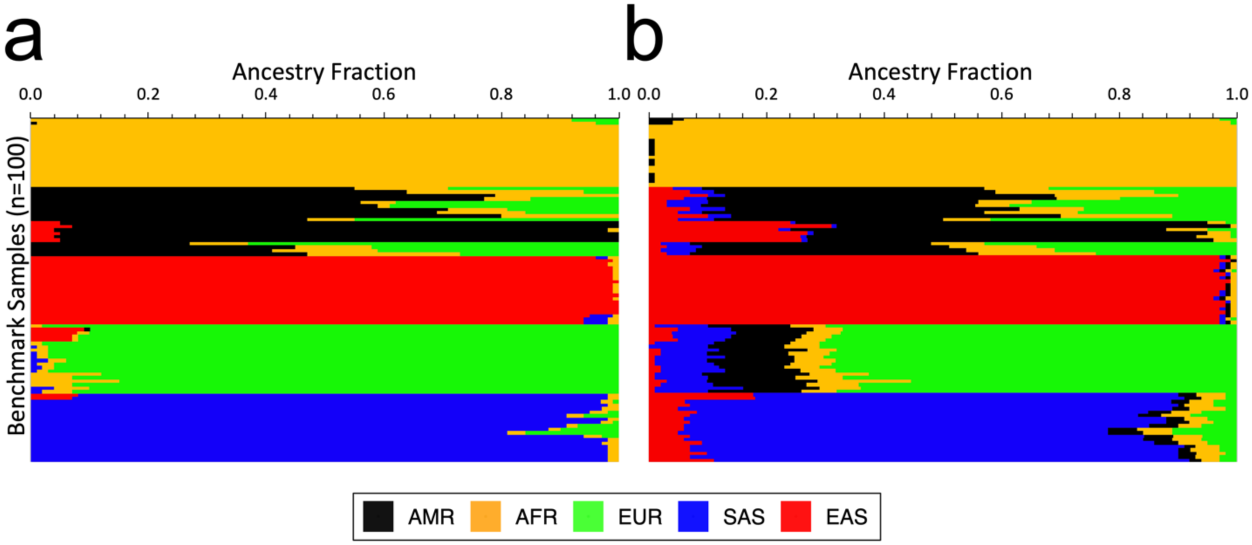

Figure S8. a, ADMIXTURE and b, ntRoot ancestry fraction predictions (y-axis) on the balanced WGS benchmark set of 100, 1kGP samples (x-axis, matching the order of Table S10 from top to bottom). The fractions are represented as horizontal stacked bars for each super-population label: EUR (European, green), AFR (African, orange), EAS (East Asian, red), SAS (South Asian, blue), and AMR (Admixed American, black).

References

1. 1000 Genomes Project Consortium. A global reference for human genetic variation. *Nature* **526**, 68 (2015).
2. Byrska-Bishop, M. *et al.* High-coverage whole-genome sequencing of the expanded 1000 Genomes Project cohort including 602 trios. *Cell* **185**, 3426-3440.e19 (2022).
3. Mallick, S. *et al.* The Simons Genome Diversity Project: 300 genomes from 142 diverse populations. *Nature* **538**, 201–206 (2016).
4. Wong, J. *et al.* Linear time complexity de novo long read genome assembly with GoldRush. *Nat. Commun.* **14**, 2906 (2023).
5. Zook, J. M. *et al.* An open resource for accurately benchmarking small variant and reference calls. *Nat. Biotechnol.* **37**, 561–566 (2019).
6. Bollas, A. E. *et al.* SNVstory: inferring genetic ancestry from genome sequencing data. *BMC Bioinformatics* **25**, 76 (2024).
7. Li, H. Aligning sequence reads, clone sequences and assembly contigs with BWA-MEM. Preprint at https://doi.org/10.48550/ARXIV.1303.3997 (2013).
8. McKenna, A. *et al.* The Genome Analysis Toolkit: a MapReduce framework for analyzing next-generation DNA sequencing data. *Genome Res.* **20**, 1297–1303 (2010).
9. Van der Auwera, G. A. & O'Connor, B. D. *Genomics in the Cloud: Using Docker, GATK, and WDL in Terra*. (O'Reilly Media, Inc., 2020).
10. Danecek, P. *et al.* Twelve years of SAMtools and BCFtools. *GigaScience* **10**, giab008 (2021).

11. Alexander, D. H., Novembre, J. & Lange, K. Fast model-based estimation of ancestry in unrelated individuals. *Genome Res.* **19**, 1655–1664 (2009).

12. Chang, C. C. *et al.* Second-generation PLINK: rising to the challenge of larger and richer datasets. *Gigascience* **4**, s13742-015-0047–8 (2015).

13. Levy, S. *et al.* The Diploid Genome Sequence of an Individual Human. *PLoS Biol.* **5**, e254 (2007).

14. Yang, C. *et al.* The complete and fully-phased diploid genome of a male Han Chinese. *Cell Res.* **33**, 745–761 (2023).

15. Kim, H.-S. *et al.* KOREF_S1: phased, parental trio-binned Korean reference genome using long reads and Hi-C sequencing methods. *GigaScience* **11**, giac022 (2022).

16. Liao, W.-W. *et al.* A draft human pangenome reference. *Nature* **617**, 312–324 (2023).
